# Role of moesin in hyaluronan induced cell migration in glioblastoma multiforme

**DOI:** 10.1186/1476-4598-12-74

**Published:** 2013-07-15

**Authors:** Leroi V DeSouza, Ajay Matta, Zia Karim, Joydeep Mukherjee, X Simon Wang, Olga Krakovska, Gelareh Zadeh, Abhijit Guha, KW Michael Siu

**Affiliations:** 1 Department of Chemistry and Centre for Research in Mass Spectrometry, York University, 4700 Keele Street, M3J 1P3, Toronto, Ontario, Canada; 2 Arthur and Sonia Labatt Brain Tumor Research Center, The Hospital for Sick Children Research Institute, University of Toronto, Toronto, Canada

**Keywords:** Glioblastoma, CD44, ERM proteins, Moesin

## Abstract

**Background:**

A major barrier to effective treatment of glioblastoma multiforme (GBM) is the invasion of glioma cells into the brain parenchyma rendering local therapies such as surgery and radiation therapy ineffective. GBM patients with such highly invasive and infiltrative tumors have poor prognosis with a median survival time of only about a year. However, the mechanisms leading to increased cell migration, invasion and diffused behavior of glioma cells are still poorly understood.

**Methods:**

In the current study, we applied quantitative proteomics for the identification of differentially expressed proteins in GBMs as compared to non-malignant brain tissues.

**Results:**

Our study led to the identification of 23 proteins showing overexpression in GBM; these include membrane proteins, moesin and CD44. The results were verified using Western blotting and immunohistochemistry in independent set of GBM and non-malignant brain tissues. Both GBM tissues and glioma cell lines (U87 / U373) demonstrated membranous expression of moesin and CD44, as revealed by immunohistochemistry and immunofluorescence, respectively. Notably, glioma cells transfected with moesin siRNA displayed reduced migration and invasion on treatment with hyaluronan (HA), an important component of the extracellular matrix in GBM. CD44, a transmembrane glycoprotein, acts as a major receptor for hyaluronan (HA). Using co-immunoprecipitation assays, we further demonstrated that moesin interacts with CD44 in glioma cells only after treatment with HA; this implicates a novel role of moesin in HA-CD44 signaling in gliomas.

**Conclusions:**

Our results suggest that development of inhibitors which interfere with CD44-moesin interactions may open a new avenue in the future to mitigate cellular migration in gliomas.

## Background

Glioblastoma multiforme (GBM) is the most common primary adult brain tumor and is almost always lethal due to persistent local recurrence and invasion into the surrounding tissue. Malignant gliomas are highly invasive and infiltrative tumors that have a poor prognosis with a median survival of only about one year [1,2]. The Canadian Cancer Society estimates an incidence rate of 2,800 new cases of brain cancer and 1,850 deaths from it in Canada for the year 2012 [3]. A major barrier to effective treatment of GBM patients is invasion of cancer cells into the brain parenchyma, rendering local therapies such as surgery and radiation therapy ineffective. However, the mechanisms leading to increased cell invasion and diffused behavior of glioma cells are still unclear [3,4]. Hyaluronan (HA), a principal glycosaminoglycan (GAG) and an important component of the extracellular matrix (ECM), is involved in the development, proliferation, migration, invasion and therapeutic resistance observed in cancer cells [5,6]. Higher amounts of HA have been reported in ECM of GBM patients than that in normal brain tissue. Abundant amounts of HA in gliomas have been associated with poor prognosis in GBM patients [5,6]. Thus, identification of the proteins and their roles within this niche may lead to better understanding of the behavior of glioma cells and provide a basis for deciphering the mechanisms involved in invasion and proliferation.

Recently, high-throughput quantitative proteomic analysis using isobaric tags for relative and absolute quantitation (iTRAQ) in combination with liquid chromatography-tandem mass spectrometry (LC-MS/MS) has been applied to the discovery of potential candidate markers for a variety of malignancies [7-13]. Such studies have included our own reports on endometrial carcinoma (EmCa), head and neck squamous cell carcinoma (HNSCC), oral premalignant lesions (OPL), and renal cell carcinoma (RCC) [7-13]. In the current study, we applied a similar approach for identification of proteins and protein-protein interaction networks that may be responsible for the highly invasive and lethal nature of GBMs. Our study reveals 57 differentially expressed proteins (23 upregulated and 34 downregulated) in GBM tissue samples as compared to non-malignant brain tissues. Importantly, we observe in GBM tissues the overexpression of moesin, a member of the ERM (ezrin, radixin, moesin) family of proteins, and CD44, an important HA-receptor on cell membrane. The ERM family of proteins functions as molecular cross-linkers between plasma membrane receptors and cytoplasmic actin filaments [14]. Notably in our iTRAQ analysis, moesin shows overexpression while both ezrin and radixin show no significant differential expression in GBMs in comparison to non-malignant brain tissue samples. CD44 is a multifunctional, transmembrane glycoprotein showing overexpression in a variety of malignancies and serves as the major receptor for hyaluronan (HA). The HA-CD44 interaction potentially mediates invasion, migration, and resistance to chemotherapy in several malignancies [15,16]. However, the mechanisms underlying HA-CD44 induced migration, invasion and therapeutic resistance in gliomas is poorly understood. This provided us the impetus to examine the role played by moesin, downstream of HA-CD44 signaling in glioma cells.

## Results

### Identification of differentially expressed proteins using quantitative proteomics

Tissues lysates prepared from GBM and non-malignant brain tissues were labeled with respective iTRAQ labels for the identification and quantification of differentially expressed proteins (Additional file 1: Table S1). Using the 0.1% global FDR as cutoff, analysis of the two runs of each set led to identification of 422 proteins in the first set, 331 in the second set, and 310 in the third set. In total, 753 non-redundant proteins were identified across all three sets (see Additional file 2: Table S2). Proteins that displayed expression levels altered by more than 50% in at least three out of the six GBMs in comparison to both non-malignant brain tissue samples were considered as differentially expressed. Following these criteria, only 57 of 753 identified proteins were considered differential expression in GBM. Of these, 23 proteins were upregulated, while 34 proteins were downregulated (Table 1). An ERM protein, moesin; stem cell markers, CD44 and nestin; and calcium-binding proteins of the S100 family, S100A11 and S100A6 were upregulated in GBM tissues. Moesin overexpression was observed in all six GBM tissues, while CD44 overexpression was observed in four of six GBM tissues (Table 1). Intriguingly, three of the seven isoforms of the 14-3-3 family, including 14-3-3 zeta, gamma, epsilon, and metabolic enzymes such as gamma enolase were downregulated (Table 1).

**Table 1:** List of differentially expressed proteins in GBM and non-malignant brain tissue samples

**Accession #**	**Name**	**NB1**	**NB4**	**NB5**	**GBM1**	**GBM2**	**GBM3**	**GBM4**	**GBM5**	**GBM6**
sp|P21333-2	Isoform 2 of filamin-A	1.46	1.37	0.33	5.20	7.24	3.31	7.80	7.66	7.87
sp|Q15084	Protein disulfide-isomerase A6	1.29	0.23	1.10	2.51	2.07	2.61	1.91	1.92	1.57
sp|P04792	Heat shock protein beta-1	0.95	0.96	1.45	6.19	2.78	2.47	2.96	1.80	2.33
sp|P04083	Annexin A1	1.06	0.86	1.96	3.63	1.61	3.31	3.47	30.48	28.31
sp|P06703	Protein S100-A6	0.94	1.04	2.03	10.86	4.06	9.73	3.53	6.03	10.47
sp|P26038	**Moesin**	1.29	1.04	1.51	2.65	2.03	2.54	4.74	4.70	12.36
sp|P16070	**CD44 antigen**		1.09	5.97	5.30		5.75		7.59	15.00
sp|P48681	Nestin	1.05	0.42	2.78	9.64	2.17	11.59	2.70	8.47	6.14
sp|P27797	Calreticulin	4.79	1.21	1.24	11.38	20.32	9.64	18.20	17.22	8.47
sp|P08758	Annexin A5	0.79	0.33	9.91	5.97	0.67	7.45	2.70	24.43	12.02
sp|P13667	Protein disulfide-isomerase A4	0.87	0.82	0.68	1.67	0.92	1.92	0.79	4.13	2.09
sp|P23284	Peptidyl-prolyl cis-trans isomerase B		0.69	0.80	7.38		8.55		12.82	4.61
sp|P17931	Galectin-3		0.96	1.07	9.73		12.02		2.61	1.75
sp|Q05682	Caldesmon	1.11	0.96		2.29	1.77	2.01	1.66		
sp|P02763	Alpha-1-acid glycoprotein 1	1.31	0.50	0.02	11.70	9.38	7.24	5.40	0.81	0.82
sp|P01023	Alpha-2-macroglobulin	1.49	0.76	0.06	2.75	3.50	2.96	2.13	1.28	0.54
sp|P02787	Serotransferrin	2.73	0.59	0.03	10.67	12.47	10.19	4.70	0.82	0.33
sp|P01024	Complement C3	1.54	0.50	0.03	4.92	5.55	5.30	2.13	0.65	0.46
sp|P02647	Apolipoprotein A-I	2.36	1.20	0.03	8.79	15.56	7.59	6.03	0.37	0.29
sp|P31949	Protein S100-A11		1.25	9.73	14.59		18.03		23.99	20.14
sp|P61604	10 kDa heat shock protein, mitochondrial	0.54	0.90	1.04	2.38	1.92	3.77	1.26	1.00	0.44
sp|P30101	Protein disulfide-isomerase A3	1.71	3.10	1.32	13.18	2.11	16.29	2.42	9.82	5.70
sp|P14625	Endoplasmin	4.49	0.84	8.87	5.20	3.19	6.67	2.91	21.48	11.48
sp|Q9BY11	Protein kinase C and casein kinase substrate in neurons protein 1	0.81	1.28	0.89	0.09	0.24	0.14	0.35	0.16	0.11
sp|Q13885	Tubulin beta-2A chain	1.34	0.67	0.77	0.05	0.11	0.06	0.36	0.19	0.31
sp|P17612	cAMP-dependent protein kinase catalytic subunit alpha	0.90	1.14	1.46	0.49	0.55	0.06	0.52	0.61	0.62
sp|P63104	14-3-3 protein zeta/delta	1.07	0.70	1.46	0.25	0.18	0.13	0.53	0.53	0.38
sp|P61981	14-3-3 protein gamma	1.10	1.07	0.65	0.46	0.21	0.44	0.25	0.83	0.39
sp|P62258	14-3-3 protein epsilon	0.96	0.96	2.36	0.51	0.58	0.51	0.83	0.63	0.92
sp|Q92686	Neurogranin	1.42	0.72	2.31	0.21	0.03	0.37	0.27	0.02	0.02
sp|P31150	Rab GDP dissociation inhibitor alpha	0.96	0.72	2.36	0.16	0.52	0.10	0.38	0.19	0.23
sp|P62158	Calmodulin	1.31	0.85	2.42	0.25	0.04	0.18	0.15	0.34	0.06
sp|P62988	Ubiquitin	0.77	0.75	2.88	0.46	0.53	0.60	0.60	0.42	0.53
sp|P11142	Heat shock cognate 71 kDa protein	1.18	0.82	2.27	0.36	0.43	0.37	0.53	0.15	0.43
sp|P49773	Histidine triad nucleotide-binding protein 1	1.37	0.81	1.79	0.37	0.45	0.50	0.57	0.49	0.58
sp|P16949	Stathmin	1.13	1.05	2.27	0.74	0.37	0.64	0.17	0.18	0.65
sp|P12277	Creatine kinase B-type	0.89	0.86	5.65	0.04	0.26	0.04	0.21	0.15	0.85
sp|P04350	Tubulin beta-4 chain	0.96	0.68	1.87	0.08	0.26	0.09	0.67	0.27	0.07
sp|Q01813	6-phosphofructokinase type C	1.04	0.82	1.00	0.60	0.46	0.70	0.52	0.53	0.44
sp|Q13509	Tubulin beta-3 chain	0.96	0.79	0.21	0.11	0.10	0.09	0.35	0.30	0.32
sp|P09104	Gamma-enolase	0.68	0.44	1.50	0.05	0.07	0.04	0.16	0.10	0.07
sp|P04075	Fructose-bisphosphate aldolase A	0.78	0.63	1.09	0.50	0.25	0.51	0.17	0.61	0.30
sp|P09972	Fructose-bisphosphate aldolase C	0.83	0.67	1.08	0.08	0.44	0.05	0.23	0.14	0.14
sp|Q96JE9	Microtubule-associated protein 6	0.86	0.50	1.18	0.02	0.09	0.04	0.18	0.07	0.06
sp|P09936	Ubiquitin carboxyl-terminal hydrolase isozyme L1	1.22	0.59	3.31	0.10	0.15	0.07	0.54	0.52	0.25
sp|P30086	Phosphatidylethanolamine-binding protein 1	1.07	0.42	2.25	0.22	0.24	0.19	0.30	0.07	0.03
sp|P10636	Isoform Tau-E of microtubule-associated protein tau	1.32	0.60	1.56	0.09	0.45	0.05	0.39	0.02	0.03
sp|P00505	Aspartate aminotransferase, mitochondrial	0.68	0.96	0.34	0.14	0.70	0.21	0.44	0.12	0.08
sp|P00441	Superoxide dismutase [Cu-Zn]	1.32	0.55	0.94	0.18	0.59	0.11	0.76	0.31	0.35
sp|P21291	Cysteine and glycine-rich protein 1	1.80	0.66	3.22	0.26	0.22	0.41	1.06	0.09	0.56
sp|P07437	Tubulin beta chain	0.93	1.09	1.22	0.20	0.64	0.20	0.39	0.22	3.02
sp|P09543	2',3'-cyclic-nucleotide 3'-phosphodiesterase	1.10	0.28	1.32	0.07	0.26	0.09	2.03	0.07	0.03
sp|O76070	Gamma-synuclein	2.83	0.38	0.58	0.21	0.48	0.10	1.66	0.12	0.04
sp|Q99962	Endophilin-A1	0.80	0.70		0.52	0.21	0.58	0.15		
sp|P38606	V-type proton ATPase catalytic subunit A	0.83	0.76		0.29	0.11	0.24	0.33		
sp|Q99719	Septin-5	0.72	0.87		0.24	0.38	0.23	0.39		
sp|P35611	Alpha-adducin	0.84	0.98		0.19	0.47	0.05	0.60		
sp|P17174	Aspartate aminotransferase, cytoplasmic	1.03	0.82	1.51	0.65	0.88	0.59	0.80	0.19	0.08

Values shown in the table are the ratios for each of the proteins relative to its expression level in the designated individual non-malignant sample in each set. Thus NB1, GBM1 and GBM2 are relative to NB2; NB4, GBM3 and GBM4 are relative to NB3 and NB5, GBM5 and GBM6 are relative to NB6. The missing values in the table represent proteins that were either not detected in that set or not reliably quantified in iTRAQ experiments.

### Verification of differentially expressed proteins using western blotting and immunohistochemistry (IHC)

**Figure 1:**
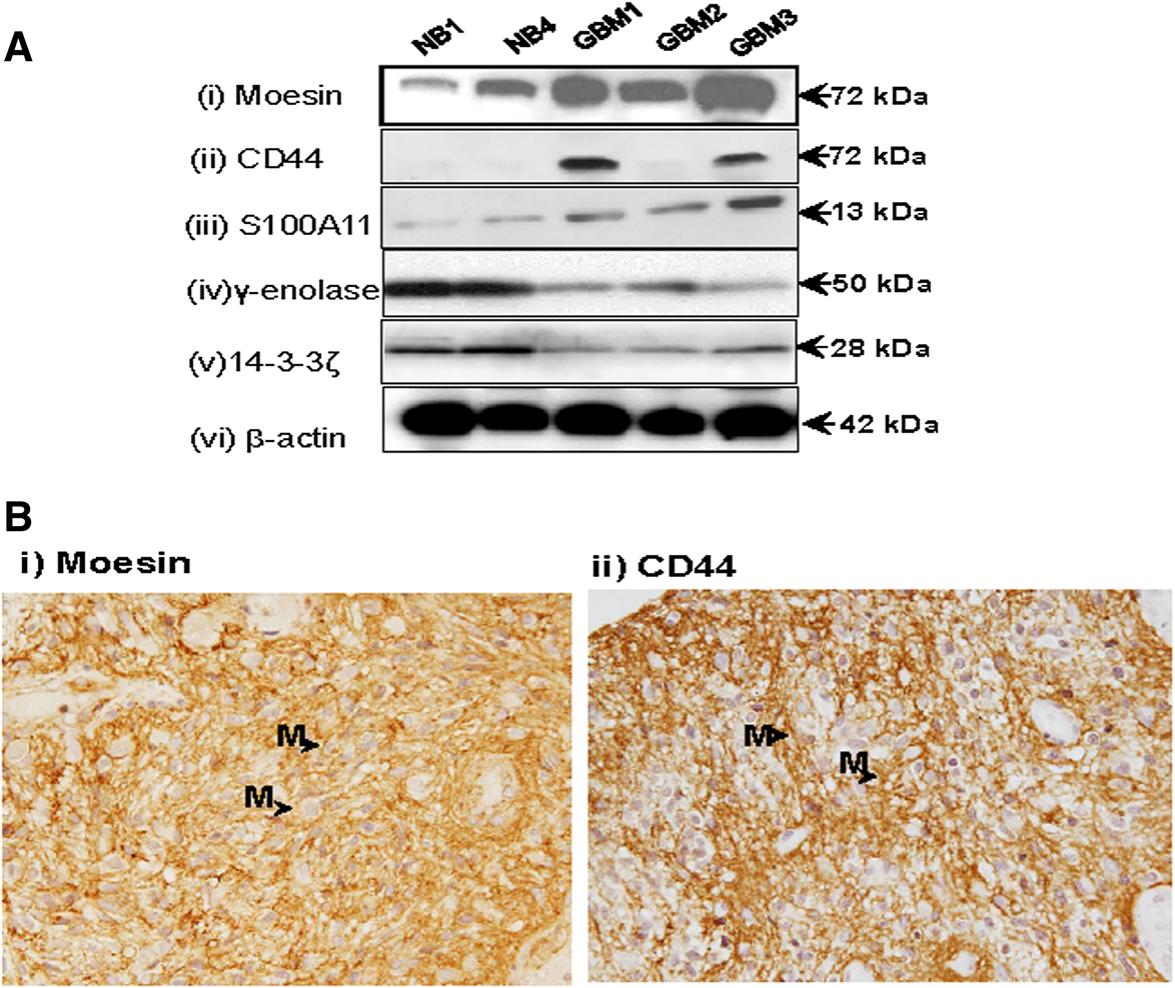
**Verification of moesin and CD44 overexpression in GBMs. (A)** Western blot analysis was carried out using specifics antibodies for moesin, CD44, S100A11, 14-3-3ζ and γ-enolase and β-actin as described in Materials and Methods. Panel **(i)** shows increased expression of moesin in GBM tissues (GBM1, GBM2 & GBM3) in comparison to non-malignant brain tissues (NB1 & NB4); **(ii)** CD44 overexpression in GBMs (GBM1 & GBM3) as compared to non-malignant brain tissues (NB1 & NB4); **(iii)** S100A11 overexpression in all GBM tissues (GBM1, GBM2 & GBM3) in comparison to non-malignant brain tissues (NB1 & NB4), whereas panels **(iv)** and **(v)** and **(v)** showed downregulation of γ-enolase and 14-3-3ζ respectively in in GBM tissues as compared to non-malignant brain tissues, thereby verifying our iTRAQ results; **(iii)** β-actin was used as a loading control; **(B)** Immunohistochemistry (IHC) was performed on independent set paraffin embedded sections of GBM tissues using monoclonal antibodies (Moesin / CD44). IHC analysis showed strong membranous expression of **(i)** Moesin and **(ii)** CD44 in glioma cells. Arrows shows membranous expression of moesin / CD44 in GBM tissue sections (Original magnification X400).

Tissues lysates from an independent set of GBM (n = 3) and non-malignant brain tissues (n = 2) were used for Western blotting to confirm differential expression of CD44, moesin, S100A11, 14-3-3ζ and γ-enolase. Western blot analysis showed overexpression of moesin, CD44 and S100A11 in at least two of the three GBM tissues (GBM1 and GBM3) as compared to non-malignant brain tissues (NB1 and NB4, Figure 1A). Further, both 14-3-3ζ and γ-enolase showed lower expression in GBM tissues as compared to non-malignant brain tissues, thereby verifying our iTRAQ data (Figure 1A). The results for moesin and CD44 overexpression were further verified in a second independent set of paraffin-embedded sections of GBM tissues (n = 15) using IHC. Twelve of 15 GBM samples showed strong moesin expression on the membrane of glioma cells, while eight of 15 cases showed strong membranous CD44 expression (Figure 1B). In each tissue microarray used in this study, liver sections were used as positive controls. In liver sections, both CD44 and moesin showed faint cytoplasmic and moderate to strong membranous immunostaining for moesin and CD44 (data not shown). In negative controls, no immunostaining was observed in either cytoplasm / membrane in glioma cells (data not shown).

### Effect of HA-treatment on migration, proliferation and cell cycle in glioma cells

**Figure 2:**
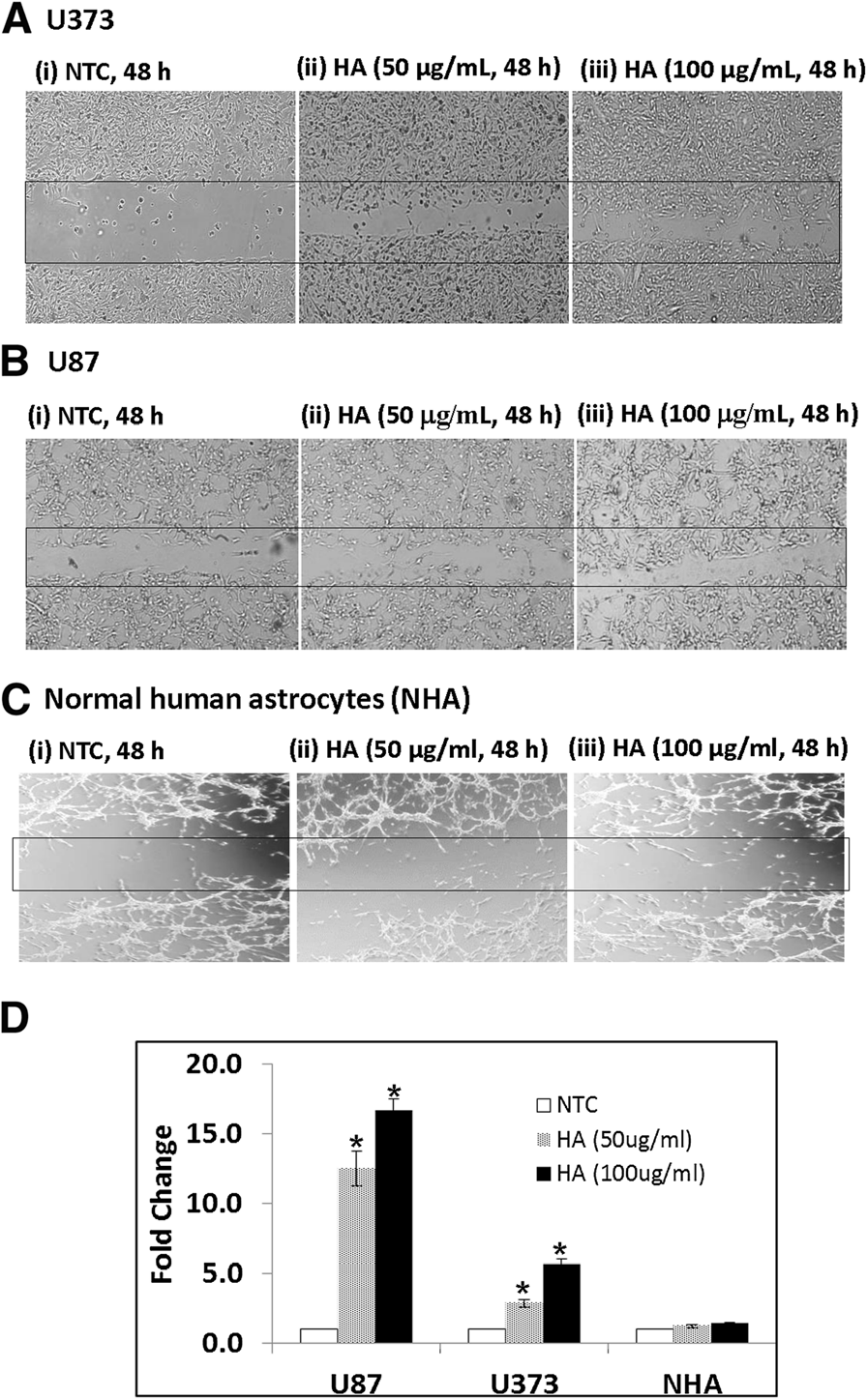
**Effect of HA-treatment on cell migration.** Glioma cells (U373 and U87) and normal human astrocytes (NHA) were plated and treated with HA (50 – 100 μg/mL) for 48 h. All HA-treatments were given in DMEM only. Both glioma cell lines **(A)** U373 and **(B)** U87 showed increase cell migration when treated with HA (50 - 100 μg/mL) as compared to no treatment controls (NTC); **(C)** Normal human astrocytes (NHA) treated with HA (50 - 100 μg/mL) showed no significant difference as compared with no treatment control (NTC). Panel **(D)** shows bar graph representing fold change ± standard deviation (S.D.) of the number of cells in the wound for on HA-treatment as compared to no treatment controls (n = 3, *p < 0.05). (Original magnification X40).

Our IHC results revealed an exclusive strong membranous expression of both CD44 and moesin in glioma cells in paraffin-embedded sections of GBM tissues. As CD44 is a receptor for hyaluronan (HA), and was identified and verified as overexpressed in GBM tissues, we explored the effects of HA-treatment on cell migration, proliferation, and cell cycle in glioma cells (U87 / U373) as well as normal human astrocytes (NHA). Both, glioma cells and NHA were treated with HA (50 – 100 μg/mL) for up to 48 h to determine the effect of treatment on cell migration using wound-healing assays. Wound closure (i.e. width of the wound) was evaluated from 6 - 48 h. Our results demonstrated increased cell migration (i.e. reduced width of the wound) in both glioma cell lines, but not in NHA upon treatment with HA (100 μg/mL, 48 h, Figure 2A - 2D). No significant difference in the size of the wound was observed in HA-treated glioma cells (U87 / U373) in comparison to no treatment controls until 24 h (Additional file 3: Figure S1A). Glioma cell lines treated with TNF-α (10 nM, used as positive control in wound-healing assays) showed decrease in size of the wound as early as 12 h (Additional file 3: Figure S1B). Further, HA-treated cells and no treatment controls demonstrated no significant difference in cell proliferation (50 – 200 μg/mL, 24 – 48 h), as determined by MTT assay (Additional file 4: Figure S2A). Moreover, treatment with HA (100 μg/mL, 48 h) showed no difference in percentage fraction of cells in each phase of the cell cycle in glioma cell lines in comparison to no treatment controls (Additional file 4: Figure S2B).

### Effect of HA-treatment on CD44 and moesin in glioma cells

**Figure 3:**
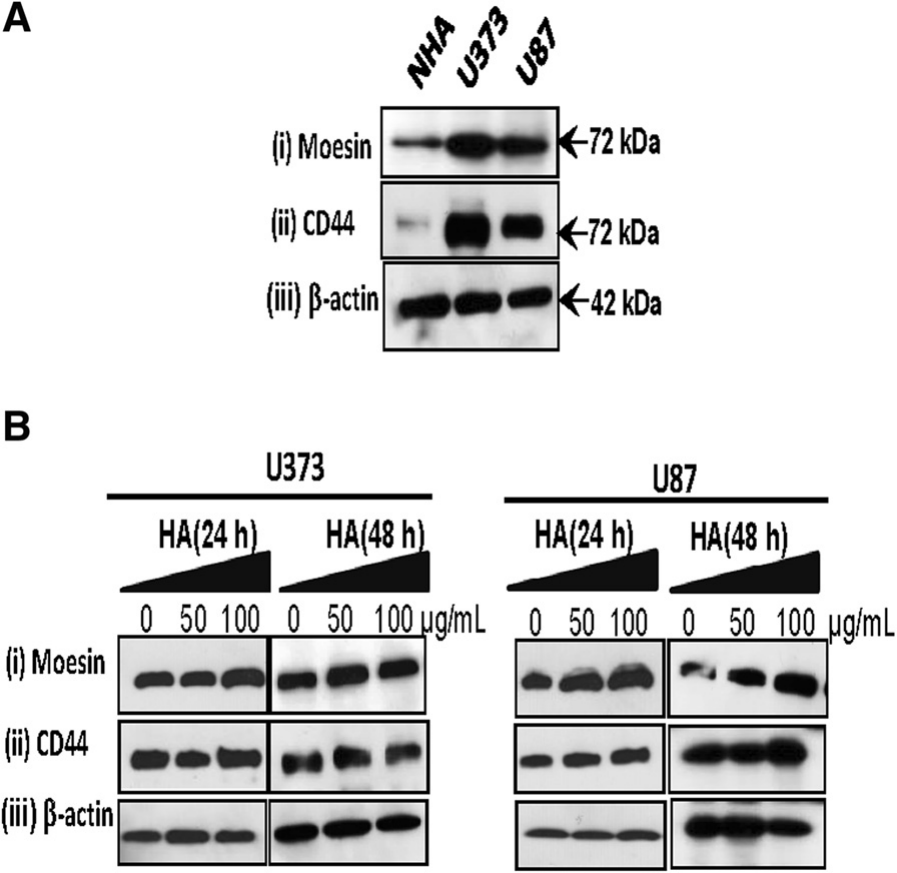
**Effect of HA-treatment on moesin and CD44 in glioma cells (U373 / U87). (A)** Panel shows western blot analysis demonstrating increased expression of both **(i)** moesin and **(ii)** CD44 in glioma cells (U87 and U373) as compared to NHA; **(B)** Effect of HA-treatment on moesin and CD44 expression was determined using Western blotting. No significant difference in expression of either **(i)** moesin, or **(ii)** CD44 was observed on treatment with HA in a dose dependent manner (50 - 100 μg/mL) for 24 - 48 h.

**Figure 4:**
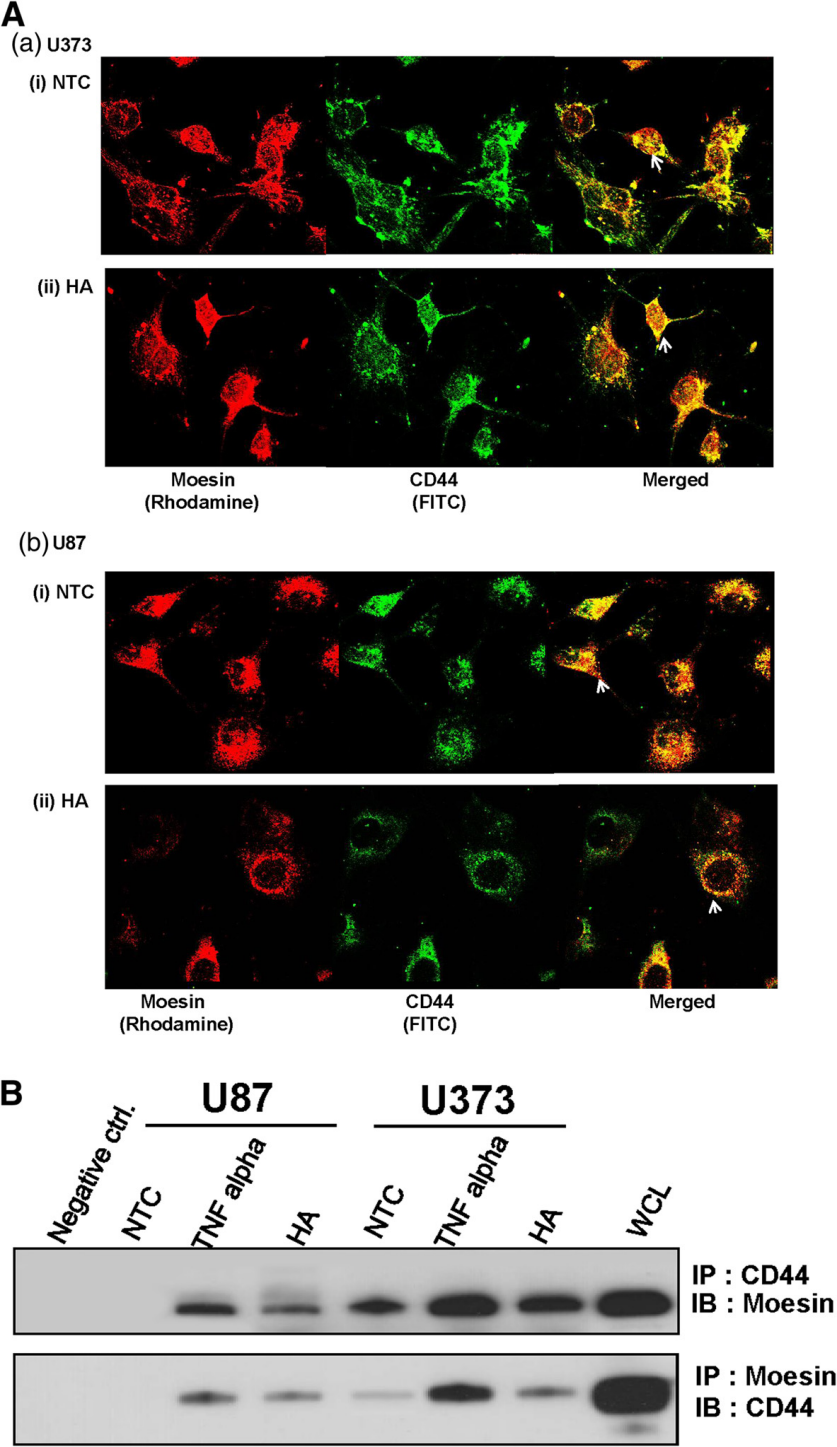
**HA-treatment induces CD44-moesin interaction. (A)** Both U373 and U87 cells were plated on coverslips and treated with HA (100 μg/mL, 24 h) followed by fixation and labeling with moesin and CD44 monoclonal antibodies as described in Materials and Methods. Panel shows images obtained using confocal laser scan microscopy (CLSM) representing localization of moesin (rhodamine, red) and CD44 (FITC, green) in glioma cell lines **(a)** U373 and **(b)** U87 in **(i)** no treatment controls (NTC) and **(ii)** HA-treated glioma cells. Arrows show membranous co-localization of moesin and CD44 in glioma cells (U373 / U87) (original magnification X600); **(B)** Co-IP assays for moesin / CD44 was performed using whole cell lysates obtained from HA (100 μg/mL, 48 h) or TNF-α (10 nM, 24 h) treated or no treatment control glioma cells (U373 / U87) using respective antibodies to determine moesin-CD44 interactions. Panel represents immunoblot (IB) of moesin showing a single band (~72 kDa, moesin) in CD44 immunoprecipitates (IP) obtained from HA-treated or TNF-α treated glioma cells (U87 / U373) and whole cell lysates (WCL of U373 cells) used as positive controls. No band of moesin was observed in negative controls, wherein pull-down was carried out using beads only or isotype mouse IgG. Similarly, moesin-immunoprecipitates showed a single band of CD44 in HA treated or TNF-α treated glioma cells (U87 / U373) and whole cell lysates (U373 cells, input used as a positive control).

To determine the expression levels of HA-receptor, CD44 and ERM protein, moesin, in NHA and glioma cell lines (U87 / U373), we carried out Western blot analysis. Our results demonstrated overexpression of both CD44 and moesin in both glioma cell lines as compared to NHA (Figure 3A). To determine the effect of HA-treatment on expression of CD44 and moesin, U373 / U87 cells were treated with HA at dose (50 – 100 μg/mL) for 24 – 48 h. Western blot analysis showed no significant change in expression of either moesin or CD44 in glioma cells (U87 / U373) on treatment with HA (Figure 3B). Notably, confocal laser scan microscopy revealed increase in membranous co-localization of both moesin and CD44 in HA-treated glioma cells in comparison to no treatment controls, suggesting HA-induced moesin-CD44 interaction in glioma cells may be responsible for increase in migratory cells (Figure 4A and 4B). This prompted us to further investigate the role of moesin in HA-induced cell migration in gliomas in the context of CD44-HA interaction.

### HA-treatment induces CD44-moesin interaction in glioma cells

To confirm the interaction of CD44 with moesin on HA-treatment, we performed co-IP assays with antibodies specific for CD44 / moesin using whole cell lysates obtained from glioma cells (U87 / U373) treated with either tumor necrosis factor-α (TNF-α, 10 nM for 24 h) or HA (100 μg/mL, 48 h), and no treatment control (NTC). Immunoprecipitation using CD44 antibody, followed by Western blot analysis with moesin antibody, revealed a single band (~72 kDa) that corresponded with the expected size for moesin in CD44 immunoprecipitates from both HA treated cell lines (Figure 4B). No such band was observed in untreated controls, suggesting that the interaction of moesin with CD44 was in response to HA treatment. Glioma cells treated with TNFα positive control) also showed a strong band for moesin (Figure 4B). No band corresponding to moesin was observed in negative controls, wherein immunoprecipitation was performed on lysates from HA-treated cells either without antibody or isotype mouse IgG (Figure 4B). These results were further confirmed using reverse immunoprecipitation assays. Moesin-immunoprecipitates obtained from both U87 and U373 cell lines treated either with HA (100 μg/ml, 48 h) or TNF α (10 nM, 24 h) and untreated controls. In all instances where the immunoprecipitates were obtained from HA- / TNF α treated cells, a band of CD44 was observed in lanes (Figure 4B). No band of CD44 was observed in immunoblots in lanes corresponding to the negative controls (Figure 4B). This clearly demonstrated an important role of moesin downstream of HA-CD44 interaction in glioma cells.

### Role of moesin in HA-induced cell migration in glioma cells

**Figure 5:**
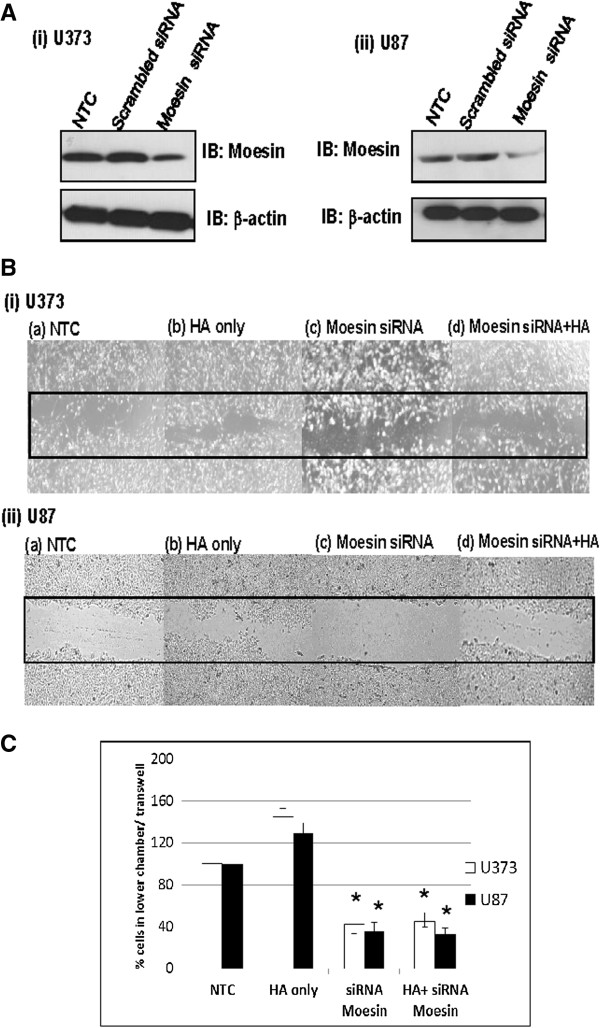
**Role of moesin in HA-induced migration in glioma cells. (A)** Western blot analysis. Both the glioma cell lines (U87 and U373) were transfected with siRNA targeting moesin and scrambled siRNA used as a negative control for 48 h followed by Western blotting. Panel represents shows effective downregulation (>70%) of moesin in both U87 and U373 cells in 48 h as compared with no transfection controls (NTC). Cells transfected with scrambled siRNA showed no significant change in the expression of moesin. β-actin was used as a loading control in western blot; **(B)** Glioma cells (U373 and U87) were transfected with moesin siRNA (200 nM) followed by treatment HA (100 μg/mL,48 h) and analyzed using wound healing assay as described in Materials and Methods. Panel shows number of cells migrating in the wound **(i)** no treatment controls (NTC); **(ii)** HA treatment (100 μg/mL, 48 h); **(iii)** cells transfected with moesin siRNA (200 nM, 48 h); (iv) cells transfected with moesin siRNA followed by treatment with HA (Original magnification X40). **(C)** Bar graph showing relative percentage of glioma cells (U373 / U87) that migrated into the lower chamber of transwell plates in no treatment controls (NTC), glioma cells treated with HA only, cells transfected with moesin siRNA (i.e. the cells with reduced moesin expression) and glioma cells transfected with siRNA targeting moesin followed by treatment with HA. As shown in the graph, glioma cells showing reduced expression of moesin demonstrated significantly reduced migration in transwell chambers even after treatment with HA (n = 3, *p-value < 0.01). This clearly demonstrates the significance of moesin downstream of HA-CD44 interaction in glioma cells.

U87 and U373 cells were transfected with either siRNA targeting moesin or negative control scrambled siRNA. Western blot analysis of the cell lysates showed downregulation (>70%) of moesin expression in both cell lines (U87 / U373) transfected with moesin siRNA in comparison to no treatment and negative control cells (Figure 5A). Notably, glioma cells showing lower expression of moesin also showed reduced cell migration in wound-healing assays as compared to no transfection controls (Figure 5B). Furthermore, to demonstrate the role of moesin in HA-induced cell migration, both glioma cell lines were transfected with moesin siRNA followed by treatment with HA (100 μg/mL) for 48 h. Wound-healing assay of these siRNA transfected and HA treated cells showed reduced cell migration relative to the no treatment control cells, demonstrating the significance of moesin in HA-induced cell migration in GBM cells (Figure 5B).

These results were further verified using transwell migration assays. Both glioma cell lines (U87 and U373) transfected with siRNA targeting moesin, resulting in its reduced expression, demonstrated significantly lower migration from the upper chamber to the lower chamber as compared to no transfection controls. Similar results were observed in glioma cells transfected with siRNA targeting moesin followed by HA-treatment, in comparison to no transfections controls and cells transfected with scrambled siRNA when treated with HA (Figure 5C).

## Discussion

In this study, we used multidimensional LC-MS/MS analysis for identification of differentially expressed proteins labeled with isobaric mass tags (iTRAQ) in clinical samples of GBM relative to non-malignant brain tissues. Our study led to the identification of 57 differentially expressed proteins of which 23 were upregulated and 34 downregulated in GBM tissues. Interestingly, stem cell markers (CD44 and nestin), ERM proteins (specifically moesin) and calcium binding proteins of the S100 family (S100A11 & S100A6) were upregulated in GBM tissues. Three of the seven isoforms of the 14-3-3 family, 14-3-3 zeta, gamma and epsilon, and metabolic enzymes such as gamma enolase were downregulated in GBM tissues as compared to non-malignant brain tissues.

Significantly, knockdown of moesin alone among ERM family of proteins reduced migration of GBM cells. Moesin belongs to the ERM (ezrin, radixin, moesin) family of proteins that functions as molecular cross-linkers between plasma membrane and cytoplasmic actin filaments [15,17,18]. ERM proteins are actively involved in actin cytoskeleton reorganization in response to several growth stimuli and serve as substrates for growth factor receptors including CD44, CD43, E-cadherin and intercellular adhesion molecule (ICAM-1, ICAM-2 and ICAM-3) [15]. ERM proteins are required for the formation of focal adhesion complexes and stress fibers downstream of Rho-GTPase activation. Knocking down the expression of ERM proteins using anti-sense oligonucleotides or siRNA affects cell-cell and cell-matrix adhesion [15,17,18]. Notably, ezrin and radixin, the other members of the ERM family, showed no significant differential expression in GBM and normal brain tissues as revealed by our iTRAQ analysis.

Overexpression of CD44, a transmembrane glycoprotein, has been reported in glioblastoma, breast, prostate, cervical, ovarian and head and neck cancer [19-23]. CD44 overexpression is responsible for increased survival, proliferation, migration, invasion and metastasis leading to poor prognosis among cancer patients [19-23]. Our study reveals overexpression of CD44 is also true in GBM and that it is localized to the membrane. CD44 serves as a major receptor for hyaluronan (HA), a polysaccharide that forms an important component of extracellular matrix (ECM) [15,16]. The levels of HA and its receptor CD44 in tumor cells are predictive of malignancy and often correlate with cancer aggressiveness in patients with glioblastoma, breast, prostate and non-small cell lung cancers [15,16,19-23].

HA-CD44 interaction potentially mediates the activation of ankyrin and Rho GTPase in ovarian and breast cancer cells [24,25]. CD44-EGFR interaction in glioblastoma cells enhanced HA-mediated phosphorylation of extracellular signal regulated kinases 1 and 2 (ERK1 and ERK2). HA treatment activates and promotes EGFR-mediated pathways including Ras, RhoA, Rho kinase, and phosphatidylinositol-3 (PI-3) kinase signaling in head and neck cancer [26-28]. The CD44-EGFR complex also associates with leukemia-associated Rho-guanine (LARG) nucleotide exchange factor activating downstream signaling through ras and RhoA [26-30]. Recent studies also revealed CD44 overexpression potentiates the migration and invasion of breast cancer cells and promotes metastasis to the liver through CD44–HA / NFκB / cortactin signaling pathway [31]. Our results also demonstrate increased migration in response to HA-treatment in glioma cells lines. However, the downstream targets underlying HA-CD44 interaction leading to increased migration and invasion in glioblastoma cells remain unidentified.

HA-induced CD44-moesin interaction resulted in increased cell migration, while knockdown of moesin reduced migration of GBM cells, even in cells treated with HA. This indicates that HA-induced CD44-moesin interaction contributes to increased migration in GBM cells. In support of our hypothesis, Hirao *et al*., [32] demonstrated that the cytoplasmic domain of mouse recombinant CD44 has a very weak binding activity to any of the ERM proteins at physiological ionic strength, but that the presence of phosphatidylinositol-4,5-bisphosphate (4,5-PIP2) causes it to bind ERM proteins with high affinity. This interaction results in an increase in the number of actin filament-association sites on the cytoplasmic surface of plasma membranes. This data supports our results demonstrating co-localization of CD44-moesin on advancing edges of plasma membrane in HA-treated glioblastoma cells. In addition, Rho-GDP dissociation inhibitor (GDI), a regulator of a small GTP binding protein Rho, also plays an important role in formation of the CD44 / moesin complex *in vivo*. The Rho-dependent enhancement of the CD44 / ERM complex formation provides an increasing number of actin filament attachment sites on the cytoplasmic surface of plasma membranes at microvilli, cleavage furrows, ruffling membranes, and cell-cell / cell-matrix adhesion sites [32].

## Conclusions

Our mass spectrometry-based analysis of GBM and non-malignant brain tissues yields insight into the differential expression of proteins involved in migration and invasion of glioblastoma cells. We show that the significant role of CD44-moesin interaction in cellular migration is in response to hyaluronan, an important component of the tumor microenvironment. Our results suggest that development of inhibitors which interfere with CD44-moesin interactions may provide a means to counteracting cellular migration in gliomas.

## Methods

### Tissue processing and iTRAQ labelling

Flash-frozen human GBM operative samples, non-malignant brain tissues (n = 6 each) and paraffin-embedded GBM tissues sections were obtained from the University of Toronto, Nervous System Tumor bank, in accordance with Research Ethics Board guidelines and consent. Six tissue samples, each of GBM and non-malignant brain (NB), were washed thrice in 1 mL of cold phosphate buffered saline (PBS, 0.1 M, pH = 7.2) and homogenized in 0.5 mL of PBS with a cocktail of protease inhibitors (1 mM 4-(2-aminoethyl) benzenesulfonyl fluoride, 10 *μ*M leupeptin, 1 μg/mL aprotinin and 1 μM pepstatin), centrifuged and the supernatant was collected. The total protein concentration was determined using a Bradford-type colorimetric assay as described earlier [7-11]. For each set, clarified sample lysates were individually denatured, alkylated, digested with trypsin and labeled with iTRAQ labels. The labeled samples were then pooled to form three 4-plex iTRAQ sets (see Additional file 1:Table S1). Each iTRAQ set was processed by offline two-dimensional LC-MS/MS analysis as described earlier [7-11].

### Mass spectrometry and data analysis

Tandem MS analysis was performed on a QSTAR Pulsar (Applied Biosystems Inc/ MDS Analytical) instrument in information dependent acquisition (IDA) mode using Analyst QS version 1.1 (Applied Biosystems Inc) as described [7-11]. Data from each set of fractions were collectively searched against a database downloaded from Uniprot (download date: 2nd June 2010) that contained 34950 protein sequences, using ProteinPilot version 4.0 (Applied Biosystems). Besides matching spectra with sequences, ProteinPilot also features a grouping function that minimizes the redundancy between the proteins reported. Proteins reported herein are, therefore, only those for which an unshared peptide or group of peptides were detected. To provide a measure of confidence in the proteins reported, the database against which the mass spectrometric data was searched included a decoy database generated by reversing the protein sequences in the original database and concatenating this reverse database to the original database. Any proteins matching the reverse sequence are obviously false, thereby providing a basis for calculation of the false discovery rate (FDR). This FDR calculation was described in Tang *et al*., [33].

Relative quantification of proteins, which was simultaneously performed by ProteinPilot, was based on the areas of the iTRAQ signature ion peaks. Only peptides not shared with other reported protein (or group) contribute to the overall ratios reported for the protein. The overall protein ratios reported is a weighted average of the ratios of the contributing unique peptides, where the weighting factor is determined by the % error of the individual peptide ratios. Further, the reported ratios were normalized using a factor, termed the *applied bias* that is calculated based on the assumption that the majority of the proteins being compared between the samples in a set are expressed at similar levels [34].

### Protein alignment

To minimize redundancy between proteins reported in the three individual iTRAQ sets and to ensure consistency of reported isoforms from one set to the next, the results of the three sets were aligned using an Excel based Protein Alignment Template (an early version of which was kindly provided by Dr. Sean Seymour, AB SCIEX) [35]. A master list of all the proteins identified in this study was first generated by performing a search on the combined data from all three iTRAQ sets and duplicate runs using ProteinPilot. The Protein summaries from the ProteinPilot results for the individual sets were then imported into the template and the proteins were collated using the master list as the reference. The complete list and the individual ratios for each protein in each set are shown in Additional file 2: Table S2.

### Western blotting

Equal amounts of whole cell lysates from GBM (n = 3) and non-malignant brain tissues (n = 2) as used in iTRAQ analysis were subjected to Western blotting [36]. Briefly, equal amounts of proteins (50 μg) obtained from GBM, non-malignant brain tissues and glioma cells were resolved on sodium dodecyl sulphate - polyacrylamide gels (SDS-PAGE). The proteins were then electro-transferred onto nitrocellulose membranes (BioRad, Hercules, CA). After blocking with 5% non-fat powdered milk in Tris-buffered saline (TBS, 0.1 M, pH = 7.4), blots were incubated with mouse monoclonal anti-moesin (cat no. ab3196) / anti-γ-enolase (cat no. sc-376375) / anti-β-actin (cat no. ab123020), rabbit monoclonal anti-CD44 (cat no. ab51037) / rabbit polyclonal 14-3-3ζ (cat no. sc1019) / anti-S100A11 (cat no. sc-98427) antibody at 4°C overnight. Membranes were incubated with secondary antibody, HRP-conjugated / rabbit / mouse anti-IgG (BioRad, CA), diluted at an appropriate dilution in 1% bovine serum albumin (BSA), for 2 h at room temperature. After each step, blots were washed three times with Tween (0.1%) -Tris-buffer saline (TTBS). Protein bands were detected by the enhanced chemiluminescence method (GE Health Care) on Kodak Hyperfilm.

### Immunohistochemistry of moesin and CD44 in GBM tissues

Immunohistochemistry for moesin and CD44 were carried out in independent set of paraffin embedded sections of GBM in tissue microarray format (TMA) as described earlier [37]. In brief, the sections were deparaffinized in xylene, hydrated in gradient alcohol, and pre-treated in a microwave oven for 15 min at maximum power in citrate buffer (0.01 M, pH = 6.0, 0.05% Tween-20) for antigen retrieval. The sections were incubated with hydrogen peroxide (0.3% v/v) in phosphate buffered saline (PBS, 0.1 M, pH = 7.2) for 15 min to quench the endogenous peroxidase activity, followed by blocking with 5% BSA to preclude non-specific binding. Thereafter, the slides were incubated with mouse monoclonal anti-moesin antibody / rabbit monoclonal anti-CD44 for 16 h at 4°C. The primary antibody was detected using the streptavidin-biotin complex with the Dako LSAB plus kit (Dako Cytomation, Glostrup, Denmark) and diaminobenzidine as chromogen [10,13,37]. All procedures were carried out at room temperature unless otherwise specified. Slides were washed three times using PBS containing 0.025% Triton-X-100, after every step. Finally, the sections were counterstained with Mayer’s hematoxylin and mounted with DPX mountant. In the negative control tissue sections, the primary antibody was replaced by isotype specific non-immune mouse IgG. The sections were evaluated by light microscopic examination.

### Cell culture, treatment with HA and siRNA transfections

GBM cells (U87 and U373) and normal human astrocytes (NHA) immortalized with human telomerase construct (hTERT) were a kind gift from Dr. Abhijit Guha, The Hospital for Sick Children, University of Toronto, Toronto, Ontario. Cells were grown in monolayer cultures in Dulbecco’s modified eagle medium (DMEM) (Sigma, St. Louis, MO) supplemented with 10% FBS, 1 mM L-glutamine, 1 mM minimum essential medium (MEM), 100 μg/mL streptomycin and 100 U/mL penicillin in a humidified incubator (5% carbon-dioxide, 95% air) at 37°C as described earlier [11,36]. Both the GBM cell lines and NHA cells were treated with HA (Sigma St. Louis, MO) at dose range (50 - 200 μg/mL) for 6 - 48 h. For all experiments, HA was suspended in the growth medium, i.e. DMEM only without FBS. Effect of treatment on cell migration, proliferation, cell cycle and expression of moesin, CD44 was determined as described below. Pre-validated siRNA targeting moesin was obtained from Ambion (Life Technologies, CA). Negative control siRNA showing no homology to any of the known mammalian gene (i.e., having a scrambled sequence) and Cy3-labeled control siRNA for determining transfection efficiency were also obtained from Ambion (Life Technologies, CA). Transient transfection of siRNAs was performed using Lipofectamine 2000 and cells were collected after 48 h post-transfection for further Western blot analyses as described earlier [11,36].

### Wound-healing and cell migration assays

For wound-healing assays, 1×10^6^ GBM cells (U87 / U373) or NHA cells were plated in 6-well plates for 24 h. Cells were either left untreated (no treatment controls), treated with HA (50 - 100 μg/mL) suspended in DMEM only or transfected with siRNA targeting moesin (200 nM, 24 - 72 h). Further, to determine the role of moesin downstream of HA - CD44 induced cell migration, glioma cells were transfected with moesin siRNA followed by treatment HA (100 μg/mL) for another 48 h. Similar-sized wounds were created in monolayer cells by scraping a gap using a micropipette tip either before treatment with HA or 24 h after siRNA transfections. After removing cell debris by rinsing with phosphate-buffered saline, fresh medium was added, and the cells started migrating from the edge of the wound and repopulated the gap area. Cells were then observed for ‘wound closure’ under a light microscope after 6 – 48 h and the number of cells were counted in the wound for quantitative analysis among treated cells and no treatment controls.

Further these results were verified by carrying out migration assays performed using gelatin-coated polycarbonate filters (pore size, 8 μm) on transwells separating the upper and lower chamber of 24-well plates (BD BioSciences). Chemotaxis medium (DMEM with 0.5% BSA and 10 mM HEPES) was added to the bottom chamber. siRNA transfected, HA-treated or no transfection control cells (1 × 10^5^ / well) suspended in DMEM containing either HA (100 μg/mL) or DMEM only used as no treatment controls (NTC) were placed in the upper chamber. The plates were incubated at 37°C overnight. Viable cells in the lower chamber were collected and counted [11].

### Cell proliferation assay

Glioma cells (U87 / U373) were plated in triplicates in 96 - well plates in complete medium (i.e. DMEM containing 10% FBS). The cells were cultured overnight and then either treated with HA (50 – 200 μg/mL) in DMEM only, for 24 - 48 h to determine dose- and time-dependent effect on cell proliferation. Cell proliferation was measured by adding MTT at 37°C for 3 - 4 h. The formazan crystals were dissolved in 100 μl of dimethylsulphoxide (DMSO) and the optical density (OD) was measured at a wavelength of 570 nm as described earlier [36].

### Cell-cycle analysis using flow cytometry

HA-treated (100 μg/mL) or untreated no treatment control glioma cells (U87 / U373) were collected and centrifuged to collect non-adherent cells. Adherent cells were washed with phosphate buffered saline (PBS, pH = 7.4) and trypsinized. Both non-adherent and adherent cell populations were pooled for further analysis. Cells were fixed in 70% ethanol and resuspended in buffer containing PBS (pH = 7.4), EDTA (0.5 M, pH = 8.0), Triton X-100 (0.05%), RNAse A (50 μg/ml) and propidium iodide (PI, 100 μg/ml) before flow cytometry analysis. The PI-labeled cells were analyzed using a BD Canto flow cytometer and the output thus obtained was analyzed using the BD Cell Quest Pro software as described earlier [36]. Cells were gated to exclude cell debris and cell clumps.

### Co-immunoprecipitation (co-IP) assays

Co-immunoprecipitation assays to determine CD44 - moesin interactions were carried out using specific antibodies (CD44 / moesin) and analyzed by Western blotting as described earlier [37]. Briefly, GBM cells (U87 / U373) treated with either HA (100 μg/mL, 48 h) or (TNF-α, 10 nM, 24 h) and no treatment control cells were rinsed in ice-cold PBS and lysed in IP lysis buffer [38]. Lysates were incubated on ice for 30 min. and cell debris was removed by centrifugation. Lysates were pre-cleared by adding 20 μL of Protein A agarose (Santa Cruz Biotech., Santa Cruz, CA), followed by overnight incubation with specific antibodies (CD44 / moesin) on a rocker at 4°C. Immunocomplexes were pulled down by incubating with Protein A-agarose for 2 h at 4°C, followed by washing with 1X ice-cold lysis buffer to eliminate non-specific interactions. Protein A-agrose-bound immunocomplexes were then resuspended in Laemelli sample buffer (1X), boiled for 5 min. and analyzed by Western blotting. In addition, glioma cells treated with tumor necrosis factor-α (TNF-α) were used as a positive control for co-IP experiments [37]. In negative controls, primary antibodies used for co-IP was replaced by isotype IgG controls [37].

### Statistical analysis

Each experiment was repeated at least twice or was performed in triplicates. Significance of the difference between two measurements was determined using Students *t*-test; p-value < 0.05 was considered as significant [11,36]. Numerical data are represented as the mean ± standard deviation.

## Abbreviations

4,5-PIP2: Phosphatidylinositol 4,5-bisphosphate; CLSM: Confocal laser scan microscopy; co-IP: Co-immunoprecipitation; DMEM: Dulbecco’s modified eagle medium; ECM: Extracellular matrix; EGFR: Epidermal growth factor receptor; ERM: Ezrin, radixin, moesin; FDR: False discovery rate; GBM: Glioblastoma multiforme; HA: Hyaluronan; IHC: Immunohistochemistry; IDA: Information dependent acquisition; LC-MS/MS: Liquid chromatography-tandem mass spectrometry; LARG: Leukemia-associated Rho-guanine; NB: Non -malignant brain; NHA: Normal human astrocytes; PI-3 K: Phosphatidylinositol-3 kinase; TNF-α: Tumor necrosis factor-α.

## Competing interests

All authors declare no conflict of interest.

## Authors’ contributions

LVD and AM conceptualized and performed experiments and wrote the manuscript. ZK and SW performed some of the experiments. JM and GZ provided the tissue samples used in the manuscript and helped in experimental design. OK performed the statistical analysis. AG provided tissue samples and cell lines used for the experiments and experimental design. KWMS conceptualized experiments, edited manuscript, provided funding and resources required for conducting all experiments. All authors read and approved the final manuscript.

## Supplementary Material

Additional file 1 Experimental design for iTRAQ labeling of tissue samples. Click here for file

Additional file 2 Complete results showing protein identifications and ratios. Click here for file

Additional file 3: Figure S1 Effect of HA-treatment on cell migration. Glioma cells (U373 and U87) were plated and treated with HA (100 μg/mL) or TNF-α (10 nM) for 6 - 24 h. All HA- / TNF-α treatments were given in DMEM only. Panel **(A)** shows no significant reduction in wound or number of cells in wound in either of the glioma cell lines **(i)** U373 and **(ii)** U87 when treated with HA (100 μg/mL) for 6 – 24 h as compared to no treatment controls (NTC); Panel shows data for **(i)** NTC; **(ii)**12 h and **(iii)** 24 h treatment; Panel **(B)** shows a significant reduction in wound and increase in num ber of cells in the wound of U87 cells treated with TNF-α (10 nM) as early as 12 h. Treatment with TNF-α (10 nM) served as positive control for wound healing assays (Original magnification X40). Click here for file

Additional file 4: Figure S2 **(A)** Cell viability assay. To determine the effect of HA-treatment on cell proliferation, GBM cells (U87 / U373) were plated in triplicates in 96-well plates in complete medium followed by treatment with varying concentrations of hyaluronan (HA) in DMEM only for 24 - 48 h. Panel **A** shows no significant difference in cell proliferation in glioma cells (U373 & U87) on treatment with HA (50 - 200 μg/mL) for 24 - 48 h; **(B)** Cell Cycle Analysis. For cell cycle analysis, both HA-treated and untreated no treatment control cells were collected, fixed and suspended in FACS buffer as described in Materials And Methods section. Panel **B** shows no significant difference in cell cycle in glioma cells (U373 & U87) on treatment with HA (100 μg/mL) for 48 h. Click here for file
