# Supplementary material for: Role of moesin in hyaluronan induced cell migration in glioblastoma multiforme
Source: Mol Cancer. 2013 Jul 15;12:74. doi: 10.1186/1476-4598-12-74 (PMC3718631; doi:10.1186/1476-4598-12-74)
Supplement: Additional file 1 — Experimental design for iTRAQ labeling of tissue samples. [file 1476-4598-12-74-S1.pdf]

Supplementary Table S1

iTRAQ Set	114	115	116	117
1	NB1	GBM1	NB2 (Ref sample)	GBM2
2	GBM4	NB3 (Ref sample)	GBM3	NB4
3	NB5	GBM5	NB6 (Ref sample)	GBM6
