# Supplementary material for: Role of moesin in hyaluronan induced cell migration in glioblastoma multiforme
Source: Mol Cancer. 2013 Jul 15;12:74. doi: 10.1186/1476-4598-12-74 (PMC3718631; doi:10.1186/1476-4598-12-74)
Supplement: Additional file 2 — Complete results showing protein identifications and ratios. [file 1476-4598-12-74-S2.pdf]

Table S2: Complete results showing protein identifications and ratios.

Master N	Accession	Name
1	sp|P02768|ALBU_HUMAN	Serum albumin OS=Homo sapiens GN=ALB PE=1 SV=2
2	sp|P69905|HBA_HUMAN	Hemoglobin subunit alpha OS=Homo sapiens GN=HBA1 PE=1 SV=2
3	sp|P68871|HBB_HUMAN	Hemoglobin subunit beta OS=Homo sapiens GN=HBB PE=1 SV=2
4	sp|P08670|VIME_HUMAN	Vimentin OS=Homo sapiens GN=VIM PE=1 SV=4
5	sp|Q13813-2|SPTA2_HUMAN	Isoform 2 of Spectrin alpha chain, brain OS=Homo sapiens GN=SPTAN1
6	sp|P14136|GFAP_HUMAN	Glial fibrillary acidic protein OS=Homo sapiens GN=GFAP PE=1 SV=1
7	sp|P01024|CO3_HUMAN	Complement C3 OS=Homo sapiens GN=C3 PE=1 SV=2
8	sp|P21333-2|FLNA_HUMAN	Isoform 2 of Filamin-A OS=Homo sapiens GN=FLNA
9	sp|Q01082|SPTB2_HUMAN	Spectrin beta chain, brain 1 OS=Homo sapiens GN=SPTBN1 PE=1 SV=2
10	sp|P02787|TRFE_HUMAN	Serotransferrin OS=Homo sapiens GN=TF PE=1 SV=2
11	sp|P02686|MBP_HUMAN	Myelin basic protein OS=Homo sapiens GN=MBP PE=1 SV=3
12	sp|P07900|HS90A_HUMAN	Heat shock protein HSP 90-alpha OS=Homo sapiens GN=HSP90AA1 PE=1 SV=5
13	sp|P60709|ACTB_HUMAN	Actin, cytoplasmic 1 OS=Homo sapiens GN=ACTB PE=1 SV=1
14	sp|Q16555|DPYL2_HUMAN	Dihydropyrimidinase-related protein 2 OS=Homo sapiens GN=DPYSL2 PE=1 SV=1
15	sp|P04406|G3P_HUMAN	Glyceraldehyde-3-phosphate dehydrogenase OS=Homo sapiens GN=GAPDH PE=1 SV=3
16	sp|P11142|HSP7C_HUMAN	Heat shock cognate 71 kDa protein OS=Homo sapiens GN=HSPA8 PE=1 SV=1
17	sp|P11137-3|MAP2_HUMAN	Isoform 3 of Microtubule-associated protein 2 OS=Homo sapiens GN=MAP2
18	sp|P14618-2|KPYM_HUMAN	Isoform M1 of Pyruvate kinase isozymes M1/M2 OS=Homo sapiens GN=PKM2
19	sp|P01023|A2MG_HUMAN	Alpha-2-macroglobulin OS=Homo sapiens GN=A2M PE=1 SV=2
20	sp|P00558|PGK1_HUMAN	Phosphoglycerate kinase 1 OS=Homo sapiens GN=PGK1 PE=1 SV=3
21	sp|P68363|TBA1B_HUMAN	Tubulin alpha-1B chain OS=Homo sapiens GN=TUBA1B PE=1 SV=1
22	sp|P01009|A1AT_HUMAN	Alpha-1-antitrypsin OS=Homo sapiens GN=SERPINA1 PE=1 SV=3
23	sp|P06733|ENOA_HUMAN	Alpha-enolase OS=Homo sapiens GN=ENO1 PE=1 SV=2
24	sp|P04075|ALDOA_HUMAN	Fructose-bisphosphate aldolase A OS=Homo sapiens GN=ALDOA PE=1 SV=2
25	sp|P12814|ACTN1_HUMAN	Alpha-actinin-1 OS=Homo sapiens GN=ACTN1 PE=1 SV=2
26	sp|Q13885|TBB2A_HUMAN	Tubulin beta-2A chain OS=Homo sapiens GN=TUBB2A PE=1 SV=1
27	sp|P00367|DHE3_HUMAN	Glutamate dehydrogenase 1, mitochondrial OS=Homo sapiens GN=GLUD1 PE=1 SV=2
28	sp|P46821|MAP1B_HUMAN	Microtubule-associated protein 1B OS=Homo sapiens GN=MAP1B PE=1 SV=1
29	sp|P26038|MOES_HUMAN	Moesin OS=Homo sapiens GN=MSN PE=1 SV=3
30	sp|P00738|HPT_HUMAN	Haptoglobin OS=Homo sapiens GN=HP PE=1 SV=1
31	sp|P80723|BASP1_HUMAN	Brain acid soluble protein 1 OS=Homo sapiens GN=BASP1 PE=1 SV=2
32	sp|P09543|CN37_HUMAN	2',3'-cyclic-nucleotide 3'-phosphodiesterase OS=Homo sapiens GN=CNP PE=1 SV=2
33	sp|Q99798|ACON_HUMAN	Aconitate hydratase, mitochondrial OS=Homo sapiens GN=ACO2 PE=1 SV=2
34	sp|P12277|KCRB_HUMAN	Creatine kinase B-type OS=Homo sapiens GN=CKB PE=1 SV=1
35	sp|P10636-7|TAU_HUMAN	Isoform Tau-E of Microtubule-associated protein tau OS=Homo sapiens GN=MAPT
36	sp|P17600-2|SYN1_HUMAN	Isoform IB of Synapsin-1 OS=Homo sapiens GN=SYN1
37	sp|P10809|CH60_HUMAN	60 kDa heat shock protein, mitochondrial OS=Homo sapiens GN=HSPD1 PE=1 SV=2
38	sp|P17677|NEUM_HUMAN	Neuromodulin OS=Homo sapiens GN=GAP43 PE=1 SV=1
39	sp|P01857|IGHG1_HUMAN	Ig gamma-1 chain C region OS=Homo sapiens GN=IGHG1 PE=1 SV=1
40	sp|P02647|APOA1_HUMAN	Apolipoprotein A-I OS=Homo sapiens GN=APOA1 PE=1 SV=1
41	sp|Q00610-2|CLH1_HUMAN	Isoform 2 of Clathrin heavy chain 1 OS=Homo sapiens GN=CLTC
42	sp|P00915|CAH1_HUMAN	Carbonic anhydrase 1 OS=Homo sapiens GN=CA1 PE=1 SV=2
43	sp|P14625|ENPL_HUMAN	Endoplasmin OS=Homo sapiens GN=HSP90B1 PE=1 SV=1
44	sp|P06396|GELS_HUMAN	Gelsolin OS=Homo sapiens GN=GSN PE=1 SV=1
45	sp|P29966|MARCS_HUMAN	Myristoylated alanine-rich C-kinase substrate OS=Homo sapiens GN=MARCKS PE=1 SV=4
46	sp|P06576|ATPB_HUMAN	ATP synthase subunit beta, mitochondrial OS=Homo sapiens GN=ATP5B PE=1 SV=3

**Master N**	**Accession**	**Name**
47	sp|P09972|ALDOC_HUMAN	Fructose-bisphosphate aldolase C OS=Homo sapiens GN=ALDOC PE=1 SV=2
48	sp|P25705|ATPA_HUMAN	ATP synthase subunit alpha, mitochondrial OS=Homo sapiens GN=ATP5A1 PE=1 SV=1
49	sp|P04083|ANXA1_HUMAN	Annexin A1 OS=Homo sapiens GN=ANXA1 PE=1 SV=2
50	sp|P60174|TPIS_HUMAN	Triosephosphate isomerase OS=Homo sapiens GN=TPI1 PE=1 SV=2
51	sp|P48681|NEST_HUMAN	Nestin OS=Homo sapiens GN=NES PE=1 SV=2
52	sp|P68104|EF1A1_HUMAN	Elongation factor 1-alpha 1 OS=Homo sapiens GN=EEF1A1 PE=1 SV=1
53	sp|P40926|MDHM_HUMAN	Malate dehydrogenase, mitochondrial OS=Homo sapiens GN=MDH2 PE=1 SV=3
54	sp|P04040|CATA_HUMAN	Catalase OS=Homo sapiens GN=CAT PE=1 SV=3
55	sp|P62258|1433E_HUMAN	14-3-3 protein epsilon OS=Homo sapiens GN=YWHAE PE=1 SV=1
56	sp|P06744|G6PI_HUMAN	Glucose-6-phosphate isomerase OS=Homo sapiens GN=GPI PE=1 SV=4
57	sp|P29401|TKT_HUMAN	Transketolase OS=Homo sapiens GN=TKT PE=1 SV=3
58	sp|P31150|GDIA_HUMAN	Rab GDP dissociation inhibitor alpha OS=Homo sapiens GN=GDI1 PE=1 SV=2
59	sp|P46459|NSF_HUMAN	Vesicle-fusing ATPase OS=Homo sapiens GN=NSF PE=1 SV=3
60	sp|P06753-2|TPM3_HUMAN	Isoform TM30nm of Tropomyosin alpha-3 chain OS=Homo sapiens GN=TPM3
61	sp|P40925|MDHC_HUMAN	Malate dehydrogenase, cytoplasmic OS=Homo sapiens GN=MDH1 PE=1 SV=4
62	sp|P11021|GRP78_HUMAN	78 kDa glucose-regulated protein OS=Homo sapiens GN=HSPA5 PE=1 SV=2
63	sp|P07195|LDHB_HUMAN	L-lactate dehydrogenase B chain OS=Homo sapiens GN=LDHB PE=1 SV=2
64	sp|P08238|HS90B_HUMAN	Heat shock protein HSP 90-beta OS=Homo sapiens GN=HSP90AB1 PE=1 SV=4
65	sp|P08107|HSP71_HUMAN	Heat shock 70 kDa protein 1A/1B OS=Homo sapiens GN=HSPA1A PE=1 SV=5
66	sp|P35579|MYH9_HUMAN	Myosin-9 OS=Homo sapiens GN=MYH9 PE=1 SV=4
67	sp|P32119|PRDX2_HUMAN	Peroxiredoxin-2 OS=Homo sapiens GN=PRDX2 PE=1 SV=5
68	sp|P01011|AACT_HUMAN	Alpha-1-antichymotrypsin OS=Homo sapiens GN=SERPINA3 PE=1 SV=2
69	sp|P02511|CRYAB_HUMAN	Alpha-crystallin B chain OS=Homo sapiens GN=CRYAB PE=1 SV=2
70	sp|P27816-6|MAP4_HUMAN	Isoform 6 of Microtubule-associated protein 4 OS=Homo sapiens GN=MAP4
71	sp|P07355|ANXA2_HUMAN	Annexin A2 OS=Homo sapiens GN=ANXA2 PE=1 SV=2
72	sp|P27797|CALR_HUMAN	Calreticulin OS=Homo sapiens GN=CALR PE=1 SV=1
73	sp|P61764-2|STXB1_HUMAN	Isoform HUNC18b of Syntaxin-binding protein 1 OS=Homo sapiens GN=STXBP1
74	sp|P09936|UCHL1_HUMAN	Ubiquitin carboxyl-terminal hydrolase isozyme L1 OS=Homo sapiens GN=UCHL1 PE=1 SV=2
75	sp|P00338|LDHA_HUMAN	L-lactate dehydrogenase A chain OS=Homo sapiens GN=LDHA PE=1 SV=2
76	sp|P07197|NFM_HUMAN	Neurofilament medium polypeptide OS=Homo sapiens GN=NEFM PE=1 SV=3
77	sp|O43707|ACTN4_HUMAN	Alpha-actinin-4 OS=Homo sapiens GN=ACTN4 PE=1 SV=2
78	sp|Q14195|DPYL3_HUMAN	Dihydropyrimidinase-related protein 3 OS=Homo sapiens GN=DPYSL3 PE=1 SV=1
79	sp|P23528|COF1_HUMAN	Cofilin-1 OS=Homo sapiens GN=CFL1 PE=1 SV=3
80	sp|P55072|TERA_HUMAN	Transitional endoplasmic reticulum ATPase OS=Homo sapiens GN=VCP PE=1 SV=4
81	sp|P08758|ANXA5_HUMAN	Annexin A5 OS=Homo sapiens GN=ANXA5 PE=1 SV=2
82	sp|P30041|PRDX6_HUMAN	Peroxiredoxin-6 OS=Homo sapiens GN=PRDX6 PE=1 SV=3
83	sp|P07237|PDIA1_HUMAN	Protein disulfide-isomerase OS=Homo sapiens GN=P4HB PE=1 SV=3
84	sp|P22314|UBA1_HUMAN	Ubiquitin-like modifier-activating enzyme 1 OS=Homo sapiens GN=UBA1 PE=1 SV=3
85	sp|P63104|1433Z_HUMAN	14-3-3 protein zeta/delta OS=Homo sapiens GN=YWHAZ PE=1 SV=1
86	sp|P30101|PDIA3_HUMAN	Protein disulfide-isomerase A3 OS=Homo sapiens GN=PDIA3 PE=1 SV=4
87	sp|P02788|TRFL_HUMAN	Lactotransferrin OS=Homo sapiens GN=LTF PE=1 SV=6
88	sp|P02671-2|FIBA_HUMAN	Isoform Alpha of Fibrinogen alpha chain OS=Homo sapiens GN=FGA
89	sp|P08133|ANXA6_HUMAN	Annexin A6 OS=Homo sapiens GN=ANXA6 PE=1 SV=3
90	sp|P09104|ENOG_HUMAN	Gamma-enolase OS=Homo sapiens GN=ENO2 PE=1 SV=3
91	sp|P13639|EF2_HUMAN	Elongation factor 2 OS=Homo sapiens GN=EEF2 PE=1 SV=4
92	sp|Q06830|PRDX1_HUMAN	Peroxiredoxin-1 OS=Homo sapiens GN=PRDX1 PE=1 SV=1
93	sp|P38646|GRP75_HUMAN	Stress-70 protein, mitochondrial OS=Homo sapiens GN=HSPA9 PE=1 SV=2
94	sp|P00505|AATM_HUMAN	Aspartate aminotransferase, mitochondrial OS=Homo sapiens GN=GOT2 PE=1 SV=2

Master N	Accession	Name
95	sp|P62937|PPIA_HUMAN	Peptidyl-prolyl cis-trans isomerase A OS=Homo sapiens GN=PPIA PE=1 SV=2
96	sp|P22626|ROA2_HUMAN	Heterogeneous nuclear ribonucleoproteins A2/B1 OS=Homo sapiens GN=HNRNPA2B1 PE=1 S
97	sp|P01876|IGHA1_HUMAN	Ig alpha-1 chain C region OS=Homo sapiens GN=IGHA1 PE=1 SV=2
98	sp|P19367-4|HXK1_HUMAN	Isoform TD of Hexokinase-1 OS=Homo sapiens GN=HK1
99	sp|Q16181-2|SEPT7_HUMAN	Isoform 2 of Septin-7 OS=Homo sapiens GN=SEPT7
100	sp|P18669|PGAM1_HUMAN	Phosphoglycerate mutase 1 OS=Homo sapiens GN=PGAM1 PE=1 SV=2
101	sp|P48735|IDHP_HUMAN	Isocitrate dehydrogenase [NADP], mitochondrial OS=Homo sapiens GN=IDH2 PE=1 SV=2
102	sp|P16152|CBR1_HUMAN	Carbonyl reductase [NADPH] 1 OS=Homo sapiens GN=CBR1 PE=1 SV=3
103	sp|Q9Y2J2|E41L3_HUMAN	Band 4.1-like protein 3 OS=Homo sapiens GN=EPB41L3 PE=1 SV=2
104	sp|Q15084|PDIA6_HUMAN	Protein disulfide-isomerase A6 OS=Homo sapiens GN=PDIA6 PE=1 SV=1
105	sp|P00450|CERU_HUMAN	Ceruloplasmin OS=Homo sapiens GN=CP PE=1 SV=1
106	sp|P16949|STMN1_HUMAN	Stathmin OS=Homo sapiens GN=STMN1 PE=1 SV=3
107	sp|P61604|CH10_HUMAN	10 kDa heat shock protein, mitochondrial OS=Homo sapiens GN=HSPE1 PE=1 SV=2
108	sp|P00918|CAH2_HUMAN	Carbonic anhydrase 2 OS=Homo sapiens GN=CA2 PE=1 SV=2
109	sp|Q96KP4|CNDP2_HUMAN	Cytosolic non-specific dipeptidase OS=Homo sapiens GN=CNDP2 PE=1 SV=2
110	sp|P43490|NAMPT_HUMAN	Nicotinamide phosphoribosyltransferase OS=Homo sapiens GN=NAMPT PE=1 SV=1
111	sp|P13667|PDIA4_HUMAN	Protein disulfide-isomerase A4 OS=Homo sapiens GN=PDIA4 PE=1 SV=2
112	sp|P62158|CALM_HUMAN	Calmodulin OS=Homo sapiens GN=CALM1 PE=1 SV=2
113	sp|P04179|SODM_HUMAN	Superoxide dismutase [Mn], mitochondrial OS=Homo sapiens GN=SOD2 PE=1 SV=2
114	sp|P30086|PEBP1_HUMAN	Phosphatidylethanolamine-binding protein 1 OS=Homo sapiens GN=PEBP1 PE=1 SV=3
115	sp|Q9BY11|PACN1_HUMAN	Protein kinase C and casein kinase substrate in neurons protein 1 OS=Homo sapiens GN=PAC
116	sp|P09211|GSTP1_HUMAN	Glutathione S-transferase P OS=Homo sapiens GN=GSTP1 PE=1 SV=2
117	sp|O94760|DDAH1_HUMAN	N(G),N(G)-dimethylarginine dimethylaminohydrolase 1 OS=Homo sapiens GN=DDAH1 PE=1 S
118	sp|P15104|GLNA_HUMAN	Glutamine synthetase OS=Homo sapiens GN=GLUL PE=1 SV=4
119	sp|P35611|ADDA_HUMAN	Alpha-adducin OS=Homo sapiens GN=ADD1 PE=1 SV=2
120	sp|P23284|PPIB_HUMAN	Peptidyl-prolyl cis-trans isomerase B OS=Homo sapiens GN=PPIB PE=1 SV=2
121	sp|P61981|1433G_HUMAN	14-3-3 protein gamma OS=Homo sapiens GN=YWHAG PE=1 SV=2
122	sp|Q99497|PARK7_HUMAN	Protein DJ-1 OS=Homo sapiens GN=PARK7 PE=1 SV=2
123	sp|P01871|IGHM_HUMAN	Ig mu chain C region OS=Homo sapiens GN=IGHM PE=1 SV=3
124	sp|O75390|CISY_HUMAN	Citrate synthase, mitochondrial OS=Homo sapiens GN=CS PE=1 SV=2
125	sp|Q9NVA2|SEP11_HUMAN	Septin-11 OS=Homo sapiens GN=SEPT11 PE=1 SV=3
126	sp|P08237|K6PF_HUMAN	6-phosphofructokinase, muscle type OS=Homo sapiens GN=PFKM PE=1 SV=2
127	sp|P21281|VATB2_HUMAN	V-type proton ATPase subunit B, brain isoform OS=Homo sapiens GN=ATP6V1B2 PE=1 SV=3
128	sp|P04350|TBB4_HUMAN	Tubulin beta-4 chain OS=Homo sapiens GN=TUBB4 PE=1 SV=2
129	sp|P01859|IGHG2_HUMAN	Ig gamma-2 chain C region OS=Homo sapiens GN=IGHG2 PE=1 SV=2
130	sp|P69891|HBG1_HUMAN	Hemoglobin subunit gamma-1 OS=Homo sapiens GN=HBG1 PE=1 SV=2
131	sp|P05023|AT1A1_HUMAN	Sodium/potassium-transporting ATPase subunit alpha-1 OS=Homo sapiens GN=ATP1A1 PE=1
132	sp|P62328|TYB4_HUMAN	Thymosin beta-4 OS=Homo sapiens GN=TMSB4X PE=1 SV=2
133	sp|P78559|MAP1A_HUMAN	Microtubule-associated protein 1A OS=Homo sapiens GN=MAP1A PE=1 SV=5
134	sp|Q15149-7|PLEC1_HUMAN	Isoform Plectin-7 of Plectin-1 OS=Homo sapiens GN=PLEC1
135	sp|Q9UQM7|KCC2A_HUMAN	Calcium/calmodulin-dependent protein kinase type II subunit alpha OS=Homo sapiens GN=CAM
136	sp|P02790|HEMO_HUMAN	Hemopexin OS=Homo sapiens GN=HPX PE=1 SV=2
137	sp|Q05193-5|DYN1_HUMAN	Isoform 5 of Dynamin-1 OS=Homo sapiens GN=DNM1
138	sp|P28482|MK01_HUMAN	Mitogen-activated protein kinase 1 OS=Homo sapiens GN=MAPK1 PE=1 SV=3
139	sp|P17174|AATC_HUMAN	Aspartate aminotransferase, cytoplasmic OS=Homo sapiens GN=GOT1 PE=1 SV=3
140	sp|P38606|VATA_HUMAN	V-type proton ATPase catalytic subunit A OS=Homo sapiens GN=ATP6V1A PE=1 SV=2
141	sp|P02763|A1AG1_HUMAN	Alpha-1-acid glycoprotein 1 OS=Homo sapiens GN=ORM1 PE=1 SV=1
142	sp|P07737|PROF1_HUMAN	Profilin-1 OS=Homo sapiens GN=PFN1 PE=1 SV=2

Master N	Accession	Name
143	sp|P09417|DHPR_HUMAN	Dihydropteridine reductase OS=Homo sapiens GN=QDPR PE=1 SV=2
144	sp|O94811|TPPP_HUMAN	Tubulin polymerization-promoting protein OS=Homo sapiens GN=TPPP PE=1 SV=1
145	sp|P02774|VTDB_HUMAN	Vitamin D-binding protein OS=Homo sapiens GN=GC PE=1 SV=1
146	sp|P09471-2|GNAO_HUMAN	Isoform Alpha-2 of Guanine nucleotide-binding protein G(o) subunit alpha OS=Homo sapiens G
147	sp|P31948|STIP1_HUMAN	Stress-induced-phosphoprotein 1 OS=Homo sapiens GN=STIP1 PE=1 SV=1
148	sp|P49411|EFTU_HUMAN	Elongation factor Tu, mitochondrial OS=Homo sapiens GN=TUFM PE=1 SV=2
149	sp|P07108|ACBP_HUMAN	Acyl-CoA-binding protein OS=Homo sapiens GN=DBI PE=1 SV=2
150	sp|P26641|EF1G_HUMAN	Elongation factor 1-gamma OS=Homo sapiens GN=EEF1G PE=1 SV=3
151	sp|Q02252|MMSA_HUMAN	Methylmalonate-semialdehyde dehydrogenase [acylating], mitochondrial OS=Homo sapiens GN
152	sp|P00734|THRB_HUMAN	Prothrombin OS=Homo sapiens GN=F2 PE=1 SV=2
153	sp|P00441|SODC_HUMAN	Superoxide dismutase [Cu-Zn] OS=Homo sapiens GN=SOD1 PE=1 SV=2
154	sp|Q15019-2|SEPT2_HUMAN	Isoform 2 of Septin-2 OS=Homo sapiens GN=SEPT2
155	sp|P11216|PYGB_HUMAN	Glycogen phosphorylase, brain form OS=Homo sapiens GN=PYGB PE=1 SV=5
156	sp|P61160|ARP2_HUMAN	Actin-related protein 2 OS=Homo sapiens GN=ACTR2 PE=1 SV=1
157	sp|P09382|LEG1_HUMAN	Galectin-1 OS=Homo sapiens GN=LGALS1 PE=1 SV=2
158	sp|P15311|EZRI_HUMAN	Ezrin OS=Homo sapiens GN=EZR PE=1 SV=4
159	sp|P0C0L5|CO4B_HUMAN	Complement C4-B OS=Homo sapiens GN=C4B PE=1 SV=1
160	sp|P37840|SYUA_HUMAN	Alpha-synuclein OS=Homo sapiens GN=SNCA PE=1 SV=1
161	sp|P02042|HBD_HUMAN	Hemoglobin subunit delta OS=Homo sapiens GN=HBD PE=1 SV=2
162	sp|O76070|SYUG_HUMAN	Gamma-synuclein OS=Homo sapiens GN=SNCG PE=1 SV=2
163	sp|Q08209|PP2BA_HUMAN	Serine/threonine-protein phosphatase 2B catalytic subunit alpha isoform OS=Homo sapiens GN
164	sp|P01834|IGKC_HUMAN	Ig kappa chain C region OS=Homo sapiens GN=IGKC PE=1 SV=1
165	sp|P30043|BLVRB_HUMAN	Flavin reductase OS=Homo sapiens GN=BLVRB PE=1 SV=3
166	sp|P05109|S10A8_HUMAN	Protein S100-A8 OS=Homo sapiens GN=S100A8 PE=1 SV=1
167	sp|P61266|STX1B_HUMAN	Syntaxin-1B OS=Homo sapiens GN=STX1B PE=2 SV=1
168	sp|P37802|TAGL2_HUMAN	Transgelin-2 OS=Homo sapiens GN=TAGLN2 PE=1 SV=3
169	sp|Q16658|FSCN1_HUMAN	Fascin OS=Homo sapiens GN=FSCN1 PE=1 SV=3
170	sp|P22392-2|NDKB_HUMAN	Isoform NM23-LV of Nucleoside diphosphate kinase B OS=Homo sapiens GN=NME2
171	sp|Q14697|GANAB_HUMAN	Neutral alpha-glucosidase AB OS=Homo sapiens GN=GANAB PE=1 SV=3
172	sp|P60660|MYL6_HUMAN	Myosin light polypeptide 6 OS=Homo sapiens GN=MYL6 PE=1 SV=2
173	sp|P06702|S10A9_HUMAN	Protein S100-A9 OS=Homo sapiens GN=S100A9 PE=1 SV=1
174	sp|P52209|6PGD_HUMAN	6-phosphogluconate dehydrogenase, decarboxylating OS=Homo sapiens GN=PGD PE=1 SV=3
175	sp|P61978|HNRPK_HUMAN	Heterogeneous nuclear ribonucleoprotein K OS=Homo sapiens GN=HNRNPK PE=1 SV=1
176	sp|P04792|HSPB1_HUMAN	Heat shock protein beta-1 OS=Homo sapiens GN=HSPB1 PE=1 SV=2
177	sp|P09622|DLDH_HUMAN	Dihydrolipoyl dehydrogenase, mitochondrial OS=Homo sapiens GN=DLD PE=1 SV=2
178	sp|P21579|SYT1_HUMAN	Synaptotagmin-1 OS=Homo sapiens GN=SYT1 PE=1 SV=1
179	sp|P01842|LAC_HUMAN	Ig lambda chain C regions OS=Homo sapiens GN=IGLC1 PE=1 SV=1
180	sp|P36543|VATE1_HUMAN	V-type proton ATPase subunit E 1 OS=Homo sapiens GN=ATP6V1E1 PE=1 SV=1
181	sp|P13797|PLST_HUMAN	Plastin-3 OS=Homo sapiens GN=PLS3 PE=1 SV=4
182	sp|Q04917|1433F_HUMAN	14-3-3 protein eta OS=Homo sapiens GN=YWHAH PE=1 SV=4
183	sp|P30044|PRDX5_HUMAN	Peroxiredoxin-5, mitochondrial OS=Homo sapiens GN=PRDX5 PE=1 SV=3
184	sp|P14314|GLU2B_HUMAN	Glucosidase 2 subunit beta OS=Homo sapiens GN=PRKCSH PE=1 SV=2
185	sp|P36871|PGM1_HUMAN	Phosphoglucomutase-1 OS=Homo sapiens GN=PGM1 PE=1 SV=3
186	sp|P09429|HMGB1_HUMAN	High mobility group protein B1 OS=Homo sapiens GN=HMGB1 PE=1 SV=3
187	sp|P36222|CH3L1_HUMAN	Chitinase-3-like protein 1 OS=Homo sapiens GN=CHI3L1 PE=1 SV=2
188	sp|P02765|FETUA_HUMAN	Alpha-2-HS-glycoprotein OS=Homo sapiens GN=AHSG PE=1 SV=1
189	sp|Q02952-3|AKA12_HUMAN	Isoform Gamma of A-kinase anchor protein 12 OS=Homo sapiens GN=AKAP12
190	sp|P12532|KCRU_HUMAN	Creatine kinase U-type, mitochondrial OS=Homo sapiens GN=CKMT1A PE=1 SV=1
191	sp|P08603|CFAH_HUMAN	Complement factor H OS=Homo sapiens GN=CFH PE=1 SV=4
192	sp|P27348|1433T_HUMAN	14-3-3 protein theta OS=Homo sapiens GN=YWHAQ PE=1 SV=1

Master N	Accession	Name
193	sp|P07339|CATD_HUMAN	Cathepsin D OS=Homo sapiens GN=CTSD PE=1 SV=1
194	sp|P30042|ES1_HUMAN	ES1 protein homolog, mitochondrial OS=Homo sapiens GN=C21orf33 PE=1 SV=3
195	sp|Q9BPU6|DPYL5_HUMAN	Dihydropyrimidinase-related protein 5 OS=Homo sapiens GN=DPYSL5 PE=1 SV=1
196	sp|O00499|BIN1_HUMAN	Myc box-dependent-interacting protein 1 OS=Homo sapiens GN=BIN1 PE=1 SV=1
197	sp|P60842|IF4A1_HUMAN	Eukaryotic initiation factor 4A-I OS=Homo sapiens GN=EIF4A1 PE=1 SV=1
198	sp|P01008|ANT3_HUMAN	Antithrombin-III OS=Homo sapiens GN=SERPINC1 PE=1 SV=1
199	sp|P67936|TPM4_HUMAN	Tropomyosin alpha-4 chain OS=Homo sapiens GN=TPM4 PE=1 SV=3
200	sp|P10909-2|CLUS_HUMAN	Isoform 2 of Clusterin OS=Homo sapiens GN=CLU
201	sp|P60880|SNP25_HUMAN	Synaptosomal-associated protein 25 OS=Homo sapiens GN=SNAP25 PE=1 SV=1
202	sp|Q14194|DPYL1_HUMAN	Dihydropyrimidinase-related protein 1 OS=Homo sapiens GN=CRMP1 PE=1 SV=1
203	sp|P37837|TALDO_HUMAN	Transaldolase OS=Homo sapiens GN=TALDO1 PE=1 SV=2
204	sp|P05155|IC1_HUMAN	Plasma protease C1 inhibitor OS=Homo sapiens GN=SERPING1 PE=1 SV=2
205	sp|P04080|CYTB_HUMAN	Cystatin-B OS=Homo sapiens GN=CSTB PE=1 SV=2
206	sp|P62988|UBIQ_HUMAN	Ubiquitin OS=Homo sapiens GN=RPS27A PE=1 SV=1
207	sp|P05091|ALDH2_HUMAN	Aldehyde dehydrogenase, mitochondrial OS=Homo sapiens GN=ALDH2 PE=1 SV=2
208	sp|Q96JE9|MAP6_HUMAN	Microtubule-associated protein 6 OS=Homo sapiens GN=MAP6 PE=1 SV=2
209	sp|O43301|HS12A_HUMAN	Heat shock 70 kDa protein 12A OS=Homo sapiens GN=HSPA12A PE=1 SV=2
210	sp|O43852|CALU_HUMAN	Calumenin OS=Homo sapiens GN=CALU PE=1 SV=2
211	sp|Q99962|SH3G2_HUMAN	Endophilin-A1 OS=Homo sapiens GN=SH3GL2 PE=1 SV=1
212	sp|P21291|CSRP1_HUMAN	Cysteine and glycine-rich protein 1 OS=Homo sapiens GN=CSRP1 PE=1 SV=3
213	sp|P10412|H14_HUMAN	Histone H1.4 OS=Homo sapiens GN=HIST1H1E PE=1 SV=2
214	sp|P27824|CALX_HUMAN	Calnexin OS=Homo sapiens GN=CANX PE=1 SV=2
215	sp|P02794|FRIH_HUMAN	Ferritin heavy chain OS=Homo sapiens GN=FTH1 PE=1 SV=2
216	sp|P47756-2|CAPZB_HUMAN	Isoform 2 of F-actin-capping protein subunit beta OS=Homo sapiens GN=CAPZB
217	sp|Q13509|TBB3_HUMAN	Tubulin beta-3 chain OS=Homo sapiens GN=TUBB3 PE=1 SV=2
218	sp|P52565|GDIR1_HUMAN	Rho GDP-dissociation inhibitor 1 OS=Homo sapiens GN=ARHGDIA PE=1 SV=3
219	sp|P00492|HPRT_HUMAN	Hypoxanthine-guanine phosphoribosyltransferase OS=Homo sapiens GN=HPRT1 PE=1 SV=2
220	sp|P07858|CATB_HUMAN	Cathepsin B OS=Homo sapiens GN=CTSB PE=1 SV=3
221	sp|P02652|APOA2_HUMAN	Apolipoprotein A-II OS=Homo sapiens GN=APOA2 PE=1 SV=1
222	sp|Q9Y617|SERC_HUMAN	Phosphoserine aminotransferase OS=Homo sapiens GN=PSAT1 PE=1 SV=2
223	sp|Q01484-5|ANK2_HUMAN	Isoform 4 of Ankyrin-2 OS=Homo sapiens GN=ANK2
224	sp|P22061|PIMT_HUMAN	Protein-L-isoaspartate(D-aspartate) O-methyltransferase OS=Homo sapiens GN=PCMT1 PE=1
225	sp|P99999|CYC_HUMAN	Cytochrome c OS=Homo sapiens GN=CYCS PE=1 SV=2
226	sp|Q16698|DECR_HUMAN	2,4-dienoyl-CoA reductase, mitochondrial OS=Homo sapiens GN=DECR1 PE=1 SV=1
227	sp|P07477|TRY1_HUMAN	Trypsin-1 OS=Homo sapiens GN=PRSS1 PE=1 SV=1
228	sp|P31946|1433B_HUMAN	14-3-3 protein beta/alpha OS=Homo sapiens GN=YWHAB PE=1 SV=3
229	sp|P50990|TCPQ_HUMAN	T-complex protein 1 subunit theta OS=Homo sapiens GN=CCT8 PE=1 SV=4
230	sp|P40939|ECHA_HUMAN	Trifunctional enzyme subunit alpha, mitochondrial OS=Homo sapiens GN=HADHA PE=1 SV=2
231	sp|P02649|APOE_HUMAN	Apolipoprotein E OS=Homo sapiens GN=APOE PE=1 SV=1
232	sp|P49006|MRP_HUMAN	MARCKS-related protein OS=Homo sapiens GN=MARCKSL1 PE=1 SV=2
233	sp|P06454|PTMA_HUMAN	Prothymosin alpha OS=Homo sapiens GN=PTMA PE=1 SV=2
234	sp|P62805|H4_HUMAN	Histone H4 OS=Homo sapiens GN=HIST1H4A PE=1 SV=2
235	sp|Q96GW7|PGCB_HUMAN	Brevican core protein OS=Homo sapiens GN=BCAN PE=1 SV=2
236	sp|P49773|HINT1_HUMAN	Histidine triad nucleotide-binding protein 1 OS=Homo sapiens GN=HINT1 PE=1 SV=2
237	sp|Q01105-2|SET_HUMAN	Isoform TAF-I beta of Protein SET OS=Homo sapiens GN=SET
238	sp|Q01518|CAP1_HUMAN	Adenylyl cyclase-associated protein 1 OS=Homo sapiens GN=CAP1 PE=1 SV=4

Master N	Accession	Name
239	sp|P30153|2AAA_HUMAN	Serine/threonine-protein phosphatase 2A 65 kDa regulatory subunit A alpha isoform OS=Homo
240	sp|P50991|TCPD_HUMAN	T-complex protein 1 subunit delta OS=Homo sapiens GN=CCT4 PE=1 SV=4
241	sp|Q09666|AHNK_HUMAN	Neuroblast differentiation-associated protein AHNAK OS=Homo sapiens GN=AHNAK PE=1 SV
242	sp|O15540|FABP7_HUMAN	Fatty acid-binding protein, brain OS=Homo sapiens GN=FABP7 PE=1 SV=3
243	sp|P67809|YBOX1_HUMAN	Nuclease-sensitive element-binding protein 1 OS=Homo sapiens GN=YBX1 PE=1 SV=3
244	sp|P30048|PRDX3_HUMAN	Thioredoxin-dependent peroxide reductase, mitochondrial OS=Homo sapiens GN=PRDX3 PE=
245	sp|P47755|CAZA2_HUMAN	F-actin-capping protein subunit alpha-2 OS=Homo sapiens GN=CAPZA2 PE=1 SV=3
246	sp|Q04760|LGUL_HUMAN	Lactoylglutathione lyase OS=Homo sapiens GN=GLO1 PE=1 SV=4
247	sp|P24752|THIL_HUMAN	Acetyl-CoA acetyltransferase, mitochondrial OS=Homo sapiens GN=ACAT1 PE=1 SV=1
248	sp|O43491|E41L2_HUMAN	Band 4.1-like protein 2 OS=Homo sapiens GN=EPB41L2 PE=1 SV=1
249	sp|Q15121|PEA15_HUMAN	Astrocytic phosphoprotein PEA-15 OS=Homo sapiens GN=PEA15 PE=1 SV=2
250	sp|P55786|PSA_HUMAN	Puromycin-sensitive aminopeptidase OS=Homo sapiens GN=NPEPPS PE=1 SV=2
251	sp|O60641-3|AP180_HUMAN	Isoform 3 of Clathrin coat assembly protein AP180 OS=Homo sapiens GN=SNAP91
252	sp|P78417|GSTO1_HUMAN	Glutathione S-transferase omega-1 OS=Homo sapiens GN=GSTO1 PE=1 SV=2
253	sp|Q96FJ2|DYL2_HUMAN	Dynein light chain 2, cytoplasmic OS=Homo sapiens GN=DYNLL2 PE=1 SV=1
254	sp|P10599|THIO_HUMAN	Thioredoxin OS=Homo sapiens GN=TXN PE=1 SV=3
255	sp|P61158|ARP3_HUMAN	Actin-related protein 3 OS=Homo sapiens GN=ACTR3 PE=1 SV=3
256	sp|P25786|PSA1_HUMAN	Proteasome subunit alpha type-1 OS=Homo sapiens GN=PSMA1 PE=1 SV=1
257	sp|O75083|WDR1_HUMAN	WD repeat-containing protein 1 OS=Homo sapiens GN=WDR1 PE=1 SV=4
258	sp|O15144|ARPC2_HUMAN	Actin-related protein 2/3 complex subunit 2 OS=Homo sapiens GN=ARPC2 PE=1 SV=1
259	sp|P09493-4|TPM1_HUMAN	Isoform 4 of Tropomyosin alpha-1 chain OS=Homo sapiens GN=TPM1
260	sp|P63241-2|IF5A1_HUMAN	Isoform A of Eukaryotic translation initiation factor 5A-1 OS=Homo sapiens GN=EIF5A
261	sp|Q9H115|SNAB_HUMAN	Beta-soluble NSF attachment protein OS=Homo sapiens GN=NAPB PE=1 SV=2
262	sp|P78371|TCPB_HUMAN	T-complex protein 1 subunit beta OS=Homo sapiens GN=CCT2 PE=1 SV=4
263	sp|Q14103-3|HNRPD_HUMAN	Isoform Dx7 of Heterogeneous nuclear ribonucleoprotein D0 OS=Homo sapiens GN=HNRNPD
264	sp|Q99719|SEPT5_HUMAN	Septin-5 OS=Homo sapiens GN=SEPT5 PE=1 SV=1
265	sp|P20810-7|ICAL_HUMAN	Isoform 7 of Calpastatin OS=Homo sapiens GN=CAST
266	sp|P51649|SSDH_HUMAN	Succinate-semialdehyde dehydrogenase, mitochondrial OS=Homo sapiens GN=ALDH5A1 PE=
267	sp|P36957|ODO2_HUMAN	Dihydrolipoyllysine-residue succinyltransferase component of 2-oxoglutarate dehydrogenase co
268	sp|P17931|LEG3_HUMAN	Galectin-3 OS=Homo sapiens GN=LGALS3 PE=1 SV=5
269	sp|P02751-4|FINC_HUMAN	Isoform Fibronectin III-15X of Fibronectin OS=Homo sapiens GN=FN1
270	sp|P00488|F13A_HUMAN	Coagulation factor XIII A chain OS=Homo sapiens GN=F13A1 PE=1 SV=4
271	sp|P09496|CLCA_HUMAN	Clathrin light chain A OS=Homo sapiens GN=CLTA PE=1 SV=1
272	sp|Q13642|FHL1_HUMAN	Four and a half LIM domains protein 1 OS=Homo sapiens GN=FHL1 PE=1 SV=4
273	sp|P63208|SKP1_HUMAN	S-phase kinase-associated protein 1 OS=Homo sapiens GN=SKP1 PE=1 SV=2
274	sp|P62826|RAN_HUMAN	GTP-binding nuclear protein Ran OS=Homo sapiens GN=RAN PE=1 SV=3
275	sp|P30038|AL4A1_HUMAN	Delta-1-pyrroline-5-carboxylate dehydrogenase, mitochondrial OS=Homo sapiens GN=ALDH4A
276	sp|P07602-2|SAP_HUMAN	Isoform Sap-mu-6 of Proactivator polypeptide OS=Homo sapiens GN=PSAP
277	sp|P09651|ROA1_HUMAN	Heterogeneous nuclear ribonucleoprotein A1 OS=Homo sapiens GN=HNRNPA1 PE=1 SV=5
278	sp|P49419|AL7A1_HUMAN	Alpha-aminoadipic semialdehyde dehydrogenase OS=Homo sapiens GN=ALDH7A1 PE=1 SV=
279	sp|P05455|LA_HUMAN	Lupus La protein OS=Homo sapiens GN=SSB PE=1 SV=2
280	sp|Q16643-2|DREB_HUMAN	Isoform 2 of Drebrin OS=Homo sapiens GN=DBN1
281	sp|Q14894|CRYM_HUMAN	Mu-crystallin homolog OS=Homo sapiens GN=CRYM PE=1 SV=1
282	sp|O00764|PDXK_HUMAN	Pyridoxal kinase OS=Homo sapiens GN=PDXK PE=1 SV=1
283	sp|P23471-2|PTPRZ_HUMAN	Isoform Short of Receptor-type tyrosine-protein phosphatase zeta OS=Homo sapiens GN=PTP
284	sp|Q01469|FABP5_HUMAN	Fatty acid-binding protein, epidermal OS=Homo sapiens GN=FABP5 PE=1 SV=3
285	sp|P62760|VISL1_HUMAN	Visinin-like protein 1 OS=Homo sapiens GN=VSNL1 PE=1 SV=2
286	sp|P07437|TBB5_HUMAN	Tubulin beta chain OS=Homo sapiens GN=TUBB PE=1 SV=2

Master N	Accession	Name
287	sp|P68366|TBA4A_HUMAN	Tubulin alpha-4A chain OS=Homo sapiens GN=TUBA4A PE=1 SV=1
288	sp|P19652|A1AG2_HUMAN	Alpha-1-acid glycoprotein 2 OS=Homo sapiens GN=ORM2 PE=1 SV=2
289	sp|P04196|HRG_HUMAN	Histidine-rich glycoprotein OS=Homo sapiens GN=HRG PE=1 SV=1
290	sp|P20962|PTMS_HUMAN	Parathymosin OS=Homo sapiens GN=PTMS PE=1 SV=2
291	sp|Q13561|DCTN2_HUMAN	Dynactin subunit 2 OS=Homo sapiens GN=DCTN2 PE=1 SV=4
292	sp|P01019|ANGT_HUMAN	Angiotensinogen OS=Homo sapiens GN=AGT PE=1 SV=1
293	sp|P58546|MTPN_HUMAN	Myotrophin OS=Homo sapiens GN=MTPN PE=1 SV=2
294	sp|P00751|CFAB_HUMAN	Complement factor B OS=Homo sapiens GN=CFB PE=1 SV=2
295	sp|Q5QNW6|H2B2F_HUMAN	Histone H2B type 2-F OS=Homo sapiens GN=HIST2H2BF PE=1 SV=3
296	sp|P13010|XRCC5_HUMAN	X-ray repair cross-complementing protein 5 OS=Homo sapiens GN=XRCC5 PE=1 SV=3
297	sp|P31943|HNRH1_HUMAN	Heterogeneous nuclear ribonucleoprotein H OS=Homo sapiens GN=HNRNPH1 PE=1 SV=4
298	sp|P61626|LYSC_HUMAN	Lysozyme C OS=Homo sapiens GN=LYZ PE=1 SV=1
299	sp|P28838|AMPL_HUMAN	Cytosol aminopeptidase OS=Homo sapiens GN=LAP3 PE=1 SV=3
300	sp|Q16695|H31T_HUMAN	Histone H3.1t OS=Homo sapiens GN=HIST3H3 PE=1 SV=3
301	sp|P45974-2|UBP5_HUMAN	Isoform Short of Ubiquitin carboxyl-terminal hydrolase 5 OS=Homo sapiens GN=USP5
302	sp|P49418|AMPH_HUMAN	Amphiphysin OS=Homo sapiens GN=AMPH PE=1 SV=1
303	sp|Q13011|ECH1_HUMAN	Delta(3,5)-Delta(2,4)-dienoyl-CoA isomerase, mitochondrial OS=Homo sapiens GN=ECH1 PE=
304	sp|P07910|HNRPC_HUMAN	Heterogeneous nuclear ribonucleoproteins C1/C2 OS=Homo sapiens GN=HNRNPC PE=1 SV=
305	sp|P07196|NFL_HUMAN	Neurofilament light polypeptide OS=Homo sapiens GN=NEFL PE=1 SV=3
306	sp|O75368|SH3L1_HUMAN	SH3 domain-binding glutamic acid-rich-like protein OS=Homo sapiens GN=SH3BGRL PE=1 SV
307	sp|Q01813|K6PP_HUMAN	6-phosphofructokinase type C OS=Homo sapiens GN=PFKP PE=1 SV=2
308	sp|Q8N573-5|OXR1_HUMAN	Isoform 5 of Oxidation resistance protein 1 OS=Homo sapiens GN=OXR1
309	sp|Q14204|DYHC1_HUMAN	Cytoplasmic dynein 1 heavy chain 1 OS=Homo sapiens GN=DYNC1H1 PE=1 SV=5
310	sp|P09497|CLCB_HUMAN	Clathrin light chain B OS=Homo sapiens GN=CLTB PE=1 SV=1
311	sp|P02545|LMNA_HUMAN	Lamin-A/C OS=Homo sapiens GN=LMNA PE=1 SV=1
312	sp|P61026|RAB10_HUMAN	Ras-related protein Rab-10 OS=Homo sapiens GN=RAB10 PE=1 SV=1
313	sp|P13804|ETFA_HUMAN	Electron transfer flavoprotein subunit alpha, mitochondrial OS=Homo sapiens GN=ETFA PE=1
314	sp|Q8NFI4|F10A5_HUMAN	Putative protein FAM10A5 OS=Homo sapiens GN=FAM10A5 PE=5 SV=1
315	sp|O95782-2|AP2A1_HUMAN	Isoform B of AP-2 complex subunit alpha-1 OS=Homo sapiens GN=AP2A1
316	sp|P63000|RAC1_HUMAN	Ras-related C3 botulinum toxin substrate 1 OS=Homo sapiens GN=RAC1 PE=1 SV=1
317	sp|P62873|GBB1_HUMAN	Guanine nucleotide-binding protein G(I)/G(S)/G(T) subunit beta-1 OS=Homo sapiens GN=GNB
318	sp|Q92777|SYN2_HUMAN	Synapsin-2 OS=Homo sapiens GN=SYN2 PE=1 SV=3
319	sp|P55809|SCOT1_HUMAN	Succinyl-CoA:3-ketoacid-coenzyme A transferase 1, mitochondrial OS=Homo sapiens GN=OX
320	sp|P34932|HSP74_HUMAN	Heat shock 70 kDa protein 4 OS=Homo sapiens GN=HSPA4 PE=1 SV=4
321	sp|P35080|PROF2_HUMAN	Profilin-2 OS=Homo sapiens GN=PFN2 PE=1 SV=3
322	sp|P16104|H2AX_HUMAN	Histone H2A.x OS=Homo sapiens GN=H2AFX PE=1 SV=2
323	sp|P62857|RS28_HUMAN	40S ribosomal protein S28 OS=Homo sapiens GN=RPS28 PE=1 SV=1
324	sp|O75781|PALM_HUMAN	Paralemmin OS=Homo sapiens GN=PALM PE=1 SV=2
325	sp|P31146|COR1A_HUMAN	Coronin-1A OS=Homo sapiens GN=CORO1A PE=1 SV=4
326	sp|P06727|APOA4_HUMAN	Apolipoprotein A-IV OS=Homo sapiens GN=APOA4 PE=1 SV=3
327	sp|P49368|TCPG_HUMAN	T-complex protein 1 subunit gamma OS=Homo sapiens GN=CCT3 PE=1 SV=4
328	sp|O43175|SERA_HUMAN	D-3-phosphoglycerate dehydrogenase OS=Homo sapiens GN=PHGDH PE=1 SV=4
329	sp|P06748|NPM_HUMAN	Nucleophosmin OS=Homo sapiens GN=NPM1 PE=1 SV=2
330	sp|P35612|ADDB_HUMAN	Beta-adducin OS=Homo sapiens GN=ADD2 PE=1 SV=3
331	sp|P05387|RLA2_HUMAN	60S acidic ribosomal protein P2 OS=Homo sapiens GN=RPLP2 PE=1 SV=1
332	sp|P08559|ODPA_HUMAN	Pyruvate dehydrogenase E1 component subunit alpha, somatic form, mitochondrial OS=Homo
333	sp|Q14624-2|ITIH4_HUMAN	Isoform 2 of Inter-alpha-trypsin inhibitor heavy chain H4 OS=Homo sapiens GN=ITIH4
334	sp|P06703|S10A6_HUMAN	Protein S100-A6 OS=Homo sapiens GN=S100A6 PE=1 SV=1

Master N	Accession	Name
335	sp|P11177|ODPB_HUMAN	Pyruvate dehydrogenase E1 component subunit beta, mitochondrial OS=Homo sapiens GN=PD
336	sp|Q9UI12|VATH_HUMAN	V-type proton ATPase subunit H OS=Homo sapiens GN=ATP6V1H PE=1 SV=1
337	sp|Q13442|HAP28_HUMAN	28 kDa heat- and acid-stable phosphoprotein OS=Homo sapiens GN=PDAP1 PE=1 SV=1
338	sp|P62942|FKB1A_HUMAN	Peptidyl-prolyl cis-trans isomerase FKBP1A OS=Homo sapiens GN=FKBP1A PE=1 SV=2
339	sp|P60953-2|CDC42_HUMAN	Isoform Placental of Cell division control protein 42 homolog OS=Homo sapiens GN=CDC42
340	sp|Q05682-5|CALD1_HUMAN	Isoform HELA L-CAD II of Caldesmon OS=Homo sapiens GN=CALD1
341	sp|P22695|QCR2_HUMAN	Cytochrome b-c1 complex subunit 2, mitochondrial OS=Homo sapiens GN=UQCRC2 PE=1 SV
342	sp|P02743|SAMP_HUMAN	Serum amyloid P-component OS=Homo sapiens GN=APCS PE=1 SV=2
343	sp|P02749|APOH_HUMAN	Beta-2-glycoprotein 1 OS=Homo sapiens GN=APOH PE=1 SV=3
344	sp|Q9UH03-2|SEPT3_HUMAN	Isoform SEP3B of Neuronal-specific septin-3 OS=Homo sapiens GN=SEPT3
345	sp|P49748|ACADV_HUMAN	Very long-chain specific acyl-CoA dehydrogenase, mitochondrial OS=Homo sapiens GN=ACAD
346	sp|Q14847|LASP1_HUMAN	LIM and SH3 domain protein 1 OS=Homo sapiens GN=LASP1 PE=1 SV=2
347	sp|P00568|KAD1_HUMAN	Adenylate kinase isoenzyme 1 OS=Homo sapiens GN=AK1 PE=1 SV=3
348	sp|P61088|UBE2N_HUMAN	Ubiquitin-conjugating enzyme E2 N OS=Homo sapiens GN=UBE2N PE=1 SV=1
349	sp|P17858-2|K6PL_HUMAN	Isoform a of 6-phosphofructokinase, liver type OS=Homo sapiens GN=PFKL
350	sp|P29692-2|EF1D_HUMAN	Isoform 2 of Elongation factor 1-delta OS=Homo sapiens GN=EEF1D
351	sp|P30084|ECHM_HUMAN	Enoyl-CoA hydratase, mitochondrial OS=Homo sapiens GN=ECHS1 PE=1 SV=4
352	sp|P52566|GDIR2_HUMAN	Rho GDP-dissociation inhibitor 2 OS=Homo sapiens GN=ARHGDIB PE=1 SV=3
353	sp|P31937|3HIDH_HUMAN	3-hydroxyisobutyrate dehydrogenase, mitochondrial OS=Homo sapiens GN=HIBADH PE=1 SV
354	sp|P30626|SORCN_HUMAN	Sorcin OS=Homo sapiens GN=SRI PE=1 SV=1
355	sp|P01042-2|KNG1_HUMAN	Isoform LMW of Kininogen-1 OS=Homo sapiens GN=KNG1
356	sp|P17987|TCPA_HUMAN	T-complex protein 1 subunit alpha OS=Homo sapiens GN=TCP1 PE=1 SV=1
357	sp|Q9UEY8-2|ADDG_HUMAN	Isoform Short of Gamma-adducin OS=Homo sapiens GN=ADD3
358	sp|O14745|NHRF1_HUMAN	Na(+)/H(+) exchange regulatory cofactor NHE-RF1 OS=Homo sapiens GN=SLC9A3R1 PE=1 S
359	sp|Q16799|RTN1_HUMAN	Reticulon-1 OS=Homo sapiens GN=RTN1 PE=1 SV=1
360	sp|Q12765|SCRN1_HUMAN	Secernin-1 OS=Homo sapiens GN=SCRN1 PE=1 SV=2
361	sp|Q92752-2|TENR_HUMAN	Isoform 2 of Tenascin-R OS=Homo sapiens GN=TNR
362	sp|Q06323|PSME1_HUMAN	Proteasome activator complex subunit 1 OS=Homo sapiens GN=PSME1 PE=1 SV=1
363	sp|P27695|APEX1_HUMAN	DNA-(apurinic or apyrimidinic site) lyase OS=Homo sapiens GN=APEX1 PE=1 SV=2
364	sp|P23526|SAHH_HUMAN	Adenosylhomocysteinase OS=Homo sapiens GN=AHCY PE=1 SV=4
365	sp|Q16762|THTR_HUMAN	Thiosulfate sulfurtransferase OS=Homo sapiens GN=TST PE=1 SV=4
366	sp|P50213|IDH3A_HUMAN	Isocitrate dehydrogenase [NAD] subunit alpha, mitochondrial OS=Homo sapiens GN=IDH3A PE
367	sp|Q8TAM6|ERMIN_HUMAN	Ermin OS=Homo sapiens GN=ERMN PE=2 SV=1
368	sp|P59998|ARPC4_HUMAN	Actin-related protein 2/3 complex subunit 4 OS=Homo sapiens GN=ARPC4 PE=1 SV=3
369	sp|P05413|FABPH_HUMAN	Fatty acid-binding protein, heart OS=Homo sapiens GN=FABP3 PE=1 SV=4
370	sp|P35241|RADI_HUMAN	Radixin OS=Homo sapiens GN=RDX PE=1 SV=1
371	sp|P61204|ARF3_HUMAN	ADP-ribosylation factor 3 OS=Homo sapiens GN=ARF3 PE=1 SV=2
372	sp|Q9UJU6|DBNL_HUMAN	Drebrin-like protein OS=Homo sapiens GN=DBNL PE=1 SV=1
373	sp|P80188|NGAL_HUMAN	Neutrophil gelatinase-associated lipocalin OS=Homo sapiens GN=LCN2 PE=1 SV=2
374	sp|P18136|KV313_HUMAN	Ig kappa chain V-III region HIC OS=Homo sapiens PE=2 SV=1
375	sp|P80404|GABT_HUMAN	4-aminobutyrate aminotransferase, mitochondrial OS=Homo sapiens GN=ABAT PE=1 SV=3
376	sp|P51149|RAB7A_HUMAN	Ras-related protein Rab-7a OS=Homo sapiens GN=RAB7A PE=1 SV=1
377	sp|P13796|PLSL_HUMAN	Plastin-2 OS=Homo sapiens GN=LCP1 PE=1 SV=5
378	sp|Q99536|VAT1_HUMAN	Synaptic vesicle membrane protein VAT-1 homolog OS=Homo sapiens GN=VAT1 PE=1 SV=2
379	sp|Q15691|MARE1_HUMAN	Microtubule-associated protein RP/EB family member 1 OS=Homo sapiens GN=MAPRE1 PE=
380	sp|Q12905|ILF2_HUMAN	Interleukin enhancer-binding factor 2 OS=Homo sapiens GN=ILF2 PE=1 SV=2
381	sp|Q13228|SBP1_HUMAN	Selenium-binding protein 1 OS=Homo sapiens GN=SELENBP1 PE=1 SV=2
382	sp|P68036|UB2L3_HUMAN	Ubiquitin-conjugating enzyme E2 L3 OS=Homo sapiens GN=UBE2L3 PE=1 SV=1

Master N	Accession	Name
383	sp|P52272|HNRPM_HUMAN	Heterogeneous nuclear ribonucleoprotein M OS=Homo sapiens GN=HNRNPM PE=1 SV=3
384	sp|Q92686|NEUG_HUMAN	Neurogranin OS=Homo sapiens GN=NRGN PE=1 SV=1
385	sp|P14868|SYDC_HUMAN	Aspartyl-tRNA synthetase, cytoplasmic OS=Homo sapiens GN=DARS PE=1 SV=2
386	sp|Q16143|SYUB_HUMAN	Beta-synuclein OS=Homo sapiens GN=SNCB PE=1 SV=1
387	sp|P26583|HMGB2_HUMAN	High mobility group protein B2 OS=Homo sapiens GN=HMGB2 PE=1 SV=2
388	sp|Q14019|COTL1_HUMAN	Coactosin-like protein OS=Homo sapiens GN=COTL1 PE=1 SV=3
389	sp|P08865|RSSA_HUMAN	40S ribosomal protein SA OS=Homo sapiens GN=RPSA PE=1 SV=4
390	sp|P14174|MIF_HUMAN	Macrophage migration inhibitory factor OS=Homo sapiens GN=MIF PE=1 SV=4
391	sp|P68032|ACTC_HUMAN	Actin, alpha cardiac muscle 1 OS=Homo sapiens GN=ACTC1 PE=1 SV=1
392	sp|Q14240|IF4A2_HUMAN	Eukaryotic initiation factor 4A-II OS=Homo sapiens GN=EIF4A2 PE=1 SV=2
393	sp|Q9UN36-3|NDRG2_HUMAN	Isoform 3 of Protein NDRG2 OS=Homo sapiens GN=NDRG2
394	sp|P41222|PTGDS_HUMAN	Prostaglandin-H2 D-isomerase OS=Homo sapiens GN=PTGDS PE=1 SV=1
395	sp|P35637|FUS_HUMAN	RNA-binding protein FUS OS=Homo sapiens GN=FUS PE=1 SV=1
396	sp|P28331|NDUS1_HUMAN	NADH-ubiquinone oxidoreductase 75 kDa subunit, mitochondrial OS=Homo sapiens GN=NDUF
397	sp|P11310|ACADM_HUMAN	Medium-chain specific acyl-CoA dehydrogenase, mitochondrial OS=Homo sapiens GN=ACADM
398	sp|Q7Z6L0|PRRT2_HUMAN	Proline-rich transmembrane protein 2 OS=Homo sapiens GN=PRRT2 PE=2 SV=1
399	sp|P25311|ZA2G_HUMAN	Zinc-alpha-2-glycoprotein OS=Homo sapiens GN=AZGP1 PE=1 SV=2
400	sp|P10451|OSTP_HUMAN	Osteopontin OS=Homo sapiens GN=SPP1 PE=1 SV=1
401	sp|Q16623-2|STX1A_HUMAN	Isoform 1C of Syntaxin-1A OS=Homo sapiens GN=STX1A
402	sp|Q9UIJ7|KAD3_HUMAN	GTP:AMP phosphotransferase mitochondrial OS=Homo sapiens GN=AK3 PE=1 SV=4
403	sp|Q07021|C1QBP_HUMAN	Complement component 1 Q subcomponent-binding protein, mitochondrial OS=Homo sapiens
404	sp|Q99426|TBCB_HUMAN	Tubulin-folding cofactor B OS=Homo sapiens GN=TBCB PE=1 SV=2
405	sp|Q99714|HCD2_HUMAN	3-hydroxyacyl-CoA dehydrogenase type-2 OS=Homo sapiens GN=HSD17B10 PE=1 SV=3
406	sp|P02654|APOC1_HUMAN	Apolipoprotein C-I OS=Homo sapiens GN=APOC1 PE=1 SV=1
407	sp|P52943|CRIP2_HUMAN	Cysteine-rich protein 2 OS=Homo sapiens GN=CRIP2 PE=1 SV=1
408	sp|P50897|PPT1_HUMAN	Palmitoyl-protein thioesterase 1 OS=Homo sapiens GN=PPT1 PE=1 SV=1
409	sp|P27144|KAD4_HUMAN	Adenylate kinase isoenzyme 4, mitochondrial OS=Homo sapiens GN=AK3L1 PE=1 SV=1
410	sp|Q15843|NEDD8_HUMAN	NEDD8 OS=Homo sapiens GN=NEDD8 PE=1 SV=1
411	sp|P59666|DEF3_HUMAN	Neutrophil defensin 3 OS=Homo sapiens GN=DEFA3 PE=1 SV=1
412	sp|P19105|ML12A_HUMAN	Myosin regulatory light chain 12A OS=Homo sapiens GN=MYL12A PE=1 SV=2
413	sp|Q9H299|SH3L3_HUMAN	SH3 domain-binding glutamic acid-rich-like protein 3 OS=Homo sapiens GN=SH3BGRL3 PE=1
414	sp|P63027|VAMP2_HUMAN	Vesicle-associated membrane protein 2 OS=Homo sapiens GN=VAMP2 PE=1 SV=3
415	sp|P54578|UBP14_HUMAN	Ubiquitin carboxyl-terminal hydrolase 14 OS=Homo sapiens GN=USP14 PE=1 SV=3
416	sp|O14818|PSA7_HUMAN	Proteasome subunit alpha type-7 OS=Homo sapiens GN=PSMA7 PE=1 SV=1
417	sp|Q15555|MARE2_HUMAN	Microtubule-associated protein RP/EB family member 2 OS=Homo sapiens GN=MAPRE2 PE=
418	sp|Q8NCB2|CAMKV_HUMAN	CaM kinase-like vesicle-associated protein OS=Homo sapiens GN=CAMKV PE=2 SV=2
419	sp|Q15056|IF4H_HUMAN	Eukaryotic translation initiation factor 4H OS=Homo sapiens GN=EIF4H PE=1 SV=5
420	sp|Q15819|UB2V2_HUMAN	Ubiquitin-conjugating enzyme E2 variant 2 OS=Homo sapiens GN=UBE2V2 PE=1 SV=4
421	sp|Q9Y696|CLIC4_HUMAN	Chloride intracellular channel protein 4 OS=Homo sapiens GN=CLIC4 PE=1 SV=4
422	sp|Q9Y2T3|GUAD_HUMAN	Guanine deaminase OS=Homo sapiens GN=GDA PE=1 SV=1
423	sp|P05026|AT1B1_HUMAN	Sodium/potassium-transporting ATPase subunit beta-1 OS=Homo sapiens GN=ATP1B1 PE=1
424	sp|Q16352|AINX_HUMAN	Alpha-internexin OS=Homo sapiens GN=INA PE=1 SV=2
425	sp|P01034|CYTC_HUMAN	Cystatin-C OS=Homo sapiens GN=CST3 PE=1 SV=1
426	sp|P02656|APOC3_HUMAN	Apolipoprotein C-III OS=Homo sapiens GN=APOC3 PE=1 SV=1
427	sp|Q99584|S10AD_HUMAN	Protein S100-A13 OS=Homo sapiens GN=S100A13 PE=1 SV=1
428	sp|P02675|FIBB_HUMAN	Fibrinogen beta chain OS=Homo sapiens GN=FGB PE=1 SV=2
429	sp|P16070|CD44_HUMAN	CD44 antigen OS=Homo sapiens GN=CD44 PE=1 SV=2
430	sp|Q9BW30|TPPP3_HUMAN	Tubulin polymerization-promoting protein family member 3 OS=Homo sapiens GN=TPPP3 PE=

Master N	Accession	Name
431	sp|P05141|ADT2_HUMAN	ADP/ATP translocase 2 OS=Homo sapiens GN=SLC25A5 PE=1 SV=6
432	sp|Q9NRX4|PHP14_HUMAN	14 kDa phosphohistidine phosphatase OS=Homo sapiens GN=PHPT1 PE=1 SV=1
433	sp|P63098|CANB1_HUMAN	Calcineurin subunit B type 1 OS=Homo sapiens GN=PPP3R1 PE=1 SV=2
434	sp|P04271|S100B_HUMAN	Protein S100-B OS=Homo sapiens GN=S100B PE=1 SV=2
435	sp|Q9NY65|TBA8_HUMAN	Tubulin alpha-8 chain OS=Homo sapiens GN=TUBA8 PE=1 SV=1
436	sp|Q9UII2|ATIF1_HUMAN	ATPase inhibitor, mitochondrial OS=Homo sapiens GN=ATPIF1 PE=1 SV=1
437	sp|Q00796|DHSO_HUMAN	Sorbitol dehydrogenase OS=Homo sapiens GN=SORD PE=1 SV=4
438	sp|Q15366|PCBP2_HUMAN	Poly(rC)-binding protein 2 OS=Homo sapiens GN=PCBP2 PE=1 SV=1
439	sp|Q9Y5K8|VATD_HUMAN	V-type proton ATPase subunit D OS=Homo sapiens GN=ATP6V1D PE=1 SV=1
440	sp|Q92823-3|NRCAM_HUMAN	Isoform 3 of Neuronal cell adhesion molecule OS=Homo sapiens GN=NRCAM
441	sp|P12429|ANXA3_HUMAN	Annexin A3 OS=Homo sapiens GN=ANXA3 PE=1 SV=3
442	sp|P48643|TCPE_HUMAN	T-complex protein 1 subunit epsilon OS=Homo sapiens GN=CCT5 PE=1 SV=1
443	sp|P41250|SYG_HUMAN	Glycyl-tRNA synthetase OS=Homo sapiens GN=GARS PE=1 SV=2
444	sp|Q92599|SEPT8_HUMAN	Septin-8 OS=Homo sapiens GN=SEPT8 PE=1 SV=4
445	sp|Q9NUQ9|FA49B_HUMAN	Protein FAM49B OS=Homo sapiens GN=FAM49B PE=1 SV=1
446	sp|P38159|HNRPG_HUMAN	Heterogeneous nuclear ribonucleoprotein G OS=Homo sapiens GN=RBMX PE=1 SV=3
447	sp|P07954|FUMH_HUMAN	Fumarate hydratase, mitochondrial OS=Homo sapiens GN=FH PE=1 SV=3
448	sp|P43004|EAA2_HUMAN	Excitatory amino acid transporter 2 OS=Homo sapiens GN=SLC1A2 PE=1 SV=2
449	sp|P13489|RINI_HUMAN	Ribonuclease inhibitor OS=Homo sapiens GN=RNH1 PE=1 SV=2
450	sp|Q15417|CNN3_HUMAN	Calponin-3 OS=Homo sapiens GN=CNN3 PE=1 SV=1
451	sp|P43487|RANG_HUMAN	Ran-specific GTPase-activating protein OS=Homo sapiens GN=RANBP1 PE=1 SV=1
452	sp|P19823|ITIH2_HUMAN	Inter-alpha-trypsin inhibitor heavy chain H2 OS=Homo sapiens GN=ITIH2 PE=1 SV=2
453	sp|Q9P2R7-2|SUCB1_HUMAN	Isoform 2 of Succinyl-CoA ligase [ADP-forming] subunit beta, mitochondrial OS=Homo sapiens
454	sp|P50395|GDIB_HUMAN	Rab GDP dissociation inhibitor beta OS=Homo sapiens GN=GDI2 PE=1 SV=2
455	sp|P63313|TYB10_HUMAN	Thymosin beta-10 OS=Homo sapiens GN=TMSB10 PE=1 SV=2
456	sp|P04114|APOB_HUMAN	Apolipoprotein B-100 OS=Homo sapiens GN=APOB PE=1 SV=1
457	sp|Q9NZD2|GLTP_HUMAN	Glycolipid transfer protein OS=Homo sapiens GN=GLTP PE=1 SV=3
458	sp|P04004|VTNC_HUMAN	Vitronectin OS=Homo sapiens GN=VTN PE=1 SV=1
459	sp|Q14247|SRC8_HUMAN	Src substrate cortactin OS=Homo sapiens GN=CTTN PE=1 SV=2
460	sp|P23515|OMGP_HUMAN	Oligodendrocyte-myelin glycoprotein OS=Homo sapiens GN=OMG PE=1 SV=2
461	sp|P35237|SPB6_HUMAN	Serpin B6 OS=Homo sapiens GN=SERPINB6 PE=1 SV=3
462	sp|Q9Y4L1|HYOU1_HUMAN	Hypoxia up-regulated protein 1 OS=Homo sapiens GN=HYOU1 PE=1 SV=1
463	sp|Q9Y490|TLN1_HUMAN	Talin-1 OS=Homo sapiens GN=TLN1 PE=1 SV=3
464	sp|O94979-7|SC31A_HUMAN	Isoform 7 of Protein transport protein Sec31A OS=Homo sapiens GN=SEC31A
465	sp|P18206-2|VINC_HUMAN	Isoform Vinculin of Vinculin OS=Homo sapiens GN=VCL
466	sp|Q9UBQ7|GRHPR_HUMAN	Glyoxylate reductase/hydroxypyruvate reductase OS=Homo sapiens GN=GRHPR PE=1 SV=1
467	sp|Q15181|IPYR_HUMAN	Inorganic pyrophosphatase OS=Homo sapiens GN=PPA1 PE=1 SV=2
468	sp|P60520|GBRL2_HUMAN	Gamma-aminobutyric acid receptor-associated protein-like 2 OS=Homo sapiens GN=GABARAI
469	sp|Q9UL46|PSME2_HUMAN	Proteasome activator complex subunit 2 OS=Homo sapiens GN=PSME2 PE=1 SV=4
470	sp|P30085|KCY_HUMAN	UMP-CMP kinase OS=Homo sapiens GN=CMPK1 PE=1 SV=3
471	sp|P17612|KAPCA_HUMAN	cAMP-dependent protein kinase catalytic subunit alpha OS=Homo sapiens GN=PRKACA PE=1
472	sp|P49588|SYAC_HUMAN	Alanyl-tRNA synthetase, cytoplasmic OS=Homo sapiens GN=AARS PE=1 SV=2
473	sp|Q08380|LG3BP_HUMAN	Galectin-3-binding protein OS=Homo sapiens GN=LGALS3BP PE=1 SV=1
474	sp|P04217|A1BG_HUMAN	Alpha-1B-glycoprotein OS=Homo sapiens GN=A1BG PE=1 SV=3
475	sp|O75323|NIPS2_HUMAN	Protein NipSnap homolog 2 OS=Homo sapiens GN=GBAS PE=1 SV=1
476	sp|Q9UHD8|SEPT9_HUMAN	Septin-9 OS=Homo sapiens GN=SEPT9 PE=1 SV=2
477	sp|Q00839|HNRPU_HUMAN	Heterogeneous nuclear ribonucleoprotein U OS=Homo sapiens GN=HNRNPU PE=1 SV=6
478	sp|P05114|HMGN1_HUMAN	Non-histone chromosomal protein HMG-14 OS=Homo sapiens GN=HMGN1 PE=1 SV=3

Master N	Accession	Name
479	sp|Q14203-2|DCTN1_HUMAN	Isoform p135 of Dynactin subunit 1 OS=Homo sapiens GN=DCTN1
480	sp|O00299|CLIC1_HUMAN	Chloride intracellular channel protein 1 OS=Homo sapiens GN=CLIC1 PE=1 SV=4
481	sp|P00491|PNPH_HUMAN	Purine nucleoside phosphorylase OS=Homo sapiens GN=PNP PE=1 SV=2
482	sp|P19338|NUCL_HUMAN	Nucleolin OS=Homo sapiens GN=NCL PE=1 SV=3
483	sp|P20916|MAG_HUMAN	Myelin-associated glycoprotein OS=Homo sapiens GN=MAG PE=1 SV=1
484	sp|Q8N8S7|ENAH_HUMAN	Protein enabled homolog OS=Homo sapiens GN=ENAH PE=1 SV=2
485	sp|P23297|S10A1_HUMAN	Protein S100-A1 OS=Homo sapiens GN=S100A1 PE=1 SV=2
486	sp|P19827|ITIH1_HUMAN	Inter-alpha-trypsin inhibitor heavy chain H1 OS=Homo sapiens GN=ITIH1 PE=1 SV=3
487	sp|P04003|C4BPA_HUMAN	C4b-binding protein alpha chain OS=Homo sapiens GN=C4BPA PE=1 SV=2
488	sp|O75874|IDHC_HUMAN	Isocitrate dehydrogenase [NADP] cytoplasmic OS=Homo sapiens GN=IDH1 PE=1 SV=2
489	sp|Q14764|MVP_HUMAN	Major vault protein OS=Homo sapiens GN=MVP PE=1 SV=4
490	sp|Q99832|TCPH_HUMAN	T-complex protein 1 subunit eta OS=Homo sapiens GN=CCT7 PE=1 SV=2
491	sp|P12956|XRCC6_HUMAN	X-ray repair cross-complementing protein 6 OS=Homo sapiens GN=XRCC6 PE=1 SV=2
492	sp|P23588|IF4B_HUMAN	Eukaryotic translation initiation factor 4B OS=Homo sapiens GN=EIF4B PE=1 SV=2
493	sp|P53597|SUCA_HUMAN	Succinyl-CoA ligase [GDP-forming] subunit alpha, mitochondrial OS=Homo sapiens GN=SUCL
494	sp|P63220|RS21_HUMAN	40S ribosomal protein S21 OS=Homo sapiens GN=RPS21 PE=1 SV=1
495	sp|O00231|PSD11_HUMAN	26S proteasome non-ATPase regulatory subunit 11 OS=Homo sapiens GN=PSMD11 PE=1 SV
496	sp|P61586|RHOA_HUMAN	Transforming protein RhoA OS=Homo sapiens GN=RHOA PE=1 SV=1
497	sp|O14531|DPYL4_HUMAN	Dihydropyrimidinase-related protein 4 OS=Homo sapiens GN=DPYSL4 PE=1 SV=2
498	sp|P60983|GMFB_HUMAN	Glia maturation factor beta OS=Homo sapiens GN=GMFB PE=1 SV=2
499	sp|Q8NC51|PAIRB_HUMAN	Plasminogen activator inhibitor 1 RNA-binding protein OS=Homo sapiens GN=SERBP1 PE=1 S
500	sp|Q14257|RCN2_HUMAN	Reticulocalbin-2 OS=Homo sapiens GN=RCN2 PE=1 SV=1
501	sp|P46777|RL5_HUMAN	60S ribosomal protein L5 OS=Homo sapiens GN=RPL5 PE=1 SV=3
502	sp|P01597|KV105_HUMAN	Ig kappa chain V-I region DEE OS=Homo sapiens PE=1 SV=1
503	sp|P09493-5|TPM1_HUMAN	Isoform 5 of Tropomyosin alpha-1 chain OS=Homo sapiens GN=TPM1
504	sp|Q16775|GLO2_HUMAN	Hydroxyacylglutathione hydrolase, mitochondrial OS=Homo sapiens GN=HAGH PE=1 SV=2
505	sp|Q92597|NDRG1_HUMAN	Protein NDRG1 OS=Homo sapiens GN=NDRG1 PE=1 SV=1
506	sp|Q86V81|THOC4_HUMAN	THO complex subunit 4 OS=Homo sapiens GN=THOC4 PE=1 SV=3
507	sp|P23246|SFPQ_HUMAN	Splicing factor, proline- and glutamine-rich OS=Homo sapiens GN=SFPQ PE=1 SV=2
508	sp|P20618|PSB1_HUMAN	Proteasome subunit beta type-1 OS=Homo sapiens GN=PSMB1 PE=1 SV=2
509	sp|P43034|LIS1_HUMAN	Platelet-activating factor acetylhydrolase IB subunit alpha OS=Homo sapiens GN=PAFAH1B1 P
510	sp|O15511|ARPC5_HUMAN	Actin-related protein 2/3 complex subunit 5 OS=Homo sapiens GN=ARPC5 PE=1 SV=3
511	sp|Q9NQC3|RTN4_HUMAN	Reticulon-4 OS=Homo sapiens GN=RTN4 PE=1 SV=2
512	sp|Q86VP6-2|CAND1_HUMAN	Isoform 2 of Cullin-associated NEDD8-dissociated protein 1 OS=Homo sapiens GN=CAND1
513	sp|O14773|TPP1_HUMAN	Tripeptidyl-peptidase 1 OS=Homo sapiens GN=TPP1 PE=1 SV=2
514	sp|Q13200|PSMD2_HUMAN	26S proteasome non-ATPase regulatory subunit 2 OS=Homo sapiens GN=PSMD2 PE=1 SV=3
515	sp|P78356|PI42B_HUMAN	Phosphatidylinositol-5-phosphate 4-kinase type-2 beta OS=Homo sapiens GN=PIP4K2B PE=1
516	sp|O43390|HNRPR_HUMAN	Heterogeneous nuclear ribonucleoprotein R OS=Homo sapiens GN=HNRNPR PE=1 SV=1
517	sp|Q86SQ6|GP123_HUMAN	Probable G-protein coupled receptor 123 OS=Homo sapiens GN=GPR123 PE=2 SV=2
518	sp|Q9Y266|NUDC_HUMAN	Nuclear migration protein nudC OS=Homo sapiens GN=NUDC PE=1 SV=1
519	sp|P31949|S10AB_HUMAN	Protein S100-A11 OS=Homo sapiens GN=S100A11 PE=1 SV=2
520	sp|Q9GZP4|CA128_HUMAN	UPF0424 protein C1orf128 OS=Homo sapiens GN=C1orf128 PE=1 SV=1
521	sp|Q9UI15|TAGL3_HUMAN	Transgelin-3 OS=Homo sapiens GN=TAGLN3 PE=1 SV=2
522	sp|P00747|PLMN_HUMAN	Plasminogen OS=Homo sapiens GN=PLG PE=1 SV=2
523	sp|Q9NTK5|OLA1_HUMAN	Obg-like ATPase 1 OS=Homo sapiens GN=OLA1 PE=1 SV=2
524	sp|P09960|LKHA4_HUMAN	Leukotriene A-4 hydrolase OS=Homo sapiens GN=LTA4H PE=1 SV=2
525	sp|Q562R1|ACTBL_HUMAN	Beta-actin-like protein 2 OS=Homo sapiens GN=ACTBL2 PE=1 SV=2
526	sp|Q9HDC9|APMAP_HUMAN	Adipocyte plasma membrane-associated protein OS=Homo sapiens GN=APMAP PE=1 SV=2

Master N	Accession	Name
527	sp|P49189|AL9A1_HUMAN	4-trimethylaminobutyraldehyde dehydrogenase OS=Homo sapiens GN=ALDH9A1 PE=1 SV=3
528	sp|Q9UHG2|PCSK1_HUMAN	ProSAAS OS=Homo sapiens GN=PCSK1N PE=1 SV=1
529	sp|P62750|RL23A_HUMAN	60S ribosomal protein L23a OS=Homo sapiens GN=RPL23A PE=1 SV=1
530	sp|P02766|TTHY_HUMAN	Transthyretin OS=Homo sapiens GN=TTR PE=1 SV=1
531	sp|Q9HCC0|MCCB_HUMAN	Methylcrotonoyl-CoA carboxylase beta chain, mitochondrial OS=Homo sapiens GN=MCCC2 PE
532	sp|Q96LX7-2|CCD17_HUMAN	Isoform 2 of Coiled-coil domain-containing protein 17 OS=Homo sapiens GN=CCDC17
533	sp|P40121|CAPG_HUMAN	Macrophage-capping protein OS=Homo sapiens GN=CAPG PE=1 SV=1
534	sp|P52907|CAZA1_HUMAN	F-actin-capping protein subunit alpha-1 OS=Homo sapiens GN=CAPZA1 PE=1 SV=3
535	sp|P38117-2|ETFB_HUMAN	Isoform 2 of Electron transfer flavoprotein subunit beta OS=Homo sapiens GN=ETFB
536	sp|P30040|ERP29_HUMAN	Endoplasmic reticulum resident protein 29 OS=Homo sapiens GN=ERP29 PE=1 SV=4
537	sp|O43837|IDH3B_HUMAN	Isocitrate dehydrogenase [NAD] subunit beta, mitochondrial OS=Homo sapiens GN=IDH3B PE
538	sp|P21796|VDAC1_HUMAN	Voltage-dependent anion-selective channel protein 1 OS=Homo sapiens GN=VDAC1 PE=1 SV
539	sp|P07203|GPX1_HUMAN	Glutathione peroxidase 1 OS=Homo sapiens GN=GPX1 PE=1 SV=3
540	sp|Q04837|SSBP_HUMAN	Single-stranded DNA-binding protein, mitochondrial OS=Homo sapiens GN=SSBP1 PE=1 SV=
541	sp|P51991|ROA3_HUMAN	Heterogeneous nuclear ribonucleoprotein A3 OS=Homo sapiens GN=HNRNPA3 PE=1 SV=2
542	sp|O15061-2|SYNEM_HUMAN	Isoform Beta of Synemin OS=Homo sapiens GN=SYNM
543	sp|P34897|GLYM_HUMAN	Serine hydroxymethyltransferase, mitochondrial OS=Homo sapiens GN=SHMT2 PE=1 SV=3
544	sp|P02792|FRIL_HUMAN	Ferritin light chain OS=Homo sapiens GN=FTL PE=1 SV=2
545	sp|P02760|AMBP_HUMAN	Protein AMBP OS=Homo sapiens GN=AMBP PE=1 SV=1
546	sp|Q9H2D6-4|TARA_HUMAN	Isoform TRIOBP-4 of TRIO and F-actin-binding protein OS=Homo sapiens GN=TRIOBP
547	sp|Q16543|CDC37_HUMAN	Hsp90 co-chaperone Cdc37 OS=Homo sapiens GN=CDC37 PE=1 SV=1
548	sp|Q15293|RCN1_HUMAN	Reticulocalbin-1 OS=Homo sapiens GN=RCN1 PE=1 SV=1
549	sp|P06313|KV403_HUMAN	Ig kappa chain V-IV region JI OS=Homo sapiens PE=4 SV=1
550	sp|Q9BUF5|TBB6_HUMAN	Tubulin beta-6 chain OS=Homo sapiens GN=TUBB6 PE=1 SV=1
551	sp|P36776|LONM_HUMAN	Lon protease homolog, mitochondrial OS=Homo sapiens GN=LONP1 PE=1 SV=2
552	sp|Q96G03|PGM2_HUMAN	Phosphoglucomutase-2 OS=Homo sapiens GN=PGM2 PE=1 SV=4
553	sp|Q16836-2|HCDH_HUMAN	Isoform 2 of Hydroxyacyl-coenzyme A dehydrogenase, mitochondrial OS=Homo sapiens GN=H
554	sp|O14576-2|DC1I1_HUMAN	Isoform 2 of Cytoplasmic dynein 1 intermediate chain 1 OS=Homo sapiens GN=DYNC1I1
555	sp|P10768|ESTD_HUMAN	S-formylglutathione hydrolase OS=Homo sapiens GN=ESD PE=1 SV=2
556	sp|P62820|RAB1A_HUMAN	Ras-related protein Rab-1A OS=Homo sapiens GN=RAB1A PE=1 SV=3
557	sp|Q9UHD9|UBQL2_HUMAN	Ubiquilin-2 OS=Homo sapiens GN=UBQLN2 PE=1 SV=2
558	sp|P16401|H15_HUMAN	Histone H1.5 OS=Homo sapiens GN=HIST1H1B PE=1 SV=3
559	sp|O75533|SF3B1_HUMAN	Splicing factor 3B subunit 1 OS=Homo sapiens GN=SF3B1 PE=1 SV=3
560	sp|P62140|PP1B_HUMAN	Serine/threonine-protein phosphatase PP1-beta catalytic subunit OS=Homo sapiens GN=PPP1
561	sp|Q00765|REEP5_HUMAN	Receptor expression-enhancing protein 5 OS=Homo sapiens GN=REEP5 PE=1 SV=3
562	sp|A8MZA4|GG6L6_HUMAN	Putative golgin subfamily A member 6-like protein 6 OS=Homo sapiens PE=5 SV=2
563	sp|Q9UBB6-3|NCDN_HUMAN	Isoform 3 of Neurochondrin OS=Homo sapiens GN=NCDN
564	sp|Q9HCJ6|VAT1L_HUMAN	Synaptic vesicle membrane protein VAT-1 homolog-like OS=Homo sapiens GN=VAT1L PE=1 S
565	sp|Q13867|BLMH_HUMAN	Bleomycin hydrolase OS=Homo sapiens GN=BLMH PE=1 SV=1
566	sp|Q12906|ILF3_HUMAN	Interleukin enhancer-binding factor 3 OS=Homo sapiens GN=ILF3 PE=1 SV=3
567	sp|Q6YN16|HSDL2_HUMAN	Hydroxysteroid dehydrogenase-like protein 2 OS=Homo sapiens GN=HSDL2 PE=1 SV=1
568	sp|Q02750|MP2K1_HUMAN	Dual specificity mitogen-activated protein kinase kinase 1 OS=Homo sapiens GN=MAP2K1 PE
569	sp|Q8N335|GPD1L_HUMAN	Glycerol-3-phosphate dehydrogenase 1-like protein OS=Homo sapiens GN=GPD1L PE=1 SV=
570	sp|P59768|GBG2_HUMAN	Guanine nucleotide-binding protein G(I)/G(S)/G(O) subunit gamma-2 OS=Homo sapiens GN=G
571	sp|P78527|PRKDC_HUMAN	DNA-dependent protein kinase catalytic subunit OS=Homo sapiens GN=PRKDC PE=1 SV=3
572	sp|Q2KJY2-2|KI26B_HUMAN	Isoform 2 of Kinesin-like protein KIF26B OS=Homo sapiens GN=KIF26B
573	sp|Q9NP80|PLPL8_HUMAN	Calcium-independent phospholipase A2-gamma OS=Homo sapiens GN=PNPLA8 PE=1 SV=1
574	sp|Q5VTT5|MYOM3_HUMAN	Myomesin-3 OS=Homo sapiens GN=MYOM3 PE=2 SV=1

Master N	Accession	Name
575	sp|Q8N163|K1967_HUMAN	Protein KIAA1967 OS=Homo sapiens GN=KIAA1967 PE=1 SV=2
576	sp|P49821|NDUV1_HUMAN	NADH dehydrogenase [ubiquinone] flavoprotein 1, mitochondrial OS=Homo sapiens GN=NDUF
577	sp|P37235|HPCL1_HUMAN	Hippocalcin-like protein 1 OS=Homo sapiens GN=HPCAL1 PE=1 SV=3
578	sp|Q92973|TNPO1_HUMAN	Transportin-1 OS=Homo sapiens GN=TNPO1 PE=1 SV=2
579	sp|O43488|ARK72_HUMAN	Aflatoxin B1 aldehyde reductase member 2 OS=Homo sapiens GN=AKR7A2 PE=1 SV=3
580	sp|P11233|RALA_HUMAN	Ras-related protein Ral-A OS=Homo sapiens GN=RALA PE=1 SV=1
581	sp|Q9H444|CHM4B_HUMAN	Charged multivesicular body protein 4b OS=Homo sapiens GN=CHMP4B PE=1 SV=1
582	sp|Q9C0I9-4|LRC27_HUMAN	Isoform 4 of Leucine-rich repeat-containing protein 27 OS=Homo sapiens GN=LRRC27
583	sp|P23083|HV103_HUMAN	Ig heavy chain V-I region V35 OS=Homo sapiens PE=1 SV=1
584	sp|O43813|LANC1_HUMAN	LanC-like protein 1 OS=Homo sapiens GN=LANCL1 PE=1 SV=1
585	sp|P60201|MYPR_HUMAN	Myelin proteolipid protein OS=Homo sapiens GN=PLP1 PE=1 SV=2
586	sp|O75380|NDUS6_HUMAN	NADH dehydrogenase [ubiquinone] iron-sulfur protein 6, mitochondrial OS=Homo sapiens GN=
587	sp|P62851|RS25_HUMAN	40S ribosomal protein S25 OS=Homo sapiens GN=RPS25 PE=1 SV=1
588	sp|P63261|ACTG_HUMAN	Actin, cytoplasmic 2 OS=Homo sapiens GN=ACTG1 PE=1 SV=1
589	sp|P14618|KPYM_HUMAN	Pyruvate kinase isozymes M1/M2 OS=Homo sapiens GN=PKM2 PE=1 SV=4
590	sp|P68371|TBB2C_HUMAN	Tubulin beta-2C chain OS=Homo sapiens GN=TUBB2C PE=1 SV=1
591	sp|P10636-6|TAU_HUMAN	Isoform Tau-D of Microtubule-associated protein tau OS=Homo sapiens GN=MAPT
592	sp|P69892|HBG2_HUMAN	Hemoglobin subunit gamma-2 OS=Homo sapiens GN=HBG2 PE=1 SV=2
593	sp|Q05639|EF1A2_HUMAN	Elongation factor 1-alpha 2 OS=Homo sapiens GN=EEF1A2 PE=1 SV=1
594	sp|P15259|PGAM2_HUMAN	Phosphoglycerate mutase 2 OS=Homo sapiens GN=PGAM2 PE=1 SV=3
595	sp|Q08EQ4|TMSL1_HUMAN	Putative thymosin beta-4-like protein 1 OS=Homo sapiens GN=TMSL1 PE=5 SV=1
596	sp|P15531|NDKA_HUMAN	Nucleoside diphosphate kinase A OS=Homo sapiens GN=NME1 PE=1 SV=1
597	sp|Q14141|SEPT6_HUMAN	Septin-6 OS=Homo sapiens GN=SEPT6 PE=1 SV=4
598	sp|O14556|G3PT_HUMAN	Glyceraldehyde-3-phosphate dehydrogenase, testis-specific OS=Homo sapiens GN=GAPDHS
599	sp|P16298|PP2BB_HUMAN	Serine/threonine-protein phosphatase 2B catalytic subunit beta isoform OS=Homo sapiens GN=
600	sp|P54920|SNAA_HUMAN	Alpha-soluble NSF attachment protein OS=Homo sapiens GN=NAPA PE=1 SV=3
601	sp|Q92930|RAB8B_HUMAN	Ras-related protein Rab-8B OS=Homo sapiens GN=RAB8B PE=1 SV=2
602	sp|Q71UI9|H2AV_HUMAN	Histone H2A.V OS=Homo sapiens GN=H2AFV PE=1 SV=3
603	sp|P04899|GNAI2_HUMAN	Guanine nucleotide-binding protein G(i) subunit alpha-2 OS=Homo sapiens GN=GNAI2 PE=1 S
604	sp|Q9ULV4|COR1C_HUMAN	Coronin-1C OS=Homo sapiens GN=CORO1C PE=1 SV=1
605	sp|P01610|KV118_HUMAN	Ig kappa chain V-I region WEA OS=Homo sapiens PE=1 SV=1
606	sp|P04433|KV309_HUMAN	Ig kappa chain V-III region VG (Fragment) OS=Homo sapiens PE=1 SV=1
607	sp|O94919|ENDD1_HUMAN	Endonuclease domain-containing 1 protein OS=Homo sapiens GN=ENDOD1 PE=1 SV=2
608	sp|P01764|HV303_HUMAN	Ig heavy chain V-III region VH26 OS=Homo sapiens PE=1 SV=1
609	sp|P01031|CO5_HUMAN	Complement C5 OS=Homo sapiens GN=C5 PE=1 SV=4
610	sp|Q00577|PURA_HUMAN	Transcriptional activator protein Pur-alpha OS=Homo sapiens GN=PURA PE=1 SV=2
611	sp|Q96F85|CNRP1_HUMAN	CB1 cannabinoid receptor-interacting protein 1 OS=Homo sapiens GN=CNRIP1 PE=1 SV=1
612	sp|P60900|PSA6_HUMAN	Proteasome subunit alpha type-6 OS=Homo sapiens GN=PSMA6 PE=1 SV=1
613	sp|Q9BY66|KDM5D_HUMAN	Lysine-specific demethylase 5D OS=Homo sapiens GN=KDM5D PE=1 SV=2
614	sp|Q9H4M9|EHD1_HUMAN	EH domain-containing protein 1 OS=Homo sapiens GN=EHD1 PE=1 SV=2
615	sp|P55884|EIF3B_HUMAN	Eukaryotic translation initiation factor 3 subunit B OS=Homo sapiens GN=EIF3B PE=1 SV=3
616	sp|P05067|A4_HUMAN	Amyloid beta A4 protein OS=Homo sapiens GN=APP PE=1 SV=3
617	sp|Q9Y2X3|NOP58_HUMAN	Nucleolar protein 58 OS=Homo sapiens GN=NOP58 PE=1 SV=1
618	sp|P29218|IMPA1_HUMAN	Inositol monophosphatase 1 OS=Homo sapiens GN=IMPA1 PE=1 SV=1
619	sp|Q96DY2|IQCD_HUMAN	IQ domain-containing protein D OS=Homo sapiens GN=IQCD PE=2 SV=2
620	sp|Q96FF9|CDCA5_HUMAN	Sororin OS=Homo sapiens GN=CDCA5 PE=1 SV=1
621	sp|P22307|NLTP_HUMAN	Non-specific lipid-transfer protein OS=Homo sapiens GN=SCP2 PE=1 SV=2
622	sp|P11766|ADHX_HUMAN	Alcohol dehydrogenase class-3 OS=Homo sapiens GN=ADH5 PE=1 SV=4
623	sp|Q6NVY1|HIBCH_HUMAN	3-hydroxyisobutyryl-CoA hydrolase, mitochondrial OS=Homo sapiens GN=HIBCH PE=1 SV=2
624	sp|P61163|ACTZ_HUMAN	Alpha-centractin OS=Homo sapiens GN=ACTR1A PE=1 SV=1

Master N	Accession	Name
623	sp|Q6NVY1|HIBCH_HUMAN	3-hydroxyisobutyryl-CoA hydrolase, mitochondrial OS=Homo sapiens GN=HIBCH PE=1 SV=2
624	sp|P61163|ACTZ_HUMAN	Alpha-centractin OS=Homo sapiens GN=ACTR1A PE=1 SV=1
625	sp|O75347|TBCA_HUMAN	Tubulin-specific chaperone A OS=Homo sapiens GN=TBCA PE=1 SV=3
626	sp|P28070|PSB4_HUMAN	Proteasome subunit beta type-4 OS=Homo sapiens GN=PSMB4 PE=1 SV=4
627	sp|P13693|TCTP_HUMAN	Translationally-controlled tumor protein OS=Homo sapiens GN=TPT1 PE=1 SV=1
628	sp|Q15459|SF3A1_HUMAN	Splicing factor 3A subunit 1 OS=Homo sapiens GN=SF3A1 PE=1 SV=1
629	sp|Q8TCU5|NMD3A_HUMAN	Glutamate [NMDA] receptor subunit 3A OS=Homo sapiens GN=GRIN3A PE=1 SV=2
630	sp|Q02790|FKBP4_HUMAN	Peptidyl-prolyl cis-trans isomerase FKBP4 OS=Homo sapiens GN=FKBP4 PE=1 SV=3
631	sp|P10155|RO60_HUMAN	60 kDa SS-A/Ro ribonucleoprotein OS=Homo sapiens GN=TROVE2 PE=1 SV=2
632	sp|Q96PX6|CC85A_HUMAN	Coiled-coil domain-containing protein 85A OS=Homo sapiens GN=CCDC85A PE=2 SV=3
633	sp|Q8N1G4|LRC47_HUMAN	Leucine-rich repeat-containing protein 47 OS=Homo sapiens GN=LRRC47 PE=1 SV=1
634	sp|Q9Y3I0|CV028_HUMAN	UPF0027 protein C22orf28 OS=Homo sapiens GN=C22orf28 PE=1 SV=1
635	sp|Q9UNZ2|NSF1C_HUMAN	NSFL1 cofactor p47 OS=Homo sapiens GN=NSFL1C PE=1 SV=2
636	sp|P78324|SHPS1_HUMAN	Tyrosine-protein phosphatase non-receptor type substrate 1 OS=Homo sapiens GN=SIRPA PE
637	sp|P51884|LUM_HUMAN	Lumican OS=Homo sapiens GN=LUM PE=1 SV=2
638	sp|O43237|DC1L2_HUMAN	Cytoplasmic dynein 1 light intermediate chain 2 OS=Homo sapiens GN=DYNC1LI2 PE=1 SV=1
639	sp|P31930|QCR1_HUMAN	Cytochrome b-c1 complex subunit 1, mitochondrial OS=Homo sapiens GN=UQCRC1 PE=1 SV
640	sp|Q8TBX8|PI42C_HUMAN	Phosphatidylinositol-5-phosphate 4-kinase type-2 gamma OS=Homo sapiens GN=PIP4K2C PE
641	sp|Q495X7|TRI60_HUMAN	Tripartite motif-containing protein 60 OS=Homo sapiens GN=TRIM60 PE=2 SV=2
642	sp|Q99747|SNAG_HUMAN	Gamma-soluble NSF attachment protein OS=Homo sapiens GN=NAPG PE=1 SV=1
643	sp|P13716-2|HEM2_HUMAN	Isoform 2 of Delta-aminolevulinic acid dehydratase OS=Homo sapiens GN=ALAD
644	sp|Q9P0L0-2|VAPA_HUMAN	Isoform 2 of Vesicle-associated membrane protein-associated protein A OS=Homo sapiens GN
645	sp|Q8IW45|CARKD_HUMAN	Carbohydrate kinase domain-containing protein OS=Homo sapiens GN=CARKD PE=1 SV=1
646	sp|P07738|PMGE_HUMAN	Bisphosphoglycerate mutase OS=Homo sapiens GN=BPGM PE=1 SV=2
647	sp|P30049|ATPD_HUMAN	ATP synthase subunit delta, mitochondrial OS=Homo sapiens GN=ATP5D PE=1 SV=2
648	sp|Q9P2U7|VGLU1_HUMAN	Vesicular glutamate transporter 1 OS=Homo sapiens GN=SLC17A7 PE=2 SV=1
649	sp|P56211|ARP19_HUMAN	cAMP-regulated phosphoprotein 19 OS=Homo sapiens GN=ARPP19 PE=1 SV=2
650	sp|P29762|RABP1_HUMAN	Cellular retinoic acid-binding protein 1 OS=Homo sapiens GN=CRABP1 PE=1 SV=2
651	sp|P25713|MT3_HUMAN	Metallothionein-3 OS=Homo sapiens GN=MT3 PE=1 SV=1
652	sp|O95674|CDS2_HUMAN	Phosphatidate cytidylyltransferase 2 OS=Homo sapiens GN=CDS2 PE=1 SV=1
653	sp|Q9UPV7|K1045_HUMAN	Protein KIAA1045 OS=Homo sapiens GN=KIAA1045 PE=1 SV=2
654	sp|Q9H0R4|HDHD2_HUMAN	Haloacid dehalogenase-like hydrolase domain-containing protein 2 OS=Homo sapiens GN=HD
655	sp|P68402|PA1B2_HUMAN	Platelet-activating factor acetylhydrolase IB subunit beta OS=Homo sapiens GN=PAFAH1B2 PE
656	sp|P24534|EF1B_HUMAN	Elongation factor 1-beta OS=Homo sapiens GN=EEF1B2 PE=1 SV=3
657	sp|P67775|PP2AA_HUMAN	Serine/threonine-protein phosphatase 2A catalytic subunit alpha isoform OS=Homo sapiens GN
658	sp|P01766|HV305_HUMAN	Ig heavy chain V-III region BRO OS=Homo sapiens PE=1 SV=1
659	sp|Q9Y2Q3|GSTK1_HUMAN	Glutathione S-transferase kappa 1 OS=Homo sapiens GN=GSTK1 PE=1 SV=3
660	sp|Q9NPJ3|ACO13_HUMAN	Acyl-coenzyme A thioesterase 13 OS=Homo sapiens GN=ACOT13 PE=1 SV=1
661	sp|Q15370|ELOB_HUMAN	Transcription elongation factor B polypeptide 2 OS=Homo sapiens GN=TCEB2 PE=1 SV=1
662	sp|P35754|GLRX1_HUMAN	Glutaredoxin-1 OS=Homo sapiens GN=GLRX PE=1 SV=2
663	sp|P06310|KV206_HUMAN	Ig kappa chain V-II region RPMI 6410 OS=Homo sapiens PE=4 SV=1
664	sp|O60888|CUTA_HUMAN	Protein CutA OS=Homo sapiens GN=CUTA PE=1 SV=2
665	sp|P47985|UCRI_HUMAN	Cytochrome b-c1 complex subunit Rieske, mitochondrial OS=Homo sapiens GN=UQCRFS1 PE
666	sp|P02735|SAA_HUMAN	Serum amyloid A protein OS=Homo sapiens GN=SAA1 PE=1 SV=2
667	sp|P07451|CAH3_HUMAN	Carbonic anhydrase 3 OS=Homo sapiens GN=CA3 PE=1 SV=3
668	sp|O43399-4|TPD54_HUMAN	Isoform 4 of Tumor protein D54 OS=Homo sapiens GN=TPD52L2

Master N	Accession	Name
669	sp|P20336|RAB3A_HUMAN	Ras-related protein Rab-3A OS=Homo sapiens GN=RAB3A PE=1 SV=1
670	sp|P11413-3|G6PD_HUMAN	Isoform 3 of Glucose-6-phosphate 1-dehydrogenase OS=Homo sapiens GN=G6PD
671	sp|Q9HAV7|GRPE1_HUMAN	GrpE protein homolog 1, mitochondrial OS=Homo sapiens GN=GRPEL1 PE=1 SV=2
672	sp|O43615|TIM44_HUMAN	Mitochondrial import inner membrane translocase subunit TIM44 OS=Homo sapiens GN=TIMM
673	sp|P63010|AP2B1_HUMAN	AP-2 complex subunit beta OS=Homo sapiens GN=AP2B1 PE=1 SV=1
674	sp|Q9UK22|FBX2_HUMAN	F-box only protein 2 OS=Homo sapiens GN=FBXO2 PE=1 SV=2
675	sp|Q6IAA8|CK059_HUMAN	RhoA activator C11orf59 OS=Homo sapiens GN=C11orf59 PE=1 SV=2
676	sp|P21108|PRPS3_HUMAN	Ribose-phosphate pyrophosphokinase 3 OS=Homo sapiens GN=PRPS1L1 PE=1 SV=2
677	sp|Q02818|NUCB1_HUMAN	Nucleobindin-1 OS=Homo sapiens GN=NUCB1 PE=1 SV=4
678	sp|P15121|ALDR_HUMAN	Aldose reductase OS=Homo sapiens GN=AKR1B1 PE=1 SV=3
679	sp|P16402|H13_HUMAN	Histone H1.3 OS=Homo sapiens GN=HIST1H1D PE=1 SV=2
680	sp|Q8IYD1|ERF3B_HUMAN	Eukaryotic peptide chain release factor GTP-binding subunit ERF3B OS=Homo sapiens GN=G
681	sp|P43243|MATR3_HUMAN	Matrin-3 OS=Homo sapiens GN=MATR3 PE=1 SV=2
682	sp|P51808|DYLT3_HUMAN	Dynein light chain Tctex-type 3 OS=Homo sapiens GN=DYNLT3 PE=1 SV=1
683	sp|P51674|GPM6A_HUMAN	Neuronal membrane glycoprotein M6-a OS=Homo sapiens GN=GPM6A PE=1 SV=2
684	sp|Q96QK1|VPS35_HUMAN	Vacuolar protein sorting-associated protein 35 OS=Homo sapiens GN=VPS35 PE=1 SV=2
685	sp|Q969H8|CS010_HUMAN	UPF0556 protein C19orf10 OS=Homo sapiens GN=C19orf10 PE=1 SV=1
686	sp|P00403|COX2_HUMAN	Cytochrome c oxidase subunit 2 OS=Homo sapiens GN=MT-CO2 PE=1 SV=1
687	sp|Q15185|TEBP_HUMAN	Prostaglandin E synthase 3 OS=Homo sapiens GN=PTGES3 PE=1 SV=1
688	sp|Q9H4G0-3|E41L1_HUMAN	Isoform 3 of Band 4.1-like protein 1 OS=Homo sapiens GN=EPB41L1
689	sp|O75521|PECI_HUMAN	Peroxisomal 3,2-trans-enoyl-CoA isomerase OS=Homo sapiens GN=PECI PE=1 SV=4
690	sp|P30405|PPIF_HUMAN	Peptidyl-prolyl cis-trans isomerase F, mitochondrial OS=Homo sapiens GN=PPIF PE=1 SV=1
691	sp|Q00688|FKBP3_HUMAN	Peptidyl-prolyl cis-trans isomerase FKBP3 OS=Homo sapiens GN=FKBP3 PE=1 SV=1
692	sp|O95292|VAPB_HUMAN	Vesicle-associated membrane protein-associated protein B/C OS=Homo sapiens GN=VAPB PE
693	sp|O43447|PPIH_HUMAN	Peptidyl-prolyl cis-trans isomerase H OS=Homo sapiens GN=PPIH PE=1 SV=1
694	sp|Q13185|CBX3_HUMAN	Chromobox protein homolog 3 OS=Homo sapiens GN=CBX3 PE=1 SV=4
695	sp|P01717|LV403_HUMAN	Ig lambda chain V-IV region Hil OS=Homo sapiens PE=1 SV=1
696	sp|P23396|RS3_HUMAN	40S ribosomal protein S3 OS=Homo sapiens GN=RPS3 PE=1 SV=2
697	sp|P17252|KPCA_HUMAN	Protein kinase C alpha type OS=Homo sapiens GN=PRKCA PE=1 SV=3
698	sp|P10070|GLI2_HUMAN	Zinc finger protein GLI2 OS=Homo sapiens GN=GLI2 PE=1 SV=4
699	sp|Q13148|TADBP_HUMAN	TAR DNA-binding protein 43 OS=Homo sapiens GN=TARDBP PE=1 SV=1
700	sp|Q9Y2J0|RP3A_HUMAN	Rabphilin-3A OS=Homo sapiens GN=RPH3A PE=1 SV=1
701	sp|P45381|ACY2_HUMAN	Aspartoacylase OS=Homo sapiens GN=ASPA PE=1 SV=1
702	sp|Q9H8H3|MET7A_HUMAN	Methyltransferase-like protein 7A OS=Homo sapiens GN=METTL7A PE=1 SV=1
703	sp|Q01995|TAGL_HUMAN	Transgelin OS=Homo sapiens GN=TAGLN PE=1 SV=4
704	sp|P07951-2|TPM2_HUMAN	Isoform Epithelial TMe1 of Tropomyosin beta chain OS=Homo sapiens GN=TPM2
705	sp|Q96AE4-2|FUBP1_HUMAN	Isoform 2 of Far upstream element-binding protein 1 OS=Homo sapiens GN=FUBP1
706	sp|Q14974|IMB1_HUMAN	Importin subunit beta-1 OS=Homo sapiens GN=KPNB1 PE=1 SV=2
707	sp|P35908|K22E_HUMAN	Keratin, type II cytoskeletal 2 epidermal OS=Homo sapiens GN=KRT2 PE=1 SV=2
708	sp|Q13232|NDK3_HUMAN	Nucleoside diphosphate kinase 3 OS=Homo sapiens GN=NME3 PE=1 SV=2
709	sp|P48147|PPCE_HUMAN	Prolyl endopeptidase OS=Homo sapiens GN=PREP PE=1 SV=2
710	sp|Q92688|AN32B_HUMAN	Acidic leucine-rich nuclear phosphoprotein 32 family member B OS=Homo sapiens GN=ANP32
711	sp|Q99961|SH3G1_HUMAN	Endophilin-A2 OS=Homo sapiens GN=SH3GL1 PE=1 SV=1
712	sp|P54687|BCAT1_HUMAN	Branched-chain-amino-acid aminotransferase, cytosolic OS=Homo sapiens GN=BCAT1 PE=1
713	sp|Q07666|KHDR1_HUMAN	KH domain-containing, RNA-binding, signal transduction-associated protein 1 OS=Homo sapie
714	sp|P02689|MYP2_HUMAN	Myelin P2 protein OS=Homo sapiens GN=PMP2 PE=1 SV=3
715	sp|P54727|RD23B_HUMAN	UV excision repair protein RAD23 homolog B OS=Homo sapiens GN=RAD23B PE=1 SV=1
716	sp|Q6UX73|CP089_HUMAN	Uncharacterized protein C16orf89 OS=Homo sapiens GN=C16orf89 PE=2 SV=2
717	sp|O95861|BPNT1_HUMAN	3'(2'),5'-bisphosphate nucleotidase 1 OS=Homo sapiens GN=BPNT1 PE=1 SV=1
718	sp|P11940|PABP1_HUMAN	Polyadenylate-binding protein 1 OS=Homo sapiens GN=PABPC1 PE=1 SV=2

Master N	Accession	Name
717	sp|O95861|BPNT1_HUMAN	3'(2'),5'-bisphosphate nucleotidase 1 OS=Homo sapiens GN=BPNT1 PE=1 SV=1
718	sp|P11940|PABP1_HUMAN	Polyadenylate-binding protein 1 OS=Homo sapiens GN=PABPC1 PE=1 SV=2
719	sp|Q9UNS2|CSN3_HUMAN	COP9 signalosome complex subunit 3 OS=Homo sapiens GN=COPS3 PE=1 SV=3
720	sp|O95670|VATG2_HUMAN	V-type proton ATPase subunit G 2 OS=Homo sapiens GN=ATP6V1G2 PE=1 SV=1
721	sp|P35542|SAA4_HUMAN	Serum amyloid A-4 protein OS=Homo sapiens GN=SAA4 PE=1 SV=2
722	sp|P08311|CATG_HUMAN	Cathepsin G OS=Homo sapiens GN=CTSG PE=1 SV=2
723	sp|A0M8Q6|LAC7_HUMAN	Ig lambda-7 chain C region OS=Homo sapiens GN=IGLC7 PE=1 SV=2
724	sp|Q06124-2|PTN11_HUMAN	Isoform PTP2C of Tyrosine-protein phosphatase non-receptor type 11 OS=Homo sapiens GN=
725	sp|P12694|ODBA_HUMAN	2-oxoisovalerate dehydrogenase subunit alpha, mitochondrial OS=Homo sapiens GN=BCKDHA
726	sp|O75348|VATG1_HUMAN	V-type proton ATPase subunit G 1 OS=Homo sapiens GN=ATP6V1G1 PE=1 SV=3
727	sp|O60518|RNBP6_HUMAN	Ran-binding protein 6 OS=Homo sapiens GN=RANBP6 PE=1 SV=2
728	sp|O43251-8|RBM9_HUMAN	Isoform 8 of RNA-binding protein 9 OS=Homo sapiens GN=RBM9
729	sp|O75607|NPM3_HUMAN	Nucleoplasmin-3 OS=Homo sapiens GN=NPM3 PE=1 SV=3
730	sp|Q6ZS82|R9BP_HUMAN	Regulator of G-protein signaling 9-binding protein OS=Homo sapiens GN=RGS9BP PE=2 SV=
731	sp|Q9NSD9|SYFB_HUMAN	Phenylalanyl-tRNA synthetase beta chain OS=Homo sapiens GN=FARSB PE=1 SV=3
732	sp|Q8N3V7-3|SYNPO_HUMAN	Isoform 3 of Synaptopodin OS=Homo sapiens GN=SYNPO
733	sp|P04156|PRIO_HUMAN	Major prion protein OS=Homo sapiens GN=PRNP PE=1 SV=1
734	sp|P31323|KAP3_HUMAN	cAMP-dependent protein kinase type II-beta regulatory subunit OS=Homo sapiens GN=PRKAR
735	sp|P08F94|PKHD1_HUMAN	Fibrocystin OS=Homo sapiens GN=PKHD1 PE=1 SV=1
736	sp|Q96FC7|PHIPL_HUMAN	Phytanoyl-CoA hydroxylase-interacting protein-like OS=Homo sapiens GN=PHYHIPL PE=2 SV=
737	sp|Q5JSL3|DOC11_HUMAN	Dedicator of cytokinesis protein 11 OS=Homo sapiens GN=DOCK11 PE=1 SV=2
738	sp|O60506|HNRPQ_HUMAN	Heterogeneous nuclear ribonucleoprotein Q OS=Homo sapiens GN=SYNCRIP PE=1 SV=2
739	sp|P13591-4|NCAM1_HUMAN	Isoform 4 of Neural cell adhesion molecule 1 OS=Homo sapiens GN=NCAM1
740	sp|P48444|COPD_HUMAN	Coatomer subunit delta OS=Homo sapiens GN=ARCN1 PE=1 SV=1
741	sp|Q9BQI5|SGIP1_HUMAN	SH3-containing GRB2-like protein 3-interacting protein 1 OS=Homo sapiens GN=SGIP1 PE=1
742	sp|Q08495|DEMA_HUMAN	Dematin OS=Homo sapiens GN=EPB49 PE=1 SV=3
743	sp|P13611-4|CSPG2_HUMAN	Isoform V3 of Versican core protein OS=Homo sapiens GN=VCAN
744	sp|Q96L93-4|KI16B_HUMAN	Isoform 3 of Kinesin-like protein KIF16B OS=Homo sapiens GN=KIF16B
745	sp|Q1KMD3|HNRL2_HUMAN	Heterogeneous nuclear ribonucleoprotein U-like protein 2 OS=Homo sapiens GN=HNRNPUL2
746	sp|Q16891|IMMT_HUMAN	Mitochondrial inner membrane protein OS=Homo sapiens GN=IMMT PE=1 SV=1
747	sp|P51858|HDGF_HUMAN	Hepatoma-derived growth factor OS=Homo sapiens GN=HDGF PE=1 SV=1
748	sp|O00429-6|DNM1L_HUMAN	Isoform 6 of Dynamin-1-like protein OS=Homo sapiens GN=DNM1L
749	sp|P21926|CD9_HUMAN	CD9 antigen OS=Homo sapiens GN=CD9 PE=1 SV=4
750	sp|Q15907|RB11B_HUMAN	Ras-related protein Rab-11B OS=Homo sapiens GN=RAB11B PE=1 SV=4
751	sp|P80297|MT1X_HUMAN	Metallothionein-1X OS=Homo sapiens GN=MT1X PE=1 SV=1
752	sp|Q13162|PRDX4_HUMAN	Peroxiredoxin-4 OS=Homo sapiens GN=PRDX4 PE=1 SV=1
753	sp|P39019|RS19_HUMAN	40S ribosomal protein S19 OS=Homo sapiens GN=RPS19 PE=1 SV=2
754	RRRRRsp|Q8N9M1|CS047_HUM	REVERSED Uncharacterized protein C19orf47 OS=Homo sapiens GN=C19orf47 PE=1 SV=1
755	sp|P09486|SPRC_HUMAN	SPARC OS=Homo sapiens GN=SPARC PE=1 SV=1
756	sp|Q7Z2K8-2|GRIN1_HUMAN	Isoform 2 of G protein-regulated inducer of neurite outgrowth 1 OS=Homo sapiens GN=GPRIN
757	sp|P62879|GBB2_HUMAN	Guanine nucleotide-binding protein G(I)/G(S)/G(T) subunit beta-2 OS=Homo sapiens GN=GNB
758	sp|Q9UBT2|SAE2_HUMAN	SUMO-activating enzyme subunit 2 OS=Homo sapiens GN=UBA2 PE=1 SV=2

Table S2: Complete results showing protein identifications and ratios.

	iTRAQ Set 1				iTRAQ Set 2				iTRAQ Set 3			
Master N		NB1: NB2 (114:116)	GBM1: NB2 (115:116)	GBM2: NB2 (117:116)		GBM4: NB3 (114:115)	GBM3: NB3 (116:115)	NB4: NB3 (117:115)		NB5: NB6 (114:116)	GBM5: NB6 (115:116)	GBM6: NB6 (117:116)
1		1.00	1.07	1.08		1.08	1.10	0.99		0.81	0.95	0.89
2		0.98	1.05	1.05		1.02	1.00	1.20		0.81	0.99	0.90
3		0.98	1.15	1.13		1.77	2.23	7.45		0.71	0.96	0.86
4		1.12	23.12	7.24		1.22	1.24	0.99		1.00	1.45	1.29
5		1.66	0.86	0.49		0.22	0.17	1.16		0.80	0.79	0.64
6		0.98	1.22	1.06		1.34	1.26	0.99		1.00	1.10	1.24
7		1.54	2.13	5.55		5.30	4.92	0.50		0.03	0.65	0.46
8		1.46	7.80	7.24		3.31	5.20	1.37		0.33	7.66	7.87
9		1.42	1.00	0.50		0.21	0.47	0.94		1.03	0.86	0.82
10		2.73	4.70	12.47		10.19	10.67	0.59		0.03	0.82	0.33
11		1.07	1.67	0.96		0.04	0.05	0.13		1.64	0.80	0.79
12		1.47	1.02	0.66		0.90	0.56	1.56		1.66	0.69	3.19
13		0.56	1.36	0.85						6.67	5.86	11.27
14		0.98	0.92	0.90		0.05	0.07	0.63		1.33	0.08	0.08
15		1.00	0.34	1.01		0.78	0.84	0.18		5.55	2.88	2.09
16		1.18	0.53	0.43		0.37	0.36	0.82		2.27	0.15	0.43
17		1.24	0.21	0.29		0.22	0.30	0.64		0.52	0.17	0.29
18		0.65	0.17	0.22		0.08	0.13	0.77		7.59	1.77	2.68
19		1.49	2.13	3.50		2.96	2.75	0.76		0.06	1.28	0.54
20		0.80	0.35	0.69		0.90	0.64	0.61		3.44	3.05	7.18
21						0.10	0.03	0.70		1.39	0.04	0.30
22		1.01	1.05	1.20		3.77	4.49	1.07		0.03	0.88	0.88
23		1.54	0.33	2.38		0.11	0.17	0.43		1.85	2.49	4.49
24		0.78	0.17	0.25		0.51	0.50	0.63		1.09	0.61	0.30
25		0.92	1.00	0.90		1.07	1.16	1.10		0.18	1.94	3.84
26		1.34	0.36	0.11		0.06	0.05	0.67		0.77	0.19	0.31
27		0.92	0.96	2.96		0.24	0.15	0.37		0.55	0.24	0.17
28		1.45	0.79	0.30		1.03	1.05	0.97		3.08	1.74	0.53
29		1.29	4.74	2.03		2.54	2.65	1.04		1.51	4.70	12.36
30		0.17	0.89	3.87		0.56	0.61	0.61		0.03	1.17	1.00
31		0.52	0.12	0.02		0.05	0.10	1.38		0.08	0.04	0.07
32		1.10	2.03	0.26		0.09	0.07	0.28		1.32	0.07	0.03
33		0.69	0.98	0.70		1.16	0.74	2.86		1.12	0.43	0.39
34		0.89	0.21	0.26		0.03	0.04	0.86		5.65	0.15	0.85
35		1.32	0.39	0.45		0.05	0.09	0.60		1.56	0.02	0.03
36		1.04	0.14	0.03		0.05	0.03	0.81				
37		0.69	1.39	2.63		4.49	3.50	1.75		1.01	1.06	0.54
38		1.10	1.18	0.35		0.51	0.61	0.34		0.30	0.73	0.16
39		0.79	1.39	1.77		9.91	10.76	1.08		0.03	0.48	0.58
40		2.36	6.03	15.56		7.59	8.79	1.20		0.03	0.37	0.29
41		0.44	0.38	0.20		0.34	0.59	1.69		0.81	0.82	0.90
42		1.49	6.25	2.70		1.08	1.17	1.87		0.05	0.90	0.58
43		4.49	2.91	3.19		6.67	5.20	0.84		8.87	21.48	11.48
44		2.27	4.92	1.66		1.91	2.81	0.81		2.54	1.58	1.03
45		2.00	2.19	3.47		0.72	0.61	0.38		1.33	0.92	1.56
46		0.31	0.87	0.61		0.49	0.25	0.97				

	iTRAQ Set 1				iTRAQ Set 2				iTRAQ Set 3			
Master N		NB1: NB2 (114:116)	GBM1: NB2 (115:116)	GBM2: NB2 (117:116)		GBM4: NB3 (114:115)	GBM3: NB3 (116:115)	NB4: NB3 (117:115)		NB5: NB6 (114:116)	GBM5: NB6 (115:116)	GBM6: NB6 (117:116)
47		0.83	0.23	0.44		0.05	0.08	0.67		1.08	0.14	0.14
48		0.24	0.65	0.67		0.36	0.17	1.01				
49		1.06	3.47	1.61		3.31	3.63	0.86		1.96	30.48	28.31
50		1.47	0.16	0.85		0.56	0.28	0.49		2.81	1.24	0.71
51		1.05	2.70	2.17		11.59	9.64	0.42		2.78	8.47	6.14
52		1.42	2.23	8.17		1.13	1.15	0.48		2.23	1.39	5.06
53		0.35	0.31	0.77		0.69	0.67	1.72		0.76	0.50	0.04
54		0.91	1.11	0.97						0.05	1.41	0.95
55		0.96	0.63	0.58		0.51	0.51	0.96		2.36	0.83	1.92
56		0.86	0.59	0.41		0.33	0.36	0.58		2.03	0.98	0.59
57		0.82	0.47	1.03		0.54	0.45	0.38		4.25	0.64	1.27
58		0.96	0.38	0.52		0.10	0.16	0.72		2.36	0.19	0.23
59		0.90	0.52	0.42		0.21	0.29	0.83				
60		0.81	0.81	0.50		1.05	2.27	0.74		0.95	3.50	4.13
61		1.07	0.37	0.30		0.04	0.05	0.54		1.64	0.13	0.13
62						10.19	10.96	1.71		0.51	12.13	5.70
63		0.77	0.29	0.69		0.36	0.47	0.41		1.56	0.46	0.16
64		1.26	0.62	1.33		0.41	0.66	0.83		2.51	2.33	4.25
65		1.37	0.86	0.98		0.86	0.86	0.90		0.92	0.96	0.90
66						1.71	3.34	1.03		0.27	2.94	1.91
67		0.82	0.79	0.70		0.21	0.18	1.01		0.33	0.93	0.13
68		0.75	0.90	0.92		4.61	4.45	0.89		0.03	1.18	0.83
69		1.01	1.16	1.22		0.81	0.70	0.60		1.28	1.02	0.93
70		1.56	1.98	3.02		1.38	1.32	0.93		13.80	3.80	11.17
71						13.55	11.59	0.71		0.82	8.87	4.79
72		4.79	18.20	20.32		9.64	11.38	1.21		1.24	17.22	8.47
73		1.06	0.77	0.22		0.11	0.12	1.32				
74		1.22	0.54	0.15		0.07	0.10	0.59		3.31	0.52	0.25
75		0.75	0.62	0.29		1.47	1.43	0.52		3.70	4.49	8.02
76		7.38	12.36	0.79		0.74	0.70	0.89		4.21	0.37	0.02
77		0.95	1.02	1.02		1.07	1.16	0.82		0.27	1.56	0.89
78		0.85	0.84	1.80		1.09	1.11	0.92		1.56	1.10	2.33
79		0.92	0.72	0.61		0.69	0.77	0.47		2.63	0.52	0.55
80		0.91	0.66	0.88		1.05	1.06	0.99		0.39	1.57	0.98
81		0.79	2.70	0.67		7.45	5.97	0.33		9.91	24.43	12.02
82		1.53	0.82	0.83		0.58	0.41	0.88		2.75	0.77	1.11
83		1.21	1.92	1.50		9.91	9.04	2.68		0.50	5.50	3.91
84		1.20	0.96	1.00		0.63	0.85	0.90		1.45	0.69	1.41
85		1.07	0.53	0.18		0.13	0.25	0.70		1.46	0.53	0.38
86		1.71	2.42	2.11		16.29	13.18	3.10		1.32	9.82	5.70
87						7.66	12.82	0.51				
88						6.08	8.17	5.70		0.14	2.27	0.58
89		1.20	0.36	0.15		0.91	0.98	0.99		2.81	8.47	4.74
90		0.68	0.16	0.07		0.04	0.05	0.44		1.50	0.10	0.07
91		0.77	0.74	1.98		1.31	1.26	0.89		1.72	2.49	5.40
92		1.80	1.71	4.57		0.20	0.15	0.66		2.42	0.85	1.03
93		1.01	0.90	1.03		3.25	2.49	1.64				
94		0.68	0.44	0.70		0.21	0.14	0.95				

	iTRAQ Set 1				iTRAQ Set 2				iTRAQ Set 3			
**Master N**		**NB1: NB2 (114:116)**	**GBM1: NB2 (115:116)**	**GBM2: NB2 (117:116)**		**GBM4: NB3 (114:115)**	**GBM3: NB3 (116:115)**	**NB4: NB3 (117:115)**		**NB5: NB6 (114:116)**	**GBM5: NB6 (115:116)**	**GBM6: NB6 (117:116)**
95		1.63	0.40	1.19		0.52	0.44	0.68		2.25	0.64	0.73
96		1.36	3.47	1.84		2.86	3.05	1.80		9.29	5.01	10.96
97						20.32	17.70	3.28		0.03	0.18	0.18
98		0.25	0.22	0.18		0.48	0.40	1.34				
99		0.85	0.95	0.53		0.24	0.20	0.76		2.29	1.27	0.76
100		0.95	0.95	0.79		0.09	0.13	0.64		2.99	0.38	0.42
101		0.98	1.12	1.32		1.07	0.65	0.98		1.31	0.60	0.72
102		1.05	1.20	0.98		0.10	0.71	1.31		3.98	0.12	0.75
103		0.71	0.52	0.16		0.14	0.28	1.74		2.19	0.70	0.74
104		1.29	1.91	2.07		2.61	2.51	0.23		1.10	1.92	1.57
105		0.35	1.25	3.77		1.94	5.60	0.90		0.02	1.12	0.79
106		1.13	0.17	0.37		0.64	0.74	1.05		2.27	0.18	0.65
107		0.54	1.26	1.92		3.77	2.38	0.90		1.04	1.00	0.44
108		0.95	1.22	0.97		0.95	0.70	0.78		1.66	0.69	0.27
109		1.38	1.06	0.66						2.78	0.96	0.90
110						8.79	8.24	1.31		0.15	1.32	2.94
111		0.87	0.79	0.92		1.92	1.67	0.82		0.68	4.13	2.09
112		1.31	0.15	0.04		0.18	0.25	0.85		2.42	0.34	0.06
113		0.58	2.65	0.77		33.11	16.00	1.56		0.19	0.64	0.51
114		1.07	0.30	0.24		0.19	0.22	0.42		2.25	0.07	0.03
115		0.81	0.35	0.24		0.14	0.09	1.28		0.89	0.16	0.11
116		1.47	1.53	2.42		1.03	0.74	0.72		4.57	1.79	2.40
117		0.85	0.79	0.81		0.47	1.05	1.98		0.70	0.69	0.47
118		2.33	0.58	0.82		0.24	0.18	0.64		2.38	0.70	0.95
119		0.84	0.60	0.47		0.05	0.19	0.98				
120						8.55	7.38	0.69		0.80	12.82	4.61
121		1.10	0.25	0.21		0.44	0.46	1.07		0.65	0.83	0.39
122		1.63	0.66	1.84		0.31	0.54	0.66		1.69	0.72	0.90
123										0.60	0.76	0.94
124		0.52	0.42	0.69		0.55	0.40	1.72				
125		1.33	0.79	0.43		0.82	0.79	1.03		0.91	0.58	0.70
126		1.05	0.26	0.37		0.72	0.76	0.84		0.95	0.92	0.69
127		1.00	0.91	0.70		0.58	0.60	1.00		1.84	0.48	0.70
128		0.95	0.67	0.26		0.09	0.08	0.68		1.87	0.27	0.07
129		0.70	2.81	6.37		14.32	14.86	1.03		0.27	1.09	0.56
130		0.75	1.24	1.75								
131		0.97	1.02	0.78		0.61	0.47	1.38				
132		0.92	1.01	1.03		1.85	2.07	1.21		1.89	0.94	0.95
133		1.80	0.54	0.08		0.95	0.97	0.65		2.75	0.21	0.21
134		0.96	9.12	3.84		4.06	2.27	0.67				
135		1.31	0.38	0.23		0.42	0.14	1.26				
136		1.09	1.21	1.66		11.59	9.38	0.95		0.02	0.70	0.77
137		1.19	0.50	0.22		0.13	0.30	2.00				
138		1.02	1.20	1.03		0.86	0.90	0.83		1.21	0.91	0.81
139		1.03	0.80	0.88		0.59	0.65	0.82		1.51	0.19	0.08
140		0.83	0.33	0.11		0.24	0.29	0.76				
141		1.31	5.40	9.38		7.24	11.69	0.50		0.02	0.81	0.82
142						2.01	2.07	0.66		2.94	2.78	4.66

	iTRAQ Set 1				iTRAQ Set 2				iTRAQ Set 3			
**Master N**		**NB1: NB2 (114:116)**	**GBM1: NB2 (115:116)**	**GBM2: NB2 (117:116)**		**GBM4: NB3 (114:115)**	**GBM3: NB3 (116:115)**	**NB4: NB3 (117:115)**		**NB5: NB6 (114:116)**	**GBM5: NB6 (115:116)**	**GBM6: NB6 (117:116)**
143		0.90	1.39	0.95		0.86	0.74	0.71		3.87	0.17	0.09
144		1.02	0.51	0.13		0.54	0.39	0.64		3.05	0.25	0.14
145		0.91	1.41	1.58						0.07	0.74	0.50
146		0.84	0.53	0.50		0.37	0.29	1.08				
147		0.97	0.83	1.05		0.85	0.98	1.01		0.98	0.97	0.87
148		1.29	1.85	2.61		1.11	0.98	1.01				
149		0.96	1.61	3.70		0.95	0.77	0.27		7.94	2.81	1.18
150		1.42	0.88	2.15		1.16	1.11	1.00		0.91	1.26	1.49
151		1.31	1.26	2.13		0.94	0.90	1.02				
152										0.24	2.54	0.64
153		1.32	0.76	0.59		0.11	0.18	0.55		0.94	0.31	0.35
154		0.88	2.91	2.31		0.99	1.13	0.86		1.75	1.12	1.17
155		1.15	0.55	0.71		0.31	0.44	1.00				
156		0.97	0.93	0.89		1.16	1.51	0.85				
157		1.08	1.27	0.51		7.94	6.92	1.25		1.01	1.00	0.78
158		1.03	0.93	0.85		0.60	0.65	0.81		0.72	0.49	1.25
159		0.82	0.72	1.01		1.20	1.18	0.58		0.16	0.72	0.39
160		1.07	0.41	0.30		0.33	0.19	0.27		0.67	0.61	0.53
161		1.51	5.40	3.87		1.24	1.63	12.13		0.02	1.43	0.76
162		2.83	1.66	0.48		0.10	0.21	0.38		0.58	0.12	0.04
163						0.21	0.19	0.96		1.45	1.11	0.96
164		2.29	4.49	15.85		14.06	15.70	2.81		0.03	0.56	0.30
165										0.08	0.82	0.49
166						19.77	40.18	2.09		0.03	4.09	0.90
167		0.96	0.97	0.86		0.14	0.20	1.53				
168						3.13	2.65	0.76		0.30	1.43	3.13
169		1.00	0.99	0.86		0.79	0.95	0.79		1.14	0.62	0.77
170		0.86	0.52	0.47		1.89	1.50	1.63		0.04	2.25	3.44
171						3.70	3.47	0.67		0.35	1.46	0.62
172						5.60	6.08	0.90		3.19	5.15	4.57
173						11.27	23.33	0.61		0.04	7.73	1.26
174										2.17	2.17	2.15
175		1.10	0.99	1.13		1.01	1.06	1.08		0.58	1.00	1.38
176		0.95	2.96	2.78		2.47	6.19	0.96		1.45	1.80	2.33
177		0.46	0.54	0.23		0.64	0.65	1.07				
178		1.09	0.70	0.69		0.31	0.76	1.28				
179		0.68	3.22	8.79		9.91	10.38	1.60		0.03	0.47	0.52
180		0.89	0.83	0.44		0.24	0.49	1.47				
181										2.68	3.47	3.87
182		1.28	1.25	1.00		0.93	1.02	1.00		2.44	0.60	0.65
183		0.92	0.99	0.85		0.48	0.47	0.71		1.02	1.04	1.03
184						5.11	5.35	0.46		1.43	1.64	1.45
185										0.86	0.84	0.90
186		1.67	2.00	4.29		1.00	1.67	0.61		1.01	3.16	2.17
187						17.06	13.43	0.45		0.52	3.28	0.95
188		1.45	1.53	2.17						0.05	1.13	0.66
189		1.49	1.05	0.21		1.02	0.95	0.92		1.72	1.05	1.37
190		0.58	0.46	0.34		0.12	0.16	0.60				
191										0.05	0.90	0.51
192		11.38	2.42	6.43		1.08	1.15	0.82		1.34	1.04	1.02

	iTRAQ Set 1				iTRAQ Set 2				iTRAQ Set 3			
**Master N**		**NB1: NB2 (114:116)**	**GBM1: NB2 (115:116)**	**GBM2: NB2 (117:116)**		**GBM4: NB3 (114:115)**	**GBM3: NB3 (116:115)**	**NB4: NB3 (117:115)**		**NB5: NB6 (114:116)**	**GBM5: NB6 (115:116)**	**GBM6: NB6 (117:116)**
193						0.87	0.64	0.53		0.94	0.85	0.85
194		0.82	0.93	1.02		1.00	0.28	0.86		1.31	0.90	0.69
195		1.04	1.03	0.92								
196		0.90	0.59	0.53		1.69	1.57	1.14				
197		0.90	1.10	1.46		1.17	1.32	0.73				
198										0.08	0.95	1.06
199		0.92	1.53	0.92		1.09	1.22	0.90		1.56	5.86	3.47
200										0.11	0.69	0.46
201						0.19	0.26	0.64				
202		1.37	1.19	0.99		0.76	0.77	0.91				
203		1.36	1.11	1.12						1.19	0.66	0.29
204										0.02	0.72	0.52
205		1.18	0.78	1.12		1.38	0.79	0.74		3.08	0.21	2.29
206		0.77	0.60	0.53		0.60	0.46	0.75		2.88	0.42	0.53
207						0.90	0.93	0.98				
208		0.86	0.18	0.09		0.04	0.02	0.50		1.18	0.07	0.06
209		0.87	0.90	0.77		0.86	1.36	1.11				
210						23.55	16.75	8.32		1.04	2.51	1.15
211		0.80	0.15	0.21		0.58	0.52	0.70				
212		1.80	1.06	0.22		0.41	0.26	0.66		3.22	0.09	0.56
213						2.33	3.66	1.45				
214		5.65	7.87	5.40		1.98	3.56	0.56				
215		0.59	0.38	0.07		1.39	1.96	3.40		1.15	0.09	0.12
216						0.91	0.74	1.63		0.83	3.80	2.81
217		0.95	0.35	0.10		0.09	0.11	0.79		0.21	0.30	0.32
218		1.20	0.82	1.12		0.21	0.20	0.71		2.96	0.48	0.72
219		0.98	1.00	1.21		0.06	0.09	0.74		0.58	0.58	0.45
220						7.18	7.18	1.82				
221		0.96	1.27	3.84		1.51	1.87	0.87		0.06	0.36	0.21
222		1.09	0.82	1.10						3.28	0.48	1.39
223		1.18	0.94	0.80		0.48	0.77	1.64				
224		0.96	1.13	1.14		0.54	0.40	0.76		0.94	0.55	0.48
225		0.51	0.52	0.56		1.45	0.73	0.83		1.03	0.69	0.45
226		1.61	2.91	4.53		1.21	1.14	0.99				
227		0.72	1.03	0.49		2.25	2.54	1.01		0.96	0.25	0.36
228		1.36	0.50	0.74		0.90	0.81	1.06		2.31	0.73	0.77
229						0.50	0.90	0.77				
230						2.13	1.85	0.27				
231		1.14	1.36	1.17						0.95	0.53	0.81
232		7.18	14.59	23.55						0.45	1.00	1.32
233		1.22	2.00	2.54		0.42	0.99	0.59		1.45	0.84	1.96
234		0.30	0.72	0.46		3.63	5.25	1.57		0.86	3.98	2.33
235		0.90	1.10	1.13		0.06	0.08	0.35		2.78	0.37	0.65
236		1.37	0.57	0.45		0.50	0.37	0.81		1.79	0.49	0.58
237		1.05	0.93	1.21						1.05	2.19	2.51
238		4.45	14.32	7.11		0.78	0.68	1.10		0.23	0.58	0.60

	iTRAQ Set 1				iTRAQ Set 2				iTRAQ Set 3			
**Master N**		**NB1: NB2 (114:116)**	**GBM1: NB2 (115:116)**	**GBM2: NB2 (117:116)**		**GBM4: NB3 (114:115)**	**GBM3: NB3 (116:115)**	**NB4: NB3 (117:115)**		**NB5: NB6 (114:116)**	**GBM5: NB6 (115:116)**	**GBM6: NB6 (117:116)**
239						0.52	0.36	0.88		1.16	0.97	0.81
240						1.06	1.21	0.70				
241		1.27	2.86	1.67		1.79	2.51	0.27				
242						1.98	2.11	0.54		6.98	8.79	9.55
243						0.90	0.75	0.97		1.38	3.28	4.83
244						0.93	1.24	0.79				
245						0.95	0.93	1.37		0.98	0.96	0.96
246						0.70	0.85	0.99		1.47	1.22	0.74
247		0.61	0.95	2.27		0.93	0.71	0.99				
248		1.13	1.18	1.36								
249		3.47	2.70	7.11						2.40	0.97	0.97
250		1.16	1.07	1.02		0.53	0.70	0.90		1.43	0.82	0.67
251						0.09	0.15	0.90				
252		1.67	0.90	0.94						1.61	0.73	0.27
253		1.02	0.72	0.68		0.18	0.21	0.60				
254						0.42	0.33	0.19		2.17	1.39	1.45
255						1.05	1.06	0.74		0.79	1.07	0.90
256										0.77	0.89	1.38
257		0.83	0.88	0.70		1.14	1.20	0.87		0.92	1.10	0.99
258						0.99	0.96	0.94		0.16	1.51	1.12
259		0.97	0.86	0.70								
260		0.99	0.70	1.03						1.34	1.09	1.28
261		1.08	0.58	0.17		0.51	0.80	1.04				
262		1.13	0.84	0.77								
263						1.16	1.13	0.81				
264		0.72	0.39	0.38		0.23	0.24	0.87				
265												
266						0.41	0.58	1.18				
267		0.61	0.94	0.83		0.95	0.90	0.84				
268						12.02	9.73	0.95		1.07	2.61	1.75
269										0.07	2.27	0.16
270										0.93	0.82	2.38
271						0.56	0.33	0.86				
272		1.60	0.86	8.17								
273		1.69	0.91	0.66						1.32	0.46	0.33
274		0.96	0.86	1.32		0.54	0.75	0.69		1.25	1.21	3.08
275		1.12	1.16	1.10								
276						0.82	1.47	0.27		1.82	0.75	0.21
277		1.02	1.27	0.92		2.38	2.31	1.33		1.08	0.95	1.09
278		1.06	1.00	1.04								
279		0.95	0.97	1.32						0.86	1.24	1.46
280		0.91	1.01	1.13								
281						0.34	0.07	0.90				
282		0.90	0.18	0.12		0.62	0.70	0.87				
283						0.33	0.32	0.67		1.49	0.79	0.53
284										1.92	1.39	0.10
285		0.78	0.24	0.15		0.17	0.05	0.40				
286		0.93	0.39	0.64		0.20	0.20	1.09		1.22	0.22	3.02

	iTRAQ Set 1				iTRAQ Set 2				iTRAQ Set 3			
**Master N**		**NB1: NB2 (114:116)**	**GBM1: NB2 (115:116)**	**GBM2: NB2 (117:116)**		**GBM4: NB3 (114:115)**	**GBM3: NB3 (116:115)**	**NB4: NB3 (117:115)**		**NB5: NB6 (114:116)**	**GBM5: NB6 (115:116)**	**GBM6: NB6 (117:116)**
287		1.08	0.45	0.17		0.07	0.15	0.23				
288										0.01	0.56	0.29
289										0.30	1.04	0.72
290		0.45	0.16	1.67						0.79	2.25	3.19
291		1.42	0.93	1.32								
292										1.14	1.04	0.96
293		1.06	0.67	0.98		1.15	1.19	0.79		0.70	1.04	1.09
294										0.09	0.92	0.54
295		0.13	0.66	0.65		2.65	3.08	1.41		2.88	22.49	6.98
296		1.00	0.82	1.03								
297		1.11	1.85	1.58		0.73	0.69	1.45		0.94	1.25	1.09
298						14.19	13.55	0.53				
299		1.26	1.98	2.11						0.12	0.86	1.61
300						25.35	30.48	5.97		1.85	10.38	4.45
301												
302		0.65	0.31	0.06		0.80	1.09	0.98				
303						1.14	1.09	1.18				
304						2.58	1.80	1.29		0.93	0.97	0.95
305		4.97	9.20	0.26		0.85	0.69	0.73		3.22	0.24	0.03
306						1.00	0.82	0.72		1.38	0.98	1.08
307		1.04	0.52	0.46		0.70	0.60	0.82		1.00	0.53	0.44
308		1.14	0.48	0.73		0.37	0.37	0.65				
309		1.47	0.63	1.06		1.12	1.04	1.22				
310						0.14	0.35	0.95				
311						2.21	2.19	0.72		1.01	1.26	1.15
312		1.16	0.94	1.12						1.82	0.77	0.99
313												
314						1.05	1.28	1.09		1.26	1.04	1.08
315		3.05	9.55	2.58		0.91	0.66	1.06				
316						0.29	0.29	0.65		1.77	0.88	1.27
317						0.95	1.14	1.74				
318						0.48	0.68	1.07				
319		0.67	0.46	0.94		1.51	1.17	3.98				
320		0.95	0.68	0.71						1.19	1.06	1.02
321		1.25	0.52	0.59								
322		0.26	0.69	1.63		5.35	6.67	3.28		0.72	1.25	0.91
323										0.63	1.26	1.85
324						0.33	0.29	0.77				
325		1.22	0.81	1.05		1.32	1.47	1.37		0.22	0.99	0.64
326		1.06	0.87	1.08						0.66	0.83	1.03
327		1.14	0.81	0.96						1.19	1.09	1.04
328		1.01	1.26	1.09		1.19	0.86	0.77				
329						1.69	1.37	1.32		0.93	1.56	1.64
330						0.69	0.95	1.01				
331												
332						0.79	0.77	1.33				
333										0.71	0.89	0.95
334		0.94	3.53	4.06		9.73	10.86	1.04		2.03	6.03	10.47

	iTRAQ Set 1				iTRAQ Set 2				iTRAQ Set 3			
**Master N**		**NB1: NB2 (114:116)**	**GBM1: NB2 (115:116)**	**GBM2: NB2 (117:116)**		**GBM4: NB3 (114:115)**	**GBM3: NB3 (116:115)**	**NB4: NB3 (117:115)**		**NB5: NB6 (114:116)**	**GBM5: NB6 (115:116)**	**GBM6: NB6 (117:116)**
335		1.67	0.83	0.87		0.96	0.84	1.53				
336												
337		0.95	0.82	0.92								
338		1.20	0.80	0.90						1.45	0.93	0.98
339		0.95	0.70	0.69						0.85	0.50	0.65
340		1.11	1.66	1.77		2.01	2.29	0.95		0.88	0.95	1.45
341		0.33	0.53	0.46		1.16	1.22	1.69				
342										0.22	4.02	0.51
343										0.07	1.75	0.41
344		0.78	0.88	0.27								
345		1.14	1.01	2.94		1.39	1.36	0.76				
346										1.15	0.99	1.47
347						0.45	0.54	0.97		1.89	1.07	1.43
348						0.54	0.47	0.85				
349										1.51	0.79	1.91
350		1.07	1.06	1.36		0.24	0.44	0.32				
351						0.88	0.21	0.98				
352		1.28	2.49	2.86						2.38	1.53	3.25
353						1.63	1.64	0.77		1.00	0.91	0.65
354		0.96	3.73	1.42								
355		1.01	1.04	1.39						1.13	0.67	0.94
356						0.95	1.25	1.41		0.98	1.04	0.99
357		0.99	1.00	1.01								
358		1.08	1.51	0.70								
359		1.56	0.70	0.29								
360		0.91	0.93	1.04						2.36	0.26	0.19
361						0.44	0.69	0.89				
362										0.91	0.89	1.37
363		1.17	0.99	2.58								
364		1.18	2.70	1.80						0.96	0.95	0.93
365		1.29	1.53	1.67		0.85	1.03	0.89				
366						0.53	0.43	0.75				
367		1.12	0.99	0.91								
368						1.91	2.00	0.84		1.53	1.61	1.87
369						0.60	0.66	1.25				
370										0.91	0.86	0.86
371						0.38	0.43	0.50				
372												
373						1.22	1.94	0.86				
374										0.82	0.74	0.69
375		0.90	0.85	1.04								
376						0.80	1.21	0.65				
377										0.94	1.61	0.99
378						1.02	0.98	1.43				
379		2.09	0.98	1.58								
380										1.21	2.42	2.51
381						0.30	0.33	0.93		1.03	1.02	1.03
382												

	iTRAQ Set 1				iTRAQ Set 2				iTRAQ Set 3			
Master N		NB1: NB2 (114:116)	GBM1: NB2 (115:116)	GBM2: NB2 (117:116)		GBM4: NB3 (114:115)	GBM3: NB3 (116:115)	NB4: NB3 (117:115)		NB5: NB6 (114:116)	GBM5: NB6 (115:116)	GBM6: NB6 (117:116)
383		1.08	1.18	0.83								
384		1.42	0.27	0.03		0.37	0.21	0.72		2.31	0.02	0.02
385										0.91	0.86	1.11
386		1.02	1.01	0.86		0.29	0.27	0.59				
387										1.36	11.91	12.02
388										1.77	2.21	0.35
389												
390						0.57	1.03	0.37		1.79	2.36	2.44
391						0.35	1.98	0.15				
392		0.80	0.56	0.85		1.17	1.32	0.73		1.12	0.86	1.92
393		1.01	0.90	0.92						1.67	0.33	0.42
394												
395										0.38	0.75	0.47
396												
397						1.79	1.51	0.77				
398						0.02	0.11	0.86				
399										0.93	1.12	1.16
400										0.58	1.58	0.27
401												
402		1.10	1.03	0.84		1.47	1.42	1.21				
403		0.70	0.94	1.08		1.19	1.43	0.84				
404												
405												
406										1.25	0.22	0.23
407						0.95	1.00	0.56		1.06	0.64	1.02
408		1.72	0.66	1.69								
409						1.38	1.22	0.98				
410		0.97	0.83	0.94						1.67	0.38	0.68
411						42.85	54.95	4.37				
412												
413												
414		0.82	0.32	0.23		0.14	0.03	2.09				
415										1.10	0.86	0.72
416						1.41	1.12	1.03				
417		1.89	0.95	0.45								
418						0.28	0.40	1.28				
419												
420										0.87	0.72	0.69
421		8.71	7.24	13.43						5.75	2.36	5.92
422												
423						0.36	0.34	0.59				
424		0.57	1.28	0.77								
425										3.70	0.82	0.67
426										0.10	0.26	0.34
427										1.38	0.69	0.82
428						1.21	1.37	1.01				
429										5.97	7.59	15.00
430										2.68	0.55	0.84

	iTRAQ Set 1				iTRAQ Set 2				iTRAQ Set 3			
Master N		NB1: NB2 (114:116)	GBM1: NB2 (115:116)	GBM2: NB2 (117:116)		GBM4: NB3 (114:115)	GBM3: NB3 (116:115)	NB4: NB3 (117:115)		NB5: NB6 (114:116)	GBM5: NB6 (115:116)	GBM6: NB6 (117:116)
431						0.73	0.82	1.18				
432		1.14	1.36	1.74						0.87	1.05	0.88
433												
434		0.85	2.15	1.20						1.63	0.81	0.14
435		0.90	0.84	0.71								
436		0.95	0.89	1.09		0.92	0.80	1.11				
437										3.40	2.09	4.92
438						1.19	1.12	0.70				
439		0.55	0.80	0.52		0.94	0.74	0.42				
440												
441												
442		0.96	0.77	0.80								
443		1.06	1.05	1.20						1.33	1.00	0.93
444						0.89	0.87	1.07				
445		1.94	2.00	2.00						1.82	0.05	0.70
446						1.27	1.19	1.01				
447										0.61	1.17	0.60
448		2.42	0.21	0.22		0.02	0.04	0.79				
449										2.47	0.95	0.90
450										0.97	1.09	2.51
451										1.60	1.19	2.49
452										0.28	0.57	0.46
453						1.49	0.81	1.07				
454						0.81	0.89	0.84				
455						0.81	0.64	0.78				
456										0.11	0.33	0.48
457										2.21	0.61	0.73
458										0.37	0.82	0.53
459						0.70	0.78	0.76				
460										2.29	0.21	0.24
461										1.47	1.56	2.13
462						1.28	1.25	0.84				
463						0.89	1.29	0.82				
464		1.12	0.72	1.41								
465										1.09	1.69	2.01
466												
467												
468		0.91	0.59	0.72								
469		0.68	1.14	1.61								
470		0.89	0.97	1.41								
471		0.90	0.52	0.55						1.46	0.61	0.62
472		0.98	0.92	0.95						1.04	0.76	0.94
473										1.04	0.52	0.47
474										0.07	0.95	0.41
475		1.15	1.12	0.97								
476						0.94	0.91	1.00				
477		1.46	1.38	1.72						4.09	6.67	11.07
478		0.91	1.66	1.89								

	iTRAQ Set 1				iTRAQ Set 2				iTRAQ Set 3			
Master N		NB1: NB2 (114:116)	GBM1: NB2 (115:116)	GBM2: NB2 (117:116)		GBM4: NB3 (114:115)	GBM3: NB3 (116:115)	NB4: NB3 (117:115)		NB5: NB6 (114:116)	GBM5: NB6 (115:116)	GBM6: NB6 (117:116)
479												
480						7.24	7.45	0.74		4.83	5.86	8.95
481										1.45	2.03	1.53
482										1.61	1.34	1.75
483												
484												
485						0.45	0.52	0.70				
486										0.79	0.99	1.12
487										0.41	0.76	0.70
488										1.09	1.45	1.19
489						1.58	1.29	1.33				
490						0.90	1.00	0.90				
491												
492		1.05	0.62	0.94								
493						1.54	1.29	1.61				
494												
495												
496										0.98	0.52	0.95
497												
498										1.49	0.65	0.63
499		0.97	1.05	0.97						1.10	1.08	1.45
500												
501						1.11	1.34	0.93				
502												
503		0.97	0.86	0.70		0.88	1.37	0.98				
504		0.77	0.76	0.75						0.96	0.87	0.67
505										0.98	0.93	0.95
506										0.93	1.24	1.19
507												
508												
509		1.01	0.59	0.97								
510						2.19	2.68	0.44				
511		1.31	1.26	0.94								
512						0.63	0.51	0.90				
513						1.94	1.51	1.15				
514												
515		1.27	1.20	1.42								
516		1.05	1.32	1.49								
517												
518												
519										9.73	23.99	20.14
520												
521						0.41	0.37	0.67				
522												
523												
524												
525												
526		0.91	1.01	1.46								

	iTRAQ Set 1				iTRAQ Set 2				iTRAQ Set 3			
Master N		NB1: NB2 (114:116)	GBM1: NB2 (115:116)	GBM2: NB2 (117:116)		GBM4: NB3 (114:115)	GBM3: NB3 (116:115)	NB4: NB3 (117:115)		NB5: NB6 (114:116)	GBM5: NB6 (115:116)	GBM6: NB6 (117:116)
527										1.36	0.84	1.11
528						0.58	0.42	0.67				
529						1.98	2.65	1.15				
530												
531		0.53	0.58	1.14								
532												
533		0.93	1.49	1.26		3.50	3.08	1.84		1.24	3.70	3.70
534										0.98	0.96	0.96
535		0.79	1.01	2.05								
536												
537						0.70	0.67	0.91				
538						1.22	1.05	2.00				
539												
540		0.99	1.27	0.89								
541										2.36	1.71	3.60
542												
543		1.05	1.04	0.88								
544												
545										0.77	0.95	0.95
546						1.53	1.11	1.32				
547						0.64	0.57	1.20				
548						3.98	3.28	1.24				
549												
550						0.09	0.11	0.79				
551		0.71	0.95	2.42								
552												
553		0.97	0.47	0.90								
554												
555		1.10	0.95	0.90								
556												
557												
558						99.08	99.08	99.08				
559												
560												
561						1.03	1.34	1.18				
562		0.87	1.74	7.38								
563												
564						0.82	0.63	0.79				
565		1.85	1.20	2.38								
566												
567		0.80	1.15	1.96								
568		1.34	1.42	1.26		0.82	1.07	0.66				
569		0.99	0.73	0.82								
570												
571		1.03	0.95	1.06								
572												
573										1.58	0.97	0.55
574										0.13	0.53	0.32

	iTRAQ Set 1				iTRAQ Set 2				iTRAQ Set 3			
**Master N**		**NB1: NB2 (114:116)**	**GBM1: NB2 (115:116)**	**GBM2: NB2 (117:116)**		**GBM4: NB3 (114:115)**	**GBM3: NB3 (116:115)**	**NB4: NB3 (117:115)**		**NB5: NB6 (114:116)**	**GBM5: NB6 (115:116)**	**GBM6: NB6 (117:116)**
575		1.02	1.00	3.47								
576						0.59	0.60	1.26				
577						0.44	0.19	1.15				
578		0.58	1.75	1.06								
579												
580		0.95	1.03	0.90								
581												
582												
583		1.79	3.94	3.77						0.16	0.49	2.58
584										2.54	0.54	0.80
585												
586						0.61	0.79	0.99				
587										1.38	1.57	2.25
588										6.67	5.86	11.27
589												
590		1.11	1.04	1.16		0.40	0.40	0.75				
591						0.05	0.09	0.60				
592		0.75	1.24	1.75						3.34	15.85	21.88
593		1.07	0.51	0.45								
594												
595												
596		0.86	0.52	0.47		1.89	1.50	1.63		1.00	1.20	1.09
597						0.07	0.50	1.50		0.91	0.58	0.70
598												
599												
600		1.08	0.58	0.17								
601		0.72	1.21	1.01								
602		0.26	0.69	1.63								
603												
604												
605										0.42	0.34	0.33
606												
607												
608												
609												
610												
611						0.69	0.74	1.00				
612										0.49	1.57	1.41
613												
614												
615												
616		1.29	0.92	0.76								
617		0.89	0.95	2.25								
618		0.95	2.00	0.83								
619												
620										1.20	6.49	2.81
621		0.94	1.34	1.72								
622												
623		0.91	3.02	1.41								
624												

	iTRAQ Set 1				iTRAQ Set 2				iTRAQ Set 3			
Master N		NB1: NB2 (114:116)	GBM1: NB2 (115:116)	GBM2: NB2 (117:116)		GBM4: NB3 (114:115)	GBM3: NB3 (116:115)	NB4: NB3 (117:115)		NB5: NB6 (114:116)	GBM5: NB6 (115:116)	GBM6: NB6 (117:116)
623		0.91	3.02	1.41								
624												
625										1.98	2.51	2.36
626												
627										1.41	0.95	0.89
628		1.08	0.95	1.28								
629												
630						0.42	0.47	0.94				
631										1.21	1.31	2.63
632												
633												
634		0.96	0.88	1.46								
635												
636		1.41	0.67	0.52		0.74	0.95	0.71				
637						1.56	2.23	0.92				
638												
639		0.37	0.66	0.55								
640		1.05	0.60	0.76								
641												
642												
643												
644												
645												
646												
647												
648		1.31	0.95	1.20		1.07	0.83	1.33				
649		0.86	0.64	0.63								
650						1.45	1.20	1.28				
651		1.66	0.85	0.48						2.51	0.11	0.20
652		2.11	0.30	1.43								
653		1.13	0.38	0.36								
654												
655												
656												
657												
658										0.81	0.94	0.83
659												
660						1.18	1.53	0.94				
661		1.28	0.44	0.87		0.94	1.02	1.08				
662												
663										0.14	0.65	0.39
664												
665												
666										0.18	0.23	0.26
667										0.87	1.16	1.80
668										1.22	1.03	1.06

	iTRAQ Set 1				iTRAQ Set 2				iTRAQ Set 3			
Master N		NB1: NB2 (114:116)	GBM1: NB2 (115:116)	GBM2: NB2 (117:116)		GBM4: NB3 (114:115)	GBM3: NB3 (116:115)	NB4: NB3 (117:115)		NB5: NB6 (114:116)	GBM5: NB6 (115:116)	GBM6: NB6 (117:116)
669												
670										1.14	0.86	1.13
671		0.81	1.11	1.05								
672		1.13	0.86	0.73								
673						0.93	1.04	1.16				
674		1.15	0.92	1.05		0.97	1.01	1.10				
675												
676										0.91	0.98	1.17
677						1.22	1.03	0.84				
678										1.96	0.52	1.15
679						1.26	2.27	1.09				
680										0.66	0.78	0.66
681												
682		0.98	0.59	0.47								
683		1.08	0.94	0.71								
684										1.53	0.69	1.46
685						0.64	0.87	0.76				
686						0.56	0.60	1.87				
687										3.63	1.85	2.21
688						0.95	0.86	1.17				
689												
690		1.71	1.60	2.42								
691		0.88	0.51	0.69								
692						0.99	0.78	0.63				
693												
694										0.27	0.90	1.27
695												
696		1.29	0.97	0.97								
697												
698												
699												
700						0.49	0.68	1.18				
701										1.69	0.42	0.63
702												
703												
704						2.58	2.96	0.42				
705		1.16	1.06	1.11								
706										0.76	1.32	1.74
707		1.06	1.04	1.19								
708						1.00	0.56	0.97				
709												
710										0.15	1.16	1.51
711												
712												
713		0.80	1.38	1.49								
714		1.19	1.79	1.34								
715												
716		0.88	1.85	1.42								
717		1.08	0.74	1.00								
718		1.13	1.09	1.53								

	iTRAQ Set 1				iTRAQ Set 2				iTRAQ Set 3			
**Master N**		**NB1: NB2 (114:116)**	**GBM1: NB2 (115:116)**	**GBM2: NB2 (117:116)**		**GBM4: NB3 (114:115)**	**GBM3: NB3 (116:115)**	**NB4: NB3 (117:115)**		**NB5: NB6 (114:116)**	**GBM5: NB6 (115:116)**	**GBM6: NB6 (117:116)**
717		1.08	0.74	1.00								
718		1.13	1.09	1.53								
719												
720												
721										0.22	0.39	0.49
722												
723						6.31	6.98	0.79				
724										2.27	0.52	0.79
725		0.68	0.55	0.80								
726		0.86	1.00	0.59								
727												
728												
729		0.74	0.90	0.34								
730												
731						0.41	0.69	0.93				
732												
733												
734										0.72	0.76	0.89
735												
736										2.42	0.72	0.35
737												
738												
739												
740												
741												
742										0.73	2.09	0.99
743												
744												
745												
746		0.59	1.13	0.89								
747												
748												
749												
750												
751												
752										0.52	3.91	2.81
753												
754												
755						0.88	0.53	0.32				
756												
757												
758		1.05	0.78	1.60								
