# Supplementary figures and images for: Role of moesin in hyaluronan induced cell migration in glioblastoma multiforme
Source: Mol Cancer. 2013 Jul 15;12:74. doi: 10.1186/1476-4598-12-74 (PMC3718631; doi:10.1186/1476-4598-12-74)

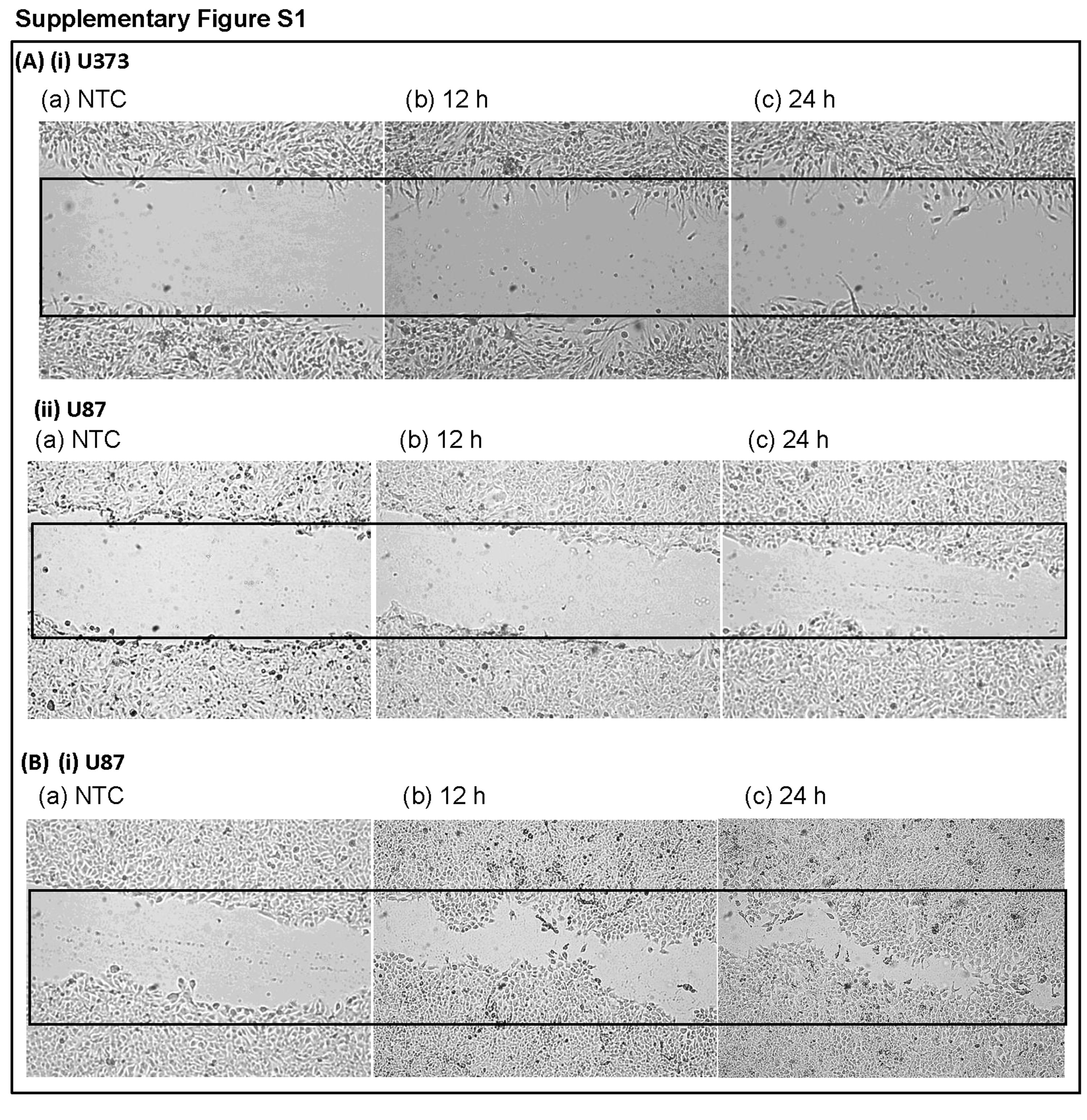

Supplement: Additional file 3: Figure S1 — Effect of HA-treatment on cell migration. Glioma cells (U373 and U87) were plated and treated with HA (100 μg/mL) or TNF-α (10 nM) for 6 - 24 h. All HA- / TNF-α treatments were given in DMEM only. Panel (A) shows no significant reduction in wound or number of cells in wound in either of the glioma cell lines (i) U373 and (ii) U87 when treated with HA (100 μg/mL) for 6 – 24 h as compared to no treatment controls (NTC); Panel shows data for (i) NTC; (ii)12 h and (iii) 24 h treatment; Panel (B) shows a significant reduction in wound and increase in num ber of cells in the wound of U87 cells treated with TNF-α (10 nM) as early as 12 h. Treatment with TNF-α (10 nM) served as positive control for wound healing assays (Original magnification X40). [file 1476-4598-12-74-S3.tiff]

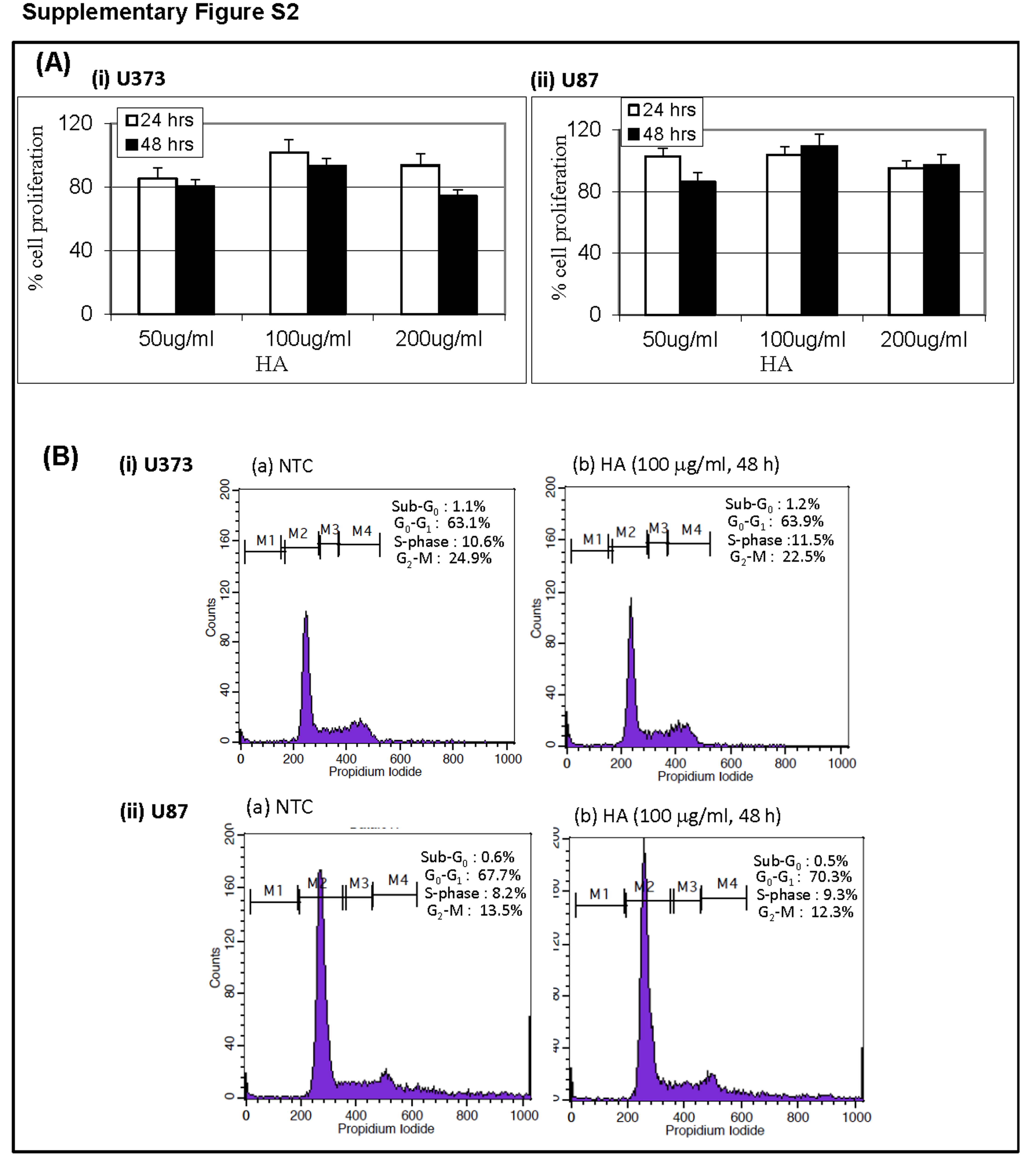

Supplement: Additional file 4: Figure S2 — (A) Cell viability assay. To determine the effect of HA-treatment on cell proliferation, GBM cells (U87 / U373) were plated in triplicates in 96-well plates in complete medium followed by treatment with varying concentrations of hyaluronan (HA) in DMEM only for 24 - 48 h. Panel A shows no significant difference in cell proliferation in glioma cells (U373 & U87) on treatment with HA (50 - 200 μg/mL) for 24 - 48 h; (B) Cell Cycle Analysis. For cell cycle analysis, both HA-treated and untreated no treatment control cells were collected, fixed and suspended in FACS buffer as described in Materials And Methods section. Panel B shows no significant difference in cell cycle in glioma cells (U373 & U87) on treatment with HA (100 μg/mL) for 48 h. [file 1476-4598-12-74-S4.tiff]
